# The DESTINIES Study: an online Delphi study to build international consensus on the medical conditions and procedures that confer immunosuppression and their respective COVID-19 risk profiles

**DOI:** 10.1016/j.eclinm.2025.103239

**Published:** 2025-05-05

**Authors:** Meredith Leston, José M. Ordóñez-Mena, Mark Joy, F.D Richard Hobbs, Simon de Lusignan, Benjamin W. Teh, Ingrid de Groot, Iain McInnes, Hana M. El Sahly, John Isaacs, Monique Andersson, Francois Raffi, Wei Shen Lim, Richard Conway, Stefan Siebert, Iain Buchan, Martin Underwood, David Lowe, Michael Hoerger, Christopher E.M. Griffiths, Alessia Alunno, Lennard Y.W. Lee, the DESTINIES Consortium

**Affiliations:** a Nuffield Department of Primary Care Health Sciences, University of Oxford, Oxford, United Kingdom; b Department of Infectious Diseases, Peter MacCallum Cancer Centre, 305 Grattan Street, Melbourne, VIC, 3000, Australia; c Spierziekten Nederland, Dutch Myositis Working Group, Lt.Gen van Heutszlaan 6, 3743JN, Baarn, Netherlands; d MVLS College Office, Wolfson Medical School Building, University of Glasgow, University Avenue, Glasgow, United Kingdom; e Departments of Molecular Virology and Microbiology and Medicine, Baylor College of Medicine, Houston, TX, USA; f William Leech Building, Medical School, Framlington Place, Newcastle Upon Tyne, United Kingdom; g Nuffield Division of Clinical Laboratory Science, Radcliffe Department of Medicine, University of Oxford, Oxford, United Kingdom; h Department of Infectious and Tropical Diseases and CIC 1413, INSERM, University Hospital, Nantes, 44093, France; i Nottingham University Hospitals, City Hospital Campus, Hucknall Road, Nottingham, United Kingdom; j Robert Mayne Clinic & Rheumatology Day Centre, St James' Hospital, Dublin, Ireland; k School of Infection & Immunity, University of Glasgow, University Place, Glasgow, United Kingdom; l Institute of Population Health, Waterhouse Building, Block B Brownlow Street, Liverpool, United Kingdom; m Warwick Clinical Trials Unit, Warwick Medical School, University of Warwick, Coventry, United Kingdom; n UCL Institute of Immunity & Transplantation, The Pears Building, Pond Street, London, United Kingdom; o Tulane Cancer Center, Tulane University, 1700 Tulane Ave, New Orleans, LA, 70112, USA; p Dermatology Centre, Hope Hospital, University of Manchester, Manchester, United Kingdom; q Department of Life, Health and Environmental Sciences, University of L'Aquila, Internal Medicine and Nephrology Division, ASL-1 Avezzano-Sulmona-L'Aquila, L'Aquila, Italy; r Department of Oncology, University of Oxford, Old Road Campus Research Building, Roosevelt Drive, Oxford, OX3 7DQ, United Kingdom

**Keywords:** Vaccine, Immunosuppressed, Immunosuppression, Consensus, Delphi, Public health, Policy, Digital health, COVID-19, Policymaking, Disease surveillance, Pharmacovigilance, Inoculation, Personalised medicine, Precision health, Inclusion health

## Abstract

**Background:**

The lack of international consensus on defining and categorising immunosuppression has undermined disease surveillance and patient care, particularly during the COVID-19 pandemic. To address this, a global expert panel was recruited to join the eDElphi STudy to fully defiINe and COVID-risk stratify ImmunosupprESsion (DESTINIES) and develop a COVID risk-stratified digital phenotype for ‘adult immunosuppression’ (the DESTINIES phenotype).

**Methods:**

Panellists were presented with all medical diagnoses and procedures cited in prevailing immunosuppressed definitions; they evaluated their appropriateness for the DESTINIES phenotype and their risks for severe COVID-19 outcomes through anonymous online questionnaires and discussion. Panel agreement with a series of clinical statements were also assessed; statements incorporated longstanding disputes, including variables that could reverse immunosuppression. Each round of data collection informed and refined a draft phenotype until final ratification. This study was active between May and September 2024.

**Findings:**

Sixty-four experts from four continents and 12 international agencies completed two rounds of consensus questionnaire, a discussion group and ratifying vote. Panellists identified candidates posing higher (e.g. Transplantation, Primary Immunodeficiency) and lower COVID-19 risk (e.g. Anorexia nervosa, Cerebral spinal fluid leak) but disagreed on the categorisation of others (e.g. Asplenia, Immune-mediated Inflammatory Disease). Consensus was reached on ten clinical statements, notably removing Drug-managed HIV and Cancer remission from consideration as immunosuppressed. The DESTINIES phenotype was ratified with near unanimous support (94%) for implementation in surveillance.

**Interpretation:**

Pending validation, the DESTINIES phenotype provides a clinically meaningful, internationally ratified and digitally practical method for identifying and COVID-19 risk-stratifying adult immunosuppressed patients in healthcare data.

**Funding:**

This work was funded by the UK 10.13039/501100000265Medical Research Council and EMIS Health.

**Research in context**

**Evidence before this study**

Before this study was conducted, there was substantial international disagreement over the medical diagnoses and procedures that would confer immunosuppression and their respective vulnerability to severe COVID-19 outcomes. These disagreements have fragmented the global research and public policy landscape. DESTINIES Study panellists were made aware of this in their pre-read packs, incorporating study background, contemporaneous information on COVID-19 vaccine benefit-risk within the immunosuppressed (something insufficiently differentiated) and introductions to both the online Delphi method and digital phenotyping.

**Added value of this study**

This study convened a panel of 64 world-leading experts at the intersection of immunology, vaccinology and infectious disease. This panel worked together to surface a COVID-19 specific, internationally aligned and digitally practical phenotype for ‘adult immunosuppression’: the DESTINIES Phenotype. During this process, consensus was also achieved on the diagnoses and procedures that carry higher and lower risks for severe COVID-19 outcomes within the immunosuppressed spectrum and on various clinical statements that were previously longstanding sources of disagreement.

**Implications of all the available evidence**

Subject to validation in real-world data, these outputs can enhance COVID-19 disease surveillance and produce the differentiated intelligence that is of benefit for targeting future care. Their generalisability to other infections can also be tested. The expansiveness of the DESTINIES phenotype and its risk-banding communicate the scale of vulnerability in the general population and the need to allocate COVID-19 resources on a needs-basis. In this, the diagnoses the panel confirmed as at higher risk for severe COVID-19 outcomes within the immunosuppressed spectrum should be prioritised. This exercise is also the first to approve the removal of Cancer in Remission and Drug-managed HIV from characterisations of immunosuppression—the latter of which is a major milestone in the global fight against HIV.

## Introduction

Internationally, there is considerable disagreement over the medical diagnoses and procedures that confer immunosuppressed status and how this clinical spectrum might be risk-categorised.^1^ Even subtle differences in definitions affect population sizing: depending on criteria used, the immunosuppressed spectrum can constitute anywhere from 2% to nearly 7% of the general population.^2^^,^^3^ Such inconsistency fragments international research, disease surveillance and policymaking as findings from discordant populations cannot be easily compiled, compared or actioned.^4^ This, in turn, undermines stated ambitions for targeted patient care.^4^

The COVID-19 pandemic typifies these issues and provides a unique opportunity for improvement. Despite global motivation to protect the clinically vulnerable, conflicting characterisations of immunosuppression created international rifts in the patients selected for enhanced monitoring and management.^5^ That said, thanks to the ubiquity of community testing,^6^ immunosuppressed COVID-19 outcomes have been documented with unprecedented detail^7^; even rare and complex conditions have been centred in COVID-19 infection and vaccine research.^8^ This is a departure from the convention of aggregating immunosuppressed outcomes^9^ or selectively reporting those of dominant subgroups.^5^

The eDElphi STudy to fully defiINe and COVID-risk stratify ImmunosupprESsion (DESTINIES Study) responded to these developments, utilising COVID-19 prognosis as a common denominator to segment the entire immunosuppressed patient spectrum. Its objectives were: 1) to achieve consensus on the medical diagnoses and procedures that confer immunosuppressed status in adults and their respective prognoses for COVID-19 and 2) to convert this information into a COVID-19 specific, internationally aligned, risk-stratified and digitally practical taxonomy of ‘adult immunosuppression’—otherwise known as a digital phenotype—for easy integration within electronic medical records (EMRs). If realised and validated in real-world data, the latter objective has the potential to improve the resolution of COVID-19 surveillance for this diverse risk group and thereby inform COVID prevention and resource allocation going forwards.

## Methods

### Recruitment

The design, implementation, write-up and dissemination of this eDelphi study was directed by a steering group. This comprised of University of Oxford researchers, statisticians, external research collaborators and a patient research champion with lived experience of immunosuppression. All questions put to the DESTINIES panel, as well as the statistical approach that supported their analysis, were developed by this steering group.

As per the timeline provided (Supplementary Materials), the DESTINIES Study was active between May and September 2024. A study protocol, published prior to study start date,^10^ justified the use of the eDelphi design and detailed recruitment criteria, entities approached for panellist recruitment (provided in full in Supplementary Materials, inclusive of World Health Organisation [WHO], Coalition for Epidemic Preparedness Innovations [CEPI] and European Medicines Agency [EMA] vaccine decision-making bodies), consensus rounds and the consensus level (≥75% panellist agreement) based on Diamond and colleagues’ recommendation.^11^

Only individuals with expertise in adult immunology, vaccinology or infectious disease were considered for recruitment. Those with exclusively paediatric expertise were not considered. Recruitment was purposive, aiming to secure a globally representative and gender-balanced panel of at least 50 panellists in anticipation of possible attrition. Efforts were made to ensure this panel offered expertise on all candidate diagnoses and procedures assessed. To maximise recruitment, eligible individuals who were unable to commit to the whole study period were invited to nominate alternates.

Those interested in participating were provided with a consent form and pre-read pack (Supplementary Materials) to establish baseline knowledge for panellists from various clinical backgrounds. The latter included study rationale, executive summaries of comparative literature on COVID vulnerability^12^ and vaccine benefit risk amongst the immunosuppressed^13^^,^^14^ and introductions to digital phenotyping and consensus building.^15^

### Ethical approval and consent

This study was entered into a formal ethical review process at the University of Oxford; however, both the University's Research Governance Ethics and Assurance Team and the Joint Research Office Study Classification Group determined that this was unnecessary. Categorising this work as “pre-research”, “priority setting”, or “survey”, both entities confirmed that neither formal sponsorship or research ethics review would be required.

As above, prospective panellists could only participate in the DESTINIES Study once they had completed and returned their consent form (Supplementary Materials).

### Questionnaires

Confirmed panellists were provided with unique ID numbers and a link to submit their first DESTINIES questionnaire. All questionnaires were hosted on Google Forms (Alphabet, Inc.), chosen due to its customisability, anonymity, advanced security measures and automated spreadsheet generation. Paper-based alternatives were offered to any panellists who felt uncomfortable using this interface.

As detailed in Supplementary Materials, questionnaires consisted of closed and free text items. These assessed the appropriateness of candidate diagnoses and procedures for inclusion in a COVID-19 specific phenotype for ‘adult immunosuppression’ and their respective levels of risk for severe infection outcomes—assessed internally (Higher vs Lower COVID risk immunosuppressed) and against a baseline (excess vulnerability compared to the immunocompetent).

Candidates are listed with their sources in Supplementary Materials. These terms were extracted from Green Book Chapter 14a and comparable international classifications or definitions of immunosuppression in adults. Terms without dedicated diagnostic codes were operationalised (e.g. Anorexia nervosa and Stunting as operationalisations of Malnutrition, cited by the National Institute of Cancer^16^). For comprehensiveness, Wider determinants for immunosuppression, including Pregnancy,^17^ Chronic stress^18^ and Sleep deprivation,^19^ were appraised. Panellists had the opportunity to recommend candidate diagnoses in the first round of consensus building (see ‘*Panellist recommendation’*). In Round 2, these suggestions had their appropriateness for inclusion evaluated in turn; those that were deemed appropriate for inclusion by ≥75% of panellists were carried forward for phenotyping.

Medication classes were excluded from consideration to align with our secondary objective of producing a digitally practical COVID risk-stratified phenotype for ‘adult immunosuppression’. Prescription data are insufficiently codified in EMRs to support medication-inclusive phenotyping^20^ as essential details including drug type, brand, dose, polypharmacy, patient weight and prescription timing or discontinuation are not reliably available.^21^ Medical diagnoses and procedures are better codified by comparison.^22^ However, a series of clinical statements were presented to panellists to build consensus on medication variables that could be operationalised without prescription data (e.g. Drug-managed HIV, inferable within EMRs thanks to the near universal uptake of antiretroviral medications in developed contexts^23^). These statements also aimed to settle longstanding disputes within the immunosuppressed literature, including the exchangeability of key immunosuppressed terms, the universality of patient prioritisation and the medication time and dose thresholds that confer immunosuppression.

Questionnaire items were adapted or refined between rounds if panellists provided feedback that the steering group judged in keeping with study objectives. For example, in later versions, reference populations were made explicit, the phrasing and operationalisation of candidate diagnoses were clarified, and a distinction was made between low dose (<20 mg per day) and very low dose (<10 mg per day) prednisolone in clinical consensus statements. Apart from ‘Higher vs Lower risk’ categorisations, all questionnaire items were Likert based. Answers at Likert poles (e.g. ‘Strongly agree’ and ‘Somewhat agree’ vs ‘Strongly disagree’ and ‘Somewhat disagree’) were combined to assess consensus direction. Middle Likert options and free text boxes allowed panellists to report any uncertainty they had in their answers. Panellists could not skip questions and were instructed not to share any identifiable information about themselves or patients while completing free text items.

### Phenotyping

Between rounds of consensus building and in preparation for final discussion, panellists were presented with the results of the preceding round and draft COVID-specific phenotypes for ‘adult immunosuppression’ that the steering group felt best reflected this data. Initially two types of phenotypes were presented to panellists: COVID risk-categorised (a Higher vs Lower COVID-19 risk binary within the immunosuppressed spectrum based on risk-allocation data) and COVID risk-stratified (a multi-level COVID-19 risk hierarchy based on vulnerability compared to the immunocompetent data).

As a binary phenotype, Higher vs Lower risk-categorisations were first majoritarian but demarcated the diagnoses within each category that exceeded the ≥75% consensus level. However, panellists rejected this approach and the ≥75% standard was instated for risk-categorisation by the second round of consensus building - creating a ‘*Contested’* category for those that did not meet this threshold in so doing. The steering group's initial labelling of these contested terms as ‘*Moderate risk’* was also rejected; panellists requested they be reported as ‘*No consensus’*.

Meanwhile, all drafts of the risk-stratified phenotype aimed to create phenotype levels with common clinical or anatomical denominators. This was balanced with consideration for population size; unbalanced levels would affect the viability of comparative analysis during implementation. As directed by panellists and steering group members, cluster analysis on candidate diagnoses' vulnerability scores provided an additional check on the accuracy of the last draft of the risk-stratified phenotype. Hierarchical cluster analysis, based on panel vulnerability scores, Euclidian distance for dissimilarity and Ward's minimum variance for clustering, assessed candidates' similarity. A dendrogram illustrated these shared vulnerability profiles; candidates positioned closest together were most alike in terms of panel-appraised vulnerability and, thereby, appropriate for inclusion in the same phenotype level.

Final discussion groups were arranged to give panellists the opportunity to provide direct and more extensive feedback on these draft phenotypes, the evidence they were based upon as well as the clinical statements that had not yet achieved consensus. Those unable to attend were given the option to submit a written contribution. Meetings were hosted and recorded on Zoom (Zoom Video Communications Inc); to retain their anonymity, panellists disabled their cameras and displayed their assigned IDs. Meeting transcripts and summaries informed the final DESTINIES phenotype that panellists then ratified via Google Form.

### Role of funding source

This work was funded through a joint award between the UK Medical Research Council and EMIS Health. Neither funder was involved in the design, delivery or publication of this work in any capacity.

## Results

### Recruitment

64 panellists were successfully recruited to the DESTINIES Consortium; their characteristics are summarised in Supplementary Materials. Expertise across the whole immunosuppressed patient spectrum was represented in this panel. There was over-representation amongst rheumatologists and those working in Europe and North America; the African and South American continents were largely and completely missing by comparison.

Round-by-round panellist attrition is also provided in Supplementary Materials. There was substantial withdrawal between online data collection and final discussion – while this did not significantly affect the evidence base that informed the DESTINIES phenotype, this did reduce the pool of panellists that were able to ratify it.

### Questionnaires

Data from every questionnaire presented to panellists are tabulated in Supplementary Materials. Final data on candidate diagnoses' 1) appropriateness for inclusion in a COVID risk-stratified digital phenotype for ‘adult immunosuppression’, 2) excess vulnerability to severe COVID-19 outcomes compared to the immunocompetent and 3) categorisations into higher vs lower risk for severe COVID-19 outcomes within the immunosuppressed patient spectrum are provided below in Table 1, Table 2, Table 3 respectively. Tables 1 and 2 present data by clinical category to facilitate comparisons between diagnoses with common traits.

**Table 1:** Final panel determinations on the appropriateness of candidate diagnoses for a COVID-specific phenotype of ‘adult immunosuppression’.

	Diagnosis	Not appropriate %	Somewhat inappropriate %	Unsure %	Somewhat appropriate %	Appropriate %	Sum Inappropriate %	Sum Appropriate %	Determination
Malignancy	Actively treated malignancy	0.0	0.0	1.7	20.0	78.3	0.0	98.3	*Appropriate*
	Haematological malignancies	0.0	0.0	0.0	5.0	95.0	0.0	100.0	*Appropriate*
	Generalised malignancies (metastasis)	0.0	1.7	6.7	26.7	65.0	1.7	91.7	*Appropriate*
	Solid tumours	0.0	5.0	13.3	41.7	40.0	5.0	81.7	*Appropriate*
Transplantation	Bone marrow transplantation	0.0	0.0	0.0	3.3	96.7	0.0	100.0	*Appropriate*
	Islet transplantation	1.7	3.3	16.7	33.3	45.0	5.0	78.3	*Appropriate*
	Multi-organ transplantation	0.0	0.0	1.7	1.7	96.7	0.0	98.3	*Appropriate*
	Solid organ transplantation	0.0	0.0	0.0	3.3	96.7	0.0	100.0	*Appropriate*
	Stem cell transplantation	0.0	0.0	0.0	1.7	98.3	0.0	100.0	*Appropriate*
Primary & Acquired Immunodeficiency	AIDS-defining illness	0.0	1.7	0.0	18.3	80	1.7	98.3	*Appropriate*
	Down Syndrome	0.0	11.7	21.7	45.0	21.7	11.7	66.7	Contested
	Drug-managed HIV	13.3	38.3	21.7	20.0	6.7	51.7	26.7	Contested
	Genetic disorders affecting the immune system	0.0	3.3	3.3	18.3	75.0	3.3	93.3	*Appropriate*
	HIV infection all stages	5.0	46.7	11.7	26.7	10.0	51.7	36.7	Contested
	Underlying aberrant immunity	0.0	1.7	1.7	28.3	68.3	1.7	96.7	*Appropriate*
	Untreated HIV	1.7	3.3	3.3	31.7	60.0	5	91.7	*Appropriate*
Immune mediated inflammatory disorders	Autoimmune skin disorders	0.0	25.0	31.7	36.7	6.7	25.0	43.3	Contested
	Endocrine autoimmune disorders	3.3	35.0	25.0	31.7	5.0	38.3	36.7	Contested
	Gastrointestinal autoimmune disorders	0	18.3	18.3	53.3	10	18.3	63.3	Contested
	Hematologic autoimmune disorders	1.7	13.3	26.7	43.3	15	15.0	58.3	Contested
	Myalgic encephalomyelitis/chronic fatigue syndrome	45	21.7	28.3	3.3	1.7	66.7	5.0	Contested
	Neurologic autoimmune disorders	0.0	16.7	21.7	51.7	10.0	16.7	61.7	Contested
	Ophthalmologic autoimmune disorders	6.7	38.3	25.0	23.3	6.7	45.0	30.0	Contested
	Renal autoimmune disorders	0.0	10.0	16.7	50.0	23.3	10.0	73.3	Contested
	Rheumatologic disorders	0.0	15.0	8.3	58.3	18.3	15.0	76.7	*Appropriate*
Disorders affecting haematopoiesis	Aplastic anaemia	1.7	13.3	8.3	33.3	43.3	15.0	76.7	*Appropriate*
	Asplenia (anatomic/functional)	8.3	10.0	11.7	35.0	35.0	18.3	70.0	Contested
	Chronic kidney disease	1.7	15.0	18.3	46.7	18.3	16.7	65.0	Contested
	Dialysis	0.0	6.7	6.7	45.0	41.7	6.7	86.7	*Appropriate*
	Liver disease (including cirrhotic)	0.0	16.7	11.7	50.0	21.7	16.7	71.7	Contested
	Nephrotic disease	1.7	15.0	15.0	43.3	25.0	16.7	68.3	Contested
	Sickle cell disease	8.3	16.7	23.3	33.3	18.3	25.0	51.7	Contested
Lung disorders	Chronic obstructive pulmonary disease	6.7	23.3	13.3	43.3	13.3	30.0	56.7	Contested
	Cystic fibrosis	5.0	16.7	8.3	35.0	35.0	21.7	70.0	Contested
Anatomical barrier defects	Burn injuries	11.7	28.3	20.0	31.7	8.3	40.0	40.0	Contested
	Cerebrospinal fluid leak	18.3	26.7	28.3	15.0	11.7	45.0	26.7	Contested
	Cochlear implant	31.7	31.7	20.0	8.3	8.3	63.3	16.7	Contested
	Skin graft recipients	16.7	23.3	26.7	26.7	6.7	40.0	33.3	Contested
Wider determinants of immunosuppression	Anorexia nervosa	41.67	15	23.3	16.7	3.3	56.7	20.0	Contested
	Chronic stress	58.3	18.3	15.0	6.7	1.7	76.7	8.3	*Inappropriate*
	Malnutrition	30.0	16.7	21.7	26.7	5.0	46.7	31.7	Contested
	Obesity	18.3	33.3	16.7	26.7	5.0	51.7	31.7	Contested
	Pregnancy	16.7	20.0	10.0	41.7	11.7	36.7	53.3	Contested
	Preterm births	3.3	20.0	21.7	31.7	23.3	23.3	55.0	Contested
	Sleep deprivation	60.0	18.3	16.7	3.3	1.7	78.3	5.0	*Inappropriate*
	Stunting	45.0	25.0	23.3	5.0	1.7	70.0	6.7	Contested
	Type 2 diabetes mellitus	10.0	35.0	16.7	31.7	6.7	45.0	38.3	Contested

**Table 2:** Candidate diagnoses’ excess vulnerability to severe COVID-19 outcomes compared to the immunocompetent.

	Diagnosis	No additional vulnerability %	Slightly elevated vulnerability %	Significantly elevated vulnerability %	High vulnerability %	Extremely high vulnerability %	Sum minimal additional vulnerability %	Sum major additional vulnerability %	Determination
Malignancy	Actively treated malignancy	1.7	13.3	16.7	40.0	28.3	15.0	68.3	Contested
	Haematological malignancies	0.0	8.3	21.7	40.0	30.0	8.3	70.0	Contested
	Generalised malignancies (metastasis)	0	1.7	6.7	26.7	65.0	1.7	91.7	*Major additional vulnerability*
	Solid tumours	0	15	31.7	36.7	16.7	15.0	53.3	Contested
Transplantation	Bone marrow transplantation	0.0	0.0	6.7	8.3	85.0	0.0	93.3	*Major additional vulnerability*
	Islet transplantation	5.0	8.3	28.3	33.3	25.0	13.3	58.3	Contested
	Multi-organ transplantation	0.0	0.0	5.0	10.0	85.0	0.0	95.0	*Major additional vulnerability*
	Solid organ transplantation	0.0	0.0	10.0	13.3	76.7	0.0	90.0	*Major additional vulnerability*
	Stem cell transplantation	0.0	0.0	5.0	10.0	85.0	0.0	95.0	*Major additional vulnerability*
Primary & Acquired Immunodeficiency	AIDS-defining illness	0.0	3.3	21.7	26.7	48.3	3.3	75.0	*Major additional vulnerability*
	Down Syndrome	5.0	21.7	41.7	21.7	10.0	26.7	31.7	Contested
	Drug-managed HIV	38.3	43.3	15.0	3.3	0.0	81.7	3.3	*Minimal additional vulnerability*
	Genetic disorders affecting the immune system	0.0	3.3	8.3	30.0	58.3	3.3	88.3	*Major additional vulnerability*
	HIV infection all stages	5.0	48.3	21.7	20.0	5.0	53.3	25.0	Contested
	Underlying aberrant immunity	0.0	11.7	10.0	36.7	41.7	11.7	78.3	*Major additional vulnerability*
	Untreated HIV	1.7	13.3	26.7	41.7	16.7	15.0	58.3	Contested
Immune mediated inflammatory disorders	Autoimmune skin disorders	8.3	60.0	21.7	8.3	1.7	68.3	10.0	Contested
	Endocrine autoimmune disorders	16.7	50.0	23.3	10.0	0.0	66.7	10.0	Contested
	Gastrointestinal autoimmune disorders	6.7	51.7	26.7	15.0	0.0	58.3	15.0	Contested
	Hematologic autoimmune disorders	8.3	31.7	36.7	18.3	5.0	40.0	23.3	Contested
	Myalgic encephalomyelitis/chronic fatigue syndrome	70.0	26.7	1.7	1.7	0.0	96.7	1.7	*Minimal additional vulnerability*
	Neurologic autoimmune disorders	6.7	45.0	33.3	15.0	0.0	51.7	15.0	Contested
	Ophthalmologic autoimmune disorders	28.3	51.7	13.3	6.7	0.0	80.0	6.7	*Minimal additional vulnerability*
	Renal autoimmune disorders	5.0	23.3	38.3	28.3	5.0	28.3	33.3	Contested
	Rheumatologic disorders	3.3	26.7	43.3	23.3	3.3	30.0	26.7	Contested
Disorders affecting haematopoiesis	Aplastic anaemia	10.0	26.7	20.0	36.7	6.7	36.7	43.3	Contested
	Asplenia (anatomic/functional)	20.0	30.0	23.3	21.7	5.0	50.0	26.7	Contested
	Chronic kidney disease	1.7	28.3	40.0	30.0	0.0	30.0	30.0	Contested
	Dialysis	0.0	21.7	28.3	35.0	15.0	21.7	50.0	Contested
	Liver disease (including cirrhotic)	8.3	33.3	36.7	18.3	3.3	41.7	21.7	Contested
	Nephrotic disease	3.3	35.0	33.3	26.7	1.7	38.3	28.3	Contested
	Sickle cell disease	13.3	35.0	28.3	21.7	1.7	48.3	23.3	Contested
Lung disorders	Chronic obstructive pulmonary disease	10.0	21.7	35.0	25.0	8.3	31.7	33.3	Contested
	Cystic fibrosis	8.3	23.3	35.0	20.0	13.3	31.7	33.3	Contested
Anatomical barrier defects	Burn injuries	43.3	28.3	20.0	8.3	0.0	71.7	8.3	Contested
	Cerebrospinal fluid leak	55.0	28.3	6.7	8.3	1.7	83.3	10.0	*Minimal additional vulnerability*
	Cochlear implant	66.7	16.7	6.7	8.3	1.7	83.3	10.0	*Minimal additional vulnerability*
	Skin graft recipients	41.7	33.3	10.0	13.3	1.7	75.0	15.0	*Minimal additional vulnerability*
Wider determinants of immunosuppression	Anorexia nervosa	45.0	41.7	11.7	1.7	0.0	86.7	1.7	*Minimal additional vulnerability*
	Chronic stress	63.3	31.7	3.3	1.7	0.0	95.0	1.7	*Minimal additional vulnerability*
	Malnutrition	31.7	46.7	16.7	5.0	0.0	78.3	5.0	*Minimal additional vulnerability*
	Obesity	21.7	43.3	18.3	13.3	3.3	65.0	16.7	Contested
	Pregnancy	6.7	43.3	23.3	25.0	1.7	50.0	26.7	Contested
	Preterm births	16.7	38.3	30.0	13.3	1.7	55.0	15.0	Contested
	Sleep deprivation	70.0	26.7	1.7	1.7	0.0	96.7	1.7	*Minimal additional vulnerability*
	Stunting	61.7	31.7	5.0	1.7	0.0	93.3	1.7	*Minimal additional vulnerability*
	Type 2 diabetes mellitus	15.0	48.3	20.0	16.7	0.0	63.3	16.7	Contested

**Table 3:** Panel categorisations for higher vs lower risk for severe COVID-19 outcomes within the immunosuppressed patient spectrum.

Diagnosis	Higher Risk (n)	Higher Risk (%)	Lower Risk (n)	Lower Risk (%)	Determination
Bone marrow transplant	59	98.3	1	1.7	Higher Risk
Haematological malignancies	59	98.3	1	1.7	Higher Risk
Multi-organ transplant	59	98.3	1	1.7	Higher Risk
Solid organ transplant	59	98.3	1	1.7	Higher Risk
Stem cell transplant	59	98.3	1	1.7	Higher Risk
Actively treated malignancies	56	93.3	4	6.7	Higher Risk
AIDS-defining illness	56	93.3	4	6.7	Higher Risk
Genetic diseases (PIDs)	56	93.3	4	6.7	Higher Risk
Untreated HIV	55	91.7	5	8.3	Higher Risk
Generalised malignancies (metastasis)	54	90.0	6	10.0	Higher Risk
Underlying aberrant immunity	53	88.3	7	11.7	Higher Risk
Islet transplant	52	86.7	8	13.3	Higher Risk
Dialysis	50	83.3	10	16.7	Higher Risk
Renal autoimmune	46	76.7	14	23.3	Higher Risk
Aplastic anaemia	45	75.0	15	25.0	Higher Risk
Solid tumours	45	75.0	15	25.0	Higher Risk
Chronic kidney disease	44	73.3	16	26.7	No consensus
Rheumatological disorders	44	73.3	16	26.7	No consensus
Nephrotic syndrome	43	71.7	17	28.3	No consensus
Chronic Obstructive Pulmonary Disease	42	70.0	18	30.0	No consensus
Cystic fibrosis	42	70.0	18	30.0	No consensus
Haematologic autoimmune disorders	41	68.3	19	31.7	No consensus
Down syndrome	39	65.0	21	35.0	No consensus
Liver disease (including cirrhotic)	39	65.0	21	35.0	No consensus
Sickle cell disease	39	65.0	21	35.0	No consensus
HIV infection (all levels)	37	61.7	23	38.3	No consensus
Asplenia (functional or anatomic)	36	60.0	24	40.0	No consensus
Neurologic autoimmune disorders	36	60.0	24	40.0	No consensus
Pregnancy	33	55.0	27	45.0	No consensus
Preterm births	33	55.0	27	45.0	No consensus
Obesity	29	48.3	31	51.7	No consensus
Gastrointestinal autoimmune disorders	28	46.7	32	53.3	No consensus
Autoimmune skin disorders	23	38.3	37	61.7	No consensus
Endocrine autoimmune disorders	22	36.7	38	63.3	No consensus
Type 2 Diabetes	20	33.3	40	66.7	No consensus
Burn injuries	17	28.3	43	71.7	No consensus
Malnutrition	17	28.3	43	71.7	No consensus
Skin grafts	17	28.3	43	71.7	No consensus
Ophthalmologic autoimmune disorders	16	26.7	44	73.3	No consensus
Cerebrospinal fluid leak	14	23.3	46	76.7	Lower Risk
Anorexia nervosa	12	20.0	48	80.0	Lower Risk
Cochlear implants	11	18.3	49	81.7	Lower Risk
Stunting	8	13.3	52	86.7	Lower Risk
Drug-managed HIV	7	11.7	53	88.3	Lower Risk
Chronic stress	6	10.0	54	90.0	Lower Risk
Sleep deprivation	6	10.0	54	90.0	Lower Risk
ME/CFS	4	6.7	56	93.3	Lower Risk

There was persistent disagreement over the appropriateness of almost all candidates for immune mediated inflammatory disorders, anatomical barrier defects and wider determinants of immunosuppression. Candidates that met ≥75% panel agreement as inappropriate for inclusion were removed from final phenotypes; this included Chronic Stress and Sleep Deprivation. In keeping with our diagnosis and procedural approach to phenotyping, Actively Treated Malignancy patients were also dropped from drafts. Synonymous or overlapping candidates were also removed: HIV infection all stages was made redundant by the retention of Drug-managed HIV, Untreated HIV and AIDS-defining illness, for example. Finally, no candidate diagnosis suggested by panel members in Round 1 (Obesity, Skin graft recipients, Myalgic encephalomyelitis/chronic fatigue syndrome [ME/CFS), Liver disease [including cirrhotic), Cystic fibrosis, Preterm births) met the ≥75% consensus level for appropriateness; this meant they were not carried forward for phenotyping.

Despite the superficial agreement between panellists’ appraisals of appropriateness and vulnerability, there are important areas of departure. For example, heterogeneous vulnerability ratings in malignancy and transplantation patients contrasted with near-universal appropriateness.

Panel 1 distinguishes between the clinical statements that did and did not achieve panel consensus.

Panel 1 **DESTINIES Consortium appraisals of general and dependency clinical statements**

**Of the clinical statements presented to panellists, consensus was achieved on the following:**

General clinical statements:

≥75% AGREE.

• *‘Immunosuppression is poorly defined’* (92%)
• *‘COVID care for the immunosuppressed—including vaccination and the release of antivirals and other resources—is overly one size fits all’* (82%)

≥75% DISAGREE

• *‘COVID-19 no longer poses a real risk to the immunosuppressed’* (97%)
• *‘Vulnerability to COVID-19 is similar for all immunosuppressed subgroups’* (97%)

Dependency clinical statements:

≥75% AGREE.

• *‘A patient would no longer be considered immunosuppressed if…*
  o *Their immunosuppressive regimen was discontinued more than 3 years ago’* (90%)
  o *Their HIV was drug managed’* (82%)
  o *Their immunosuppressive regimen was discontinued more than 1 year ago’* (82%)
  o *Their cancer was in remission’* (77%)

≥75% DISAGREE.

• *‘A patient would no longer be considered immunosuppressed if…*
  o *They had been on immunosuppressive treatment for less than 6 months’* (85%)
  o *Their cancer was untreated’* (77%)

However, consensus was not achieved on the following:

General clinical statements:

• *‘Our current clinical definition for immunosuppression is too expansive’*
• *‘Our current clinical definition for immunosuppression is not expansive enough’*
• *‘The terms ‘immunosuppression’ and ‘immunocompromised’ can be used interchangeably’*
• *‘The needs of the immunosuppressed are sufficiently prioritised in times of public health emergency’*
• *‘It is easy to pick out the immunosuppressed subgroups at most risk for COVID-19’*
• *‘The immunosuppressed subgroups that are at most risk for COVID-19 are the same subgroups at most risk for other infectious diseases (Respiratory syncytial virus, influenza, bacterial pneumonia etc.)’*

Dependency clinical statements:

• *‘A patient would no longer be considered immunosuppressed if…*
  o *Their cancer is classed as Early Stage (Stage I)’*
  o *Their inflammatory disease is currently untreated’*
  o *Their immunosuppressive regimen was discontinued more than 6 months ago’*
  o *They are on low dose immunosuppressives (equivalent of<20 mg of prednisolone)’*
  o *They are on very low dose immunosuppressives (equivalent of<10 mg of prednisolone)’*

### Phenotyping

A summary document of DESTINIES Final Discussion Groups is provided in Supplementary Files; a total of twelve were hosted to fit within sociable hours of all attending panellists. All iterations of draft Risk-Categorised and Risk-Stratified phenotypes for adult immunosuppression and their evaluation data are also provided within Supplementary Materials.

**Fig. 1:**
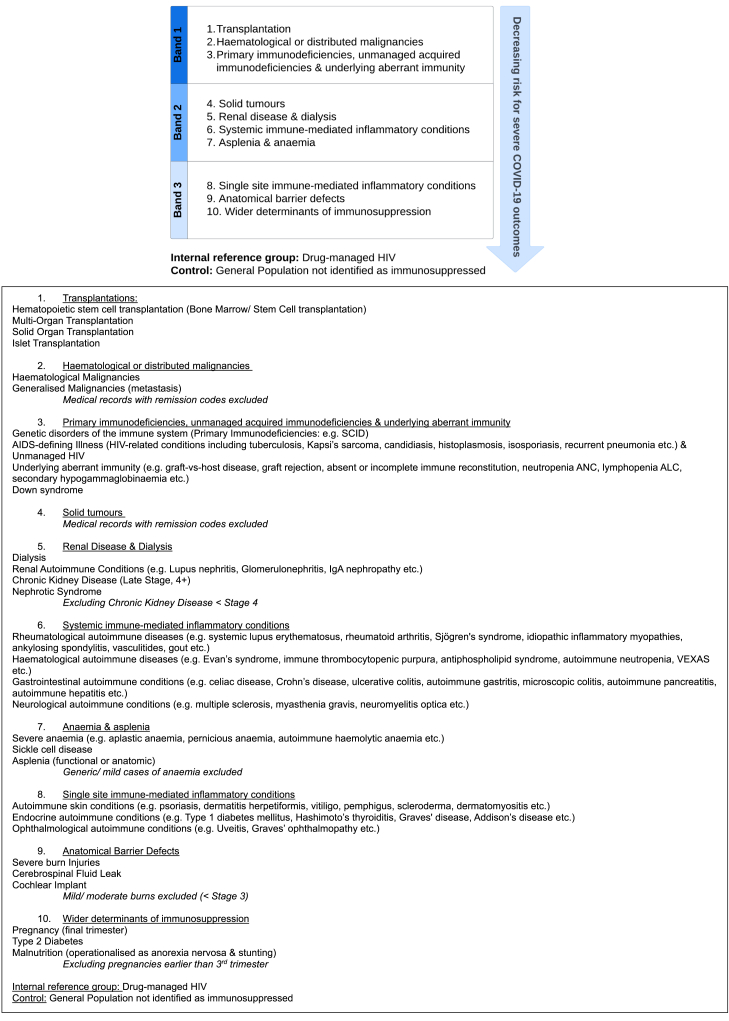
The DESTINIES phenotype.

As requested during final discussions, only a single phenotype was ratified for use in real-world data (the DESTINIES Phenotype; Fig. 1); this utilised risk bands to reconcile risk categorised and stratified approaches. Phenotype levels and their contents (relative to each level) are provided in descending levels of risk for severe COVID-19 outcomes. Band 1 levels are at major risk for severe COVID-19 outcomes, Band 2 levels are at moderate risk for severe COVID-19 outcomes and Band 3 levels are at minimal risk for severe COVID-19 outcomes. Italicised text denotes key exclusions; these were limited to those that are implementable in EMRs.

Drug-manged HIV was removed from the main body of the phenotype to reflect panel consensus that ‘*a patient would no longer be considered immunosuppressed if their HIV was drug-managed’*. This will be trialled as an internal reference group for comparative research—offering a point of relative immunocompetence and a better demographic and vaccine uptake match to the immunosuppressed than the general population control.

A dendrogram of the cluster analysis that served as an additional check on DESTINIES Phenotype structure is available in Supplementary Materials. This exercise indicated that there was sufficient similarity in vulnerability between phenotype levels to justify their continued grouping.

Final ratification data are also reported in Supplementary Materials. Panellists agreed with near unanimity that the DESTINIES Phenotype was an improvement from previous drafts (94%), represented study results accurately (91%) and warranted being trialled in real-world data (94%). Phenotype contents and cadence exceeded 75% consensus and panellists agreed on the exploratory value of comparing phenotype, reference and control group data for COVID-19 (85%) and other infection outcomes (RSV, pneumonia, seasonal influenza etc; 88%). With 12% and 24% disagreement, there were reservations around the use of risk bands and Drug-managed HIV as an internal reference group; validation studies will determinate their retention.

## Discussion

This study delivered the following outputs: a COVID-19 specific, internationally aligned and digitally practical phenotype for ‘adult immunosuppression’, current international consensus on immunosuppressed diagnoses and procedures at higher and lower risk for severe COVID-19 outcomes and agreement on a wide range of clinical statements. Subject to validation in real-world data, these hold significant promise for COVID surveillance and targeted patient care. Higher resolution intelligence can inform the prioritisation of COVID-specific medical resources (booster vaccinations, monoclonal antibodies, convalescent plasma, antivirals etc) and non-pharmaceutical interventions including shielding, improved ventilation and continued mask wearing in public spaces.

Those identified with consensus as at greatest risk for severe COVID-19 outcomes included Haemopoietic Transplant and Solid Organ Transplant recipients, those with Malignancies (Haematologic and Solid Tumour) and Primary Immunodeficiency, Aplastic anaemia, Renal autoimmune and Dialysis patients. Meanwhile, Drug-managed HIV, ME/CFS and the majority of Anatomical Barrier Defect and Wider Determinants of Immunosuppression candidates were deemed at lowest risk of severe COVID-19 within the immunosuppressed spectrum.

Despite multiple rounds of data collection and discussion, panellists failed to agree on either the appropriateness or COVID-19 risk profiles of certain candidates. Immune mediated inflammatory disorders were especially affected. There was also significant heterogeneity within common clinical categories. This served as a reminder for the complexity and idiosyncrasy of immunosuppression and the dynamic nature of their vulnerability: panellists continually caveated the influence of individual medication and demographic factors (e.g. age or comorbidity) when making their appraisals—caveats that were too nuanced to be codified in a CMR-compatible phenotype at this moment in time.

Consensus was achieved on the following clinical statements: that immunosuppression was poorly defined, that COVID-19 continued to pose a threat upon these patients, that said threat was not experienced equally across the patient spectrum and that COVID-19 care was currently insufficiently targeted to protect those most at risk. Agreement was also reached on the reversing of immunosuppressed status if a patient's cancer went into remission, if a patient's HIV was sufficiently drug-managed and if their immunosuppressive medications had been discontinued for over one year. Six months on an immunosuppressive regimen was considered sufficient time to create immunosuppression. Panellists noted exceptions to these areas of agreement, however—citing the long-term, even lifelong immunosuppression associated with rituximab and other B-Cell depleting therapies, and the non-reversal of immunosuppressed status if a cancer treated via haemopoietic transplantation achieved remission. Drug equivalence statements were rejected outright.

The heterogeneity of COVID-19 risk profiles within same clinical categories demonstrates the importance of constructing digital phenotypes that do not collapse higher risk diagnoses into a more moderate clinical category average. The DESTINIES phenotype achieves this, distinguishing metastasising cancer, unmanaged HIV and systemic immune-mediated inflammatory disease from their respective clinical categories. When implemented in real-world data, this will reduce the likelihood that sub-trends of clinical importance are erased.

The holistic interpretation of immunosuppression offered by the DESTINIES phenotype alerts policymakers, pharmaceutical companies and the wider public to the scale of vulnerability in the general population; viewing the immunosuppressed as a minor and thus low-priority group leads to underinvestment in their interests with severe health consequences.^24^ That said, panellists warned against prioritising all DESTINIES phenotype contents for enhanced care; this would not be economically tenable in any public health system.^25^ If validated in real-world data, risk-banding and dependency clinical statements should direct resources, with stock and shielding notices prioritised for those of highest risk. This will prevent the over-scoping that strained resource allocation during the COVID-19 pandemic.^26^ Maintaining broad and narrow definitions of immunosuppression in parallel is not unprecedented: this was the approach taken by Evans and colleagues (2023) to clarify COVID-19 outcomes in the immunosuppressed during the omicron era.^13^

This work is the first to address international inconsistencies in how immunosuppression is defined and risk assessed through the lens COVID-19. Consortium members acknowledged that this was an exceedingly difficult exercise with significant heterogeneity to navigate; achieving consensus in any domain was considered an accomplishment, bringing clarity to long-disagreed topics in the field. The calibre and global representativeness of panellists were also strengths of this work.

To the best of our knowledge, the DESTINIES Study is the first to recommend with clinical consensus that Drug-managed HIV be removed from characterisations of immunosuppression going forwards. This result is momentous given the longstanding vulnerability of this population and emphasises the importance of drug equity initiatives, including the World Health Organisation's 90:90:90 initiative.^27^ Panel ratification of Wider determinants of immunosuppression, Anatomical barrier defects and the demarcation between systemic and single site immune-mediated inflammatory conditions added scope and detail to existing definitions of immunosuppression.

Despite these achievements, the DESTINIES Study suffered from the following limitations. As highlighted within the study protocol,^10^ the Delphi method is not yet underpinned by clear, universalised guidelines. This creates considerable inconsistency in how these studies are conducted and enables highly iterative approaches to be taken. Such iteration could be criticised as unscientific: for example, adjustments made to study questions and statistical analysis based on panel feedback could be criticised. Providing panellists with interim data to inform said feedback may also have led to groupthink.^28^

Panellists were also disappointed by their inability to provide medicated vs unmedicated appraisals of candidates’ COVID-19 risk. While they appreciated the difficulties and poor accuracy associated with operationalising a medication-inclusive phenotype in real-world data, they questioned the comprehensiveness of the DESTINIES phenotype without them. The vagueness of certain candidate terms (e.g. Underlying aberrant immunity) also caused concern. These issues led to unresolved disputes over the appropriateness of several candidate terms. It is therefore our recommendation that a separate, but similar, online Delphi exercise be arranged to build international consensus on the types of medications and contextual factors that confer immunosuppression and their respective COVID-19 risk profiles. Subject to validation in real-world data, this could augment the present DESTINIES phenotype, providing parallel axes for COVID risk appraisal and surveillance stratification.

The highly generalised nature of clinical statements was also detrimental to consensus building. Many topics were highly context dependent. Panellists were especially resistant to identifying a standard medication dosage or time point where immunosuppression would no longer be conferred; this will be communicated to the authors of clinical guidelines that attempt this.^29^

Panel composition can also be criticised: a recruitment strategy that targeted public health agencies was not purposive enough to secure a fully representative panel. The absence or underrepresentation of certain continents were detrimental; LMIC contexts and considerations were only lightly addressed. Representativeness was also eroded by attrition between Round 2 and Final Discussion. These in-person discussion groups were affected by scheduling conflicts and often coincided with annual leave. It was an oversight to not remind those struggling to accommodate their in-person timeslots that written participation was available. Finally, despite approaching the patient champions of target entities, none agreed to participate in this work. This is now being addressed with the recruitment of a DESTINIES Patient Panel. Representing all immunosuppressive diagnoses and procedures cited in its final format, this Patient Panel will soon meet to critique the DESTINIES phenotype and catalogue patient concerns for its real-world implementation. It is essential that this phenotype does not cause undue distress amongst the patient population; higher risk bands may need to be contextualised, especially for their lowest risk constituents including Down Syndrome and Islet Transplantation patients. Refinements will be made if necessary.

As a collective, these limitations currently preclude the DESTINIES phenotype from supporting clinical decision-making; it must be validated within real-world dataflows before its surveillance can inform care. Likewise, without testing, this phenotype cannot be considered generalisable to other disease domains. The idiosyncrasies of COVID-19 infection, especially the protective value of certain immunosuppressed states and medications^30^ against critical outcomes, mean that the risk-hierarchy that surfaced in this exercise may not hold for other infection types.

To conclude, over the course of two online questionnaires, one discussion group and a final ratifying vote, this exercise has resolved several points of contention in the immunosuppressed literature and has produced a COVID-19 specific, internationally aligned and digitally practical means of identifying immunosuppressed patients in real-world data and risk-stratifying their infection outcomes. This DESTINIES phenotype will now be codified and tested within disease surveillance dataflows. In the spirit of open science, code lists will be published in public phenotype libraries to invite international collaboration and validation.

## Contributors

ML served as primary investigator for this work. Contributions included conceptualisation, data access, curation, formal analysis, funding acquisition, investigation, methodology, project administration, resources, software, data validation, visualisation, writing–original draft and writing–review and editing.

JOM, MJ, FDRH, SdeL, BT, IM and LL served as DESTINES Steering Group members. Contributions included conceptualisation, investigation, methodology, project administration, data access, data curation, data validation, manuscript review & editing.

IdG served as our DESTINIES Steering Group Patient Member. Contributions included conceptualisation, investigation, methodology, project administration, data access, data validation, manuscript review & editing.

HS, JI, MA, FR, WSL, RC, SS, IB, MU, DL, MH, CG, AA are DESTINIES Consortium members who provided substantiative enough feedback on the manuscript to warrant co-authorship. Contributions included investigation, review & editing.

## Data sharing statement

The DESTINIES Study protocol is publicly available and referenced within this work. Study data is provided in full in Supplementary Materials but can be requested from the corresponding author in spreadsheet format.

## Declaration of interests

The manuscript authors would like to declare the following interests:

SdeL has received funding for vaccine related work from AstraZeneca, GSK, Moderna, Pfizer, Sanofi, and Seqirus; has been funded for conference travel and has received a speaking fee from AstraZeneca and Moderna.

RC has received payment for research on behalf of Janssen, Celltrion, Roche, Sanofi, Abbvie, Galapagos, Fresenius Kapi, Viatris and UCB; has been funded for conference travel by Abbvie, Janssen and Nordic Pharma; has participated on an advisory board for Abbvie; holds other non-financial interests with Abbvie and Novartis (clinical trial participation).

SS has received institutional funding from GSK and Pfizer; has received consulting fees for AstraZeneca; has received payment for a research presentation by Pfizer; has received travel expenses from Pfizer; has been an unpaid advisory member of the McInnes Independent Advisory Group 2021–2023.

MU has received grants from the National Institute for Health and Care Research, the Norwegian Medical Research Council and the Australian National Health and Medical Research Council. He has received royalties from the University of Warwick and has participated on an advisory board at the National Institute of Health and Care Research. He holds stock with Clinvivo Ltd and has received clinical materials from Stryker PLC.

WSL currently holds a leadership position within the Joint Committee of Vaccination and Immunisation.

CG has received grants from Almirall and Boots UK as well as royalties as Wiley Editor of Rook's Textbook of Dermatology. He has received consulting fees from Johnson & Johnson, Inmagene, Evelo Bioscience, Bristol Meyers Squibb and Boehringer-Ingelheim. He has received payment for presentations at Abbvie, Lilly, Almirall, No. 7 Company, UCB, Bristol Meyers Squibb and Novartis as well as payment for expert testimony at Amryt Pharma and Johnson & Johnson. He participates on the advisory board for Artax and is treasurer for the International Societies of Investigative Dermatology. He holds stock in The Skin Diary.

DL has received a research grant via his institution from GSK and BMS; has received consultancy fees via institution from GSK; has provided educational lectures for Biotest, Takeda, AstraZeneca and Roche; has received support to attend a conference from Octapharma; serves within the Clinical Guidelines group of the British Society for Immunology.

IB has received a senior investigator award from the NIHR; has received consulting fees from AstraZeneca as their Chief Data Scientist Advisor (2019–2023); has served on the UK COVID-19 Testing Initiatives Evaluation Board.

BT has received Medical Research Future Fund Investigator Grant from the Australian Government, Seqirus funding and MSD funding paid to his institution; he serves on the Australasian Myeloma Research Consortium (unpaid) and Takeda, CSL-Behring and Moderna advisory boards paid to his institution; he serves on the Australian Technical Advisory Group on Immunisation.

IBM has received grants from Abbvie, Amgen, BMS, Causeway, Eli Lilly, Gilead, Janssen, Novartis, Pfizer, Sanofi Regeneron, UCB Pharma, Evelo, Compugen and AstraZeneca; he has received consulting fees from Abbvie, Amgen, BMS, Causeway Therapeutics, Cabaletta, Eli Lilly, Gilead, Janssen, Novartis, Pfizer, Sanofi Regeneron, UCB Pharma, Evelo, Compugen and AstraZeneca; he serves as a board member for NHS Greater Glasgow & Clyde Health Board, a Trustee at vs Arthritis and Vice Principal and Head of College at the University of Glasgow; he holds stock options with Evelo, Compugen and Cabaletta.

ML is funded by an MRC iCASE Award between the UK Medical Research Council and EMIS Health.

## References

[1] Cohen J., Warrell D.A., Firth J.D., Cox T.M. (2010). Oxford Textbook of Medicine.

[2] Harpaz R., Dahl R.M., Dooling K.L. (2016). Prevalence of immunosuppression among US adults, 2013. JAMA.

[3] Martinson M.L., Lapham J. (2024). Prevalence of immunosuppression among US adults. JAMA.

[4] Shoham S., Batista C., Amor Y.B. (2023). Vaccines and therapeutics for immunocompromised patients with COVID-19. eClinicalMedicine.

[5] Belsky J.A., Tullius B.P., Lamb M.G., Sayegh R., Stanek J.R., Auletta J.J. (2021). COVID-19 in immunocompromised patients: a systematic review of cancer, hematopoietic cell and Solid organ transplant patients. J Infect.

[6] Mercer T.R., Salit M. (2021). Testing at scale during the COVID-19 pandemic. Nat Rev Genet.

[7] Furstenau L.B., Rabaioli B., Sott M.K. (2021). A bibliometric network analysis of coronavirus during the first eight months of COVID-19 in 2020. Int J Environ Res Public Health.

[8] Tangye S.G., COVID Human Genetic Effort consortium (2022). Impact of SARS-CoV-2 infection and COVID-19 on patients with inborn errors of immunity. J Allergy Clin Immunol.

[9] Whitaker H.J., Tsang R.S., Byford R. (2022). Pfizer-BioNTech and Oxford AstraZeneca COVID-19 vaccine effectiveness and immune response among individuals in clinical risk groups. J Infect.

[10] Leston M., Ordóñez-Mena J., Joy M. (2024). Defining and risk-stratifying immunosuppression (the DESTINIES Study): protocol for an electronic Delphi study. JMIR Res Protoc.

[11] Diamond I.R., Grant R.C., Feldman B.M. (2014). Defining consensus: a systematic review recommends methodologic criteria for reporting of Delphi studies. J Clin Epidemiol.

[12] Leston M., Elson W., Ordóñez-Mena J.M. (2024). Disparities in COVID-19 mortality amongst the immunosuppressed: a systematic review and meta-analysis for enhanced disease surveillance. J Infect.

[13] Evans R.A., Dube S., Lu Y. (2023). Impact of COVID-19 on immunocompromised populations during the omicron era: insights from the observational population-based INFORM study. Lancet Reg Health.

[14] Barnes E., Goodyear C.S., Willicombe M. (2023). SARS-CoV-2-Specific immune responses and clinical outcomes after COVID-19 vaccination in patients with immune-suppressive disease. Nat Med.

[15] Teh B.W., Mikulska M., Averbuch D. (2024). Consensus position statement on advancing the standardised reporting of infection events in immunocompromised patients. Lancet Infect Dis.

[16] National cancer institute. https://www.cancer.gov/Publications/Dictionaries/Cancer-Terms/Def/Immunosuppressed.

[17] Abu-Raya B., Michalski C., Sadarangani M., Lavoie P.M. (2020). Maternal immunological adaptation during normal pregnancy. Front Immunol.

[18] Morey J.N., Boggero I.A., Scott A.B., Segerstrom S.C. (2015). Current directions in stress and human immune function. Curr Opin Psychol.

[19] Garbarino S., Lanteri P., Bragazzi N.L., Magnavita N., Scoditti E. (2021). Role of sleep deprivation in immune-related disease risk and outcomes. Commun Biol.

[20] Bell S.K., Delbanco T., Elmore J.G. (2020). Frequency and types of patient-reported errors in electronic health record ambulatory care notes. JAMA Netw Open.

[21] Säfholm S., Bondesson Å., Modig S. (2019). Medication errors in primary health care records; a cross-sectional study in southern Sweden. BMC Fam Pract.

[22] Mirhashemi S.H., Ramezanghorbani N., Asadi F., Rangraz M.H. (2020). Auditing the accuracy of medical diagnostic coding based on international classification of diseases, tenth revision. Iran Red Crescent Med J.

[23] Public Health England (2016).

[24] Trøseid M., Hentzien M., Ader F. (2022). Immunocompromised patients have been neglected in COVID-19 trials: a call for action. Clin Microbiol Infect.

[25] Ferguson C. (2007).

[26] Herrick C. (2022). “We thank you for your sacrifice”: clinical vulnerability, shielding and biosociality in the UK's Covid-19 response. Biosocieties.

[27] UNAIDS (2016). http://www.unaids.org/en/resources/909090.

[28] Janis, Irving L. (1972).

[29] (2020). COVID-19: The Green Book, Chapter 14a.

[30] Schoot T.S., Kerckhoffs A.P.M., Hilbrands L.B., van Marum R.J. (2020). Immunosuppressive drugs and COVID-19: a review. Front Pharmacol.

