# Supplementary material for: The DESTINIES Study: an online Delphi study to build international consensus on the medical conditions and procedures that confer immunosuppression and their respective COVID-19 risk profiles
Source: eClinicalMedicine. 2025 May 5;83:103239. doi: 10.1016/j.eclinm.2025.103239 (PMC12124667; doi:10.1016/j.eclinm.2025.103239)
Supplement: Supplementary Materials [file mmc1.docx]

**Supplementary Materials**

1. **Schematic of DESTINIES Study Timeline**


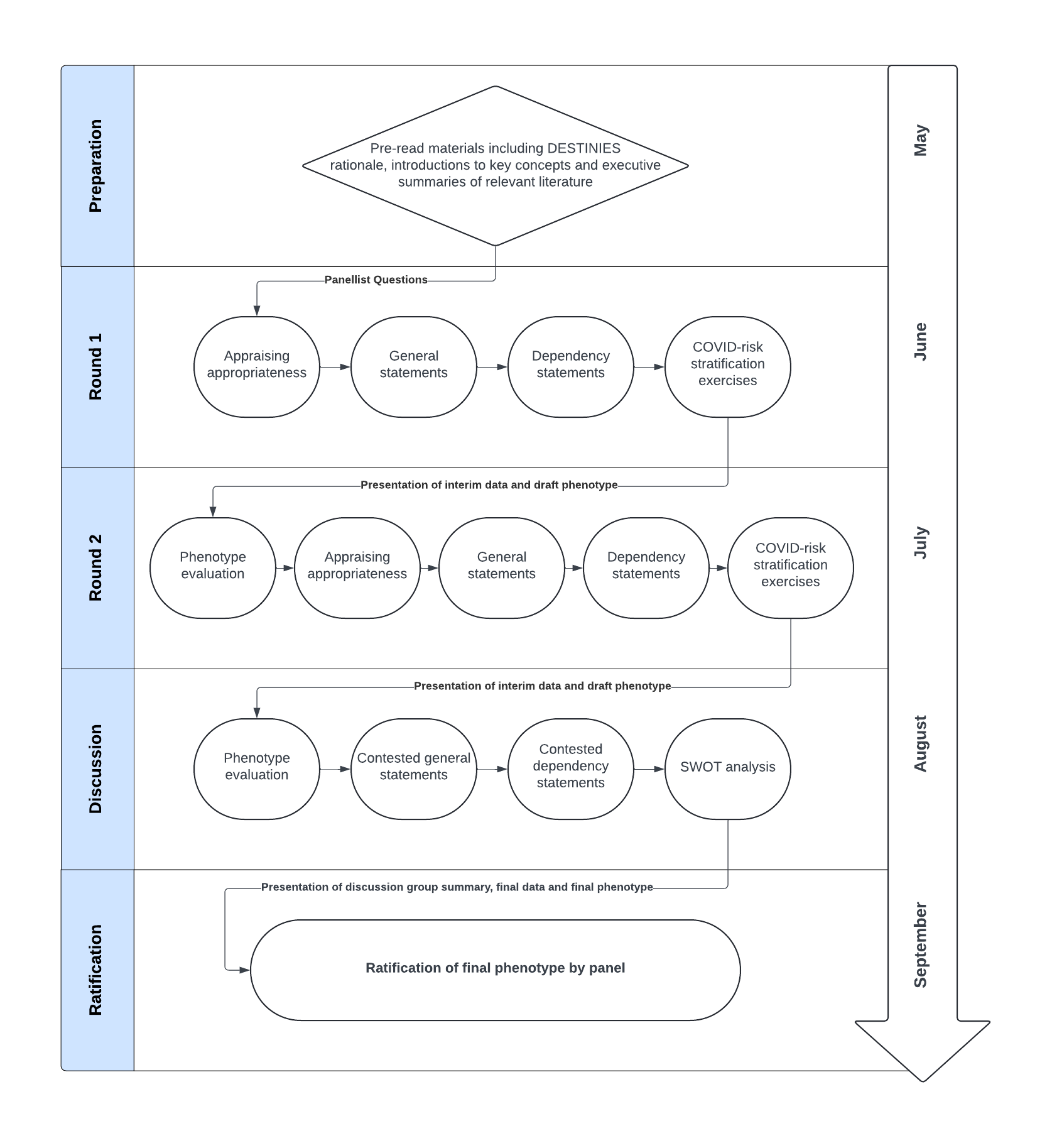


1. **List of Public Health Institutions Approached for Panel Recruitment**

- World Health Organization (WHO) Global Advisory Committee on Vaccine Safety (GACVS)
- Coalition for Epidemic Preparedness Innovations (CEPI) Scientific Advisory Committee (SAC)
- The Global Immunocompromised Health Coalition (GHIC)
- The European Alliance of Associations for Rheumatology (EULAR)
- European Medicines Agency (EMA) Vaccine Working Party
- UK Joint Committee on Vaccination and Immunisation (JCVI)
- UK COVID-19 Neutralising Monoclonal Antibodies (nMABs) and Antivirals Access Independent Advisory Group (Van Tam Advisory Group)
- UK Scientific Advisory Group for Emergencies (SAGE)
- USA Centers for Disease Control and Prevention (CDC) Advisory Committee on Immunization Practices (ACIP)
- USA Food and Drug Administration (FDA) Vaccine Advisory Panel
- University of Oxford Nuffield Department of Primary Care Health Sciences (NDPCHS)
- University of Oxford Nuffield Department of Medicine (NDM)

1. **Pre-read materials & Consent Form**


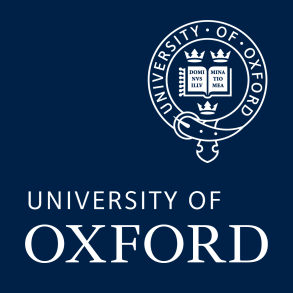
Meredith Leston, Primary Investigator

Radcliffe Primary Care Building

Radcliffe Observatory Quarter

Oxford

OX2 6GG

[meredith.leston@phc.ox.ac.uk](mailto:meredith.leston@phc.ox.ac.uk)

+447896980320

**PANNELLIST INFORMATION SHEET**

The Inaugural UK e**DE**lphi **ST**udy to Def**IN**e and Risk-Stratify **I**mmunosuppr**ES**sion (**the DESTINIES study**):

We are delighted that you will be participating in this eDelphi study to address global inconsistencies in how immunosuppression is characterised and subdivided as a clinical risk group. It is our intention that this work will deliver an internationally aligned, clinically meaningful and medical record compatible means of identifying immunosuppressed patients and subdividing their health outcomes by reported vulnerability to severe COVID-19 infection.

**Why have I been invited?**

You have been asked to participate in this study in recognition of your track record in immunology, vaccinology and/or clinical risk prioritisation or your affiliation with the decision-making bodies listed:

- World Health Organisation (WHO) Global Advisory Committee on Vaccine Safety (GACVS)
- Coalition for Epidemic Preparedness Innovations (CEPI) Scientific Advisory Committee
- The Global Immunocompromised Health Coalition (GHIC)
- The European Alliance of Associations for Rheumatology (EULAR)
- European Medicines Agency’s (EMA) Vaccines Working Party (VWP)
- Joint Committee on Vaccination and Immunisation (JCVI)
- COVID-19 Neutralising Monoclonal Antibodies (nMABs) and Antivirals Access Independent Advisory Group
- UK Scientific Advisory Group for Emergencies (SAGE)
- Independent Scientific Advisory Group for Emergencies (Indie_SAGE)
- Centers for Disease Control and Prevention (CDC) Advisory Committee on Immunization Practices (ACIP)
- Food and Drug Administration (FDA) Vaccine Advisory Panel
- The Nuffield Department of Primary Care Health Sciences
- The Nuffield Department of Medicine

**How do I take part?**

We kindly request that our panellists read all the materials included in this document before the first eDelphi round begins. These resources have been specially compiled to provide panellists with background information on the study, digital phenotyping, differential vulnerability to COVID-19 in the immunosuppressed and the strengths of consensus exercises.

For the two questionnaire rounds of this eDelphi study, you will be emailed a personal link to Google Forms to submit your answers (approx. 15 minutes each). You will be given a period of two weeks to do so. You will also be allocated a Panellist ID number for your anonymity and study management purposes. However, should you have any discomfort with completing this Google Form, you also have the option to request a paper-based version of this survey to complete and return to researchers. Please contact the Primary Investigator at the details provided as soon as possible if you would like to participate by this route.

In the two weeks between questionnaire rounds, you will be provided with a summary of the data collected from the previous round, including distributions of how the panel has scored each questionnaire item and how your own score compares. You will also be provided with two draft phenotypes of immunosuppression in adults. Both will COVID risk-stratify immunosuppressed conditions based on panellist answers from the previous round; the first will do so as a higher vs lower risk binary, the second in multiple levels. You will be asked to evaluate the accuracy of these drafts in the next round.

For the final discussion group portion of this study (hosted virtually via Microsoft Teams), all panellists will be presented with three, time-zone adjusted options to choose from. The option that receives the most votes will be taken forward; panellists unable to attend this timeslot will be provided with an additional Google Form to address the questions that will be discussed or a paper-based alternative. To retain panellist anonymity, all those joining these online discussion groups will be instructed to keep their cameras switched off.

**What are the key study dates?**

- 24th May - *first round begins*
- 7th June - *end of first round*
- 14th June - *dissemination of interim results and draft phenotypes*
- 21st June - *second round begins*
- 5th July - *end of second round*
- 12th July - *dissemination of interim results, draft phenotypes and booking poll for final discussion groups*
- w/c 22nd July - *3x final discussion groups (panel divided by time zone) and dissemination of final google form for those unable to participate.*
- 26th July - *end of study.*

**What are the possible benefits of taking part?**

Your participation will provide the evidence base needed to surface an internationally aligned and computerised medical record compatible definition for immunosuppression in adults and a clinically meaningful form of subdivision. Based on panellists’ instruction, digital phenotypes for a COVID Risk Hierarchy and a Higher vs Lower Risk Binary will be constructed and implemented into national disease surveillance flows. These digital phenotypes will be published on dedicated online libraries (BioPortal/ PhenoFlow), with due attribution given to all consenting panellists. Likewise, panellists that are willing to be named will be listed as co-authors on all experimental outputs; those that prefer to remain anonymous will have their contribution acknowledged under the umbrella term ‘The DESTINIES Consortium’.

**Will my taking part in the study be kept confidential?**

The use of Panellist ID numbers throughout this study ensures that panellists can remain anonymous while giving their professional opinion. Only researchers will have access to identifying information during data collection. However, panellists that wish to be named in experimental outputs and publications will be given due attribution and co-author status. Those that do not will be acknowledged under ‘The DESTINIES Consortium’ umbrella, as mentioned.

All study data will be kept safely on a secure computer network; only those who have passed the necessary security checks will have access to this research environment. Responsible members of the University of Oxford may be given access to your answers for monitoring and/or audit of the study to ensure that the research is complying with all regulations. However, again, they will not have access to any personal information about you and will not be able to identify you at any point.

**Will I be reimbursed for taking part?**

You will not be reimbursed for participating.

**What will happen to my data?**

Your data will be retained for a period of 18 months, after which it will be deleted from the secure server and removed from Google Forms.

**What will happen if I don't want to carry on with the study?**

Participation in this research is completely voluntary. Should you wish, you do not have to give any reason for withdrawing from this study. However, any answers you had given up until this point will be used in analysis, unless you explicitly ask for this not to occur.

**What if there is a problem?**

If you have a concern about any aspect of this study, please speak with research team at the details provided. They will do their best to answer your questions.

If you wish to complain about any aspect of the way in which you have been approached or treated, or how your information is handled during the course of this study, you should contact Meredith Leston ([meredith.leston@phc.ox.ac.uk](mailto:meredith.leston@phc.ox.ac.uk); +447896980320) or you may contact the Research Governance, Ethics & Assurance (RGEA) Office on 01865 616480 or, for the RGEA Team lead, email [rgea.complaints@admin.ox.ac.uk](mailto:rgea.complaints@admin.ox.ac.uk)

**How have patients and the public been involved in this study?**

This work has the oversight of the European Alliance of Associations for Rheumatology (EULAR) People with Arthritis/Rheumatism across Europe (PARE). A patient research expert from this organisation sits on the steering committee of this study; her role is to ensure that he questions you are being presented with are of maximal value to patients, clinicians and their caretakers and that findings are disseminated to these groups in an accessible manner.

**Who is organising and funding the study?**

The study is organised by Ms Meredith Leston from the Nuffield Department of Primary Care Health Sciences at the University of Oxford. This research is funded by EMIS Health and the Medical Research Council.

*Thank you for taking part in this research.*

**Study Background:**

The term immunosuppression is generally accepted as referring to those who, either due to a specific diagnosis, procedure, or course of medication, mount an insufficient immune response to infection and onward disease. This term is sometimes used synonymously with immunodeficient or immunocompromised, sometimes as a standalone.

Indeed, there remains considerable disagreement over the exact conditions that confer immunosuppressed status and how this patient spectrum might be meaningfully subdivided or risk categorised. These inconsistencies are most apparent when comparing international clinical guidelines. For example, the definition offered for immunosuppression by *Immunisation against infectious disease: The Green Book* in the United Kingdom is markedly different in scope and conditionality than equivalent national handbooks. Even very subtle differences in definitions can have significant impacts on population sizing: depending on criteria used, the immunosuppressed patient spectrum can constitute anywhere between 2% to 10% of the general population. Inconsistency here fragments the research, disease surveillance and health policy landscape as findings from discordant populations cannot be easily compiled, compared, or actioned. This confusion undermines stated ambitions for targeted patient care.

The COVID-19 pandemic, an ongoing event from the perspective of the clinically vulnerable, typifies these issues and provides a unique opportunity for improvement. Despite collective motivation to protect immunosuppressed patients, conflicting clinical definitions have created international rifts in how these patients have been monitored and managed. That said, thanks to unprecedented levels of collaboration and community testing, there has been a proliferation in literature reporting immunosuppressed health outcomes. Indeed, COVID data provides a much-needed common denominator for establishing differential vulnerability in this diverse clinical risk group. Even rare and complex immunosuppressed conditions have been centred in COVID infection and vaccine research. This is a departure from the previous convention of aggregating immunosuppressed outcomes or selectively reporting those from dominant subgroups.

The DESTINIES Study seeks to respond to these developments, convening over 70 world-leading clinical, research or policy experts in immunology and/or clinical risk prioritisation to define immunosuppression in adults and respond to the current evidence base for differential vulnerability to COVID-19 amongst these patients. This study will appraise all conditions currently associated with immunosuppression, identifying key omissions and redundancies in national definitions to produce an internationally aligned alternative. A series of consensus statements and COVID risk ranking exercises will inform how this can be risk stratified going forwards. A final discussion group will refine these outputs before they are codified into a digital phenotype for onward use in real world surveillance dataflows.

Although COVID-specific, the international definition and risk-stratification that will emerge over the course of this study have significant prospective value for clinical decision-making across all diseases that disproportionately affect the immunosuppressed.

**Executive Summary I:** [***‘Disparities in COVID-19 mortality amongst the immunosuppressed: A systematic review and meta-analysis for enhanced disease surveillance’***](https://www.journalofinfection.com/article/S0163-4453(24)00028-8/fulltext)

This systematic review and meta-analysis assessed whether excess COVID-19 mortality compared to the immunocompetent could successfully subdivide the immunosuppressed patient spectrum. To do so, authors converted all immunosuppressed terminology included within UK *Immunisation against infectious disease: The Green Book* into search terms compatible with OVID (EMBASE, MEDLINE, Transplant Library and Global Health), PubMed and Google Scholar databases. This characterisation of the immunosuppressed was chosen because of its influence over vaccine prioritisation in the UK and, as a medication- and condition-inclusive definition, the expansive but differentiable entry point it provided into the immunosuppressed literature. This syntax was combined with COVID-19 and mortality terms before being entered into the databases specified.

Literature was screened in two phases of stringency: the first to establish the feasibility of meta-analysis and the second to extract the necessary information to carry it out. Inclusion and exclusion criteria for both phases are specified in Textbox 1.

**Textbox 1: Inclusion and Exclusion Criteria**

Phase 1:

Inclusion criteria were as follows:

- Study investigated the impact of COVID-19 on at least one immunosuppressed subgroup
- (as defined by the UK guidance); pre-prints were permissible
- COVID-19 associated mortality data was provided in some form on an immunosuppressed
- subgroup basis.

Exclusion criteria were as follows:

- Study did not investigate the impact of COVID-19 on at least one immunosuppressed
- subgroup (as defined by UK guidance); studies that only assessed this at the aggregate
- level of "the immunosuppressed' were also ineligible
- Mortality data was not provided in any form
- Study only presented indirect COVID-19 mortality data - that created by disruptions to
- diagnosis or treatment seen over the course of the pandemic
- Sample size for either immunosuppressed or immunocompetent groups were less than 50
- people
- Multiple or overlapping risk groups were assessed (e.g. pregnant/diabetic
- immunosuppressed patients)
- Immunosuppressants were studied as a prospective treatment for COVID-19, not as a risk
- factor for adverse outcomes
- Study included paediatric patients (under 18 years)
- Study only presented case series (n<50) or single-centre data.

Phase 2:

As per Phase 1, and

Additional inclusion criteria:

- Mortality data was sufficiently detailed for meta-analysis: studies reported mortality for
- both the relevant immunosuppressed subgroup and their immunocompetent control either
- through a combination of sample size and proportion death or via comparative effect
- measures and their respective confidence intervals.

Additional exclusion criteria:

- Study was published in 2023
- Study used established population-level mortality statistics in lieu of a formal control
- group
- Study only reported compound critical outcomes (e.g. ICU and death)
- Study only reported all-cause mortality
- Study presented a combined estimate of excess mortality via meta-analysis
- Study did not present its own mortality data but summarised other authors' findings.

Figure 1 illustrates the entire screening process. As seen, source material was forward and backwards citation tracked to maximise catchment; papers published in and after 2023 were excluded to minimise the influence of novel therapeutics and vaccines. Once complete, screening returned 99 unique studies that offered data on both immunosuppressed and immunocompetent control mortality outcomes via effect size or absolute case and mortality figures. 18 studies offered mortality data for multiple types of immunosuppressed patients.

**Figure 1: Study selection flow diagram**


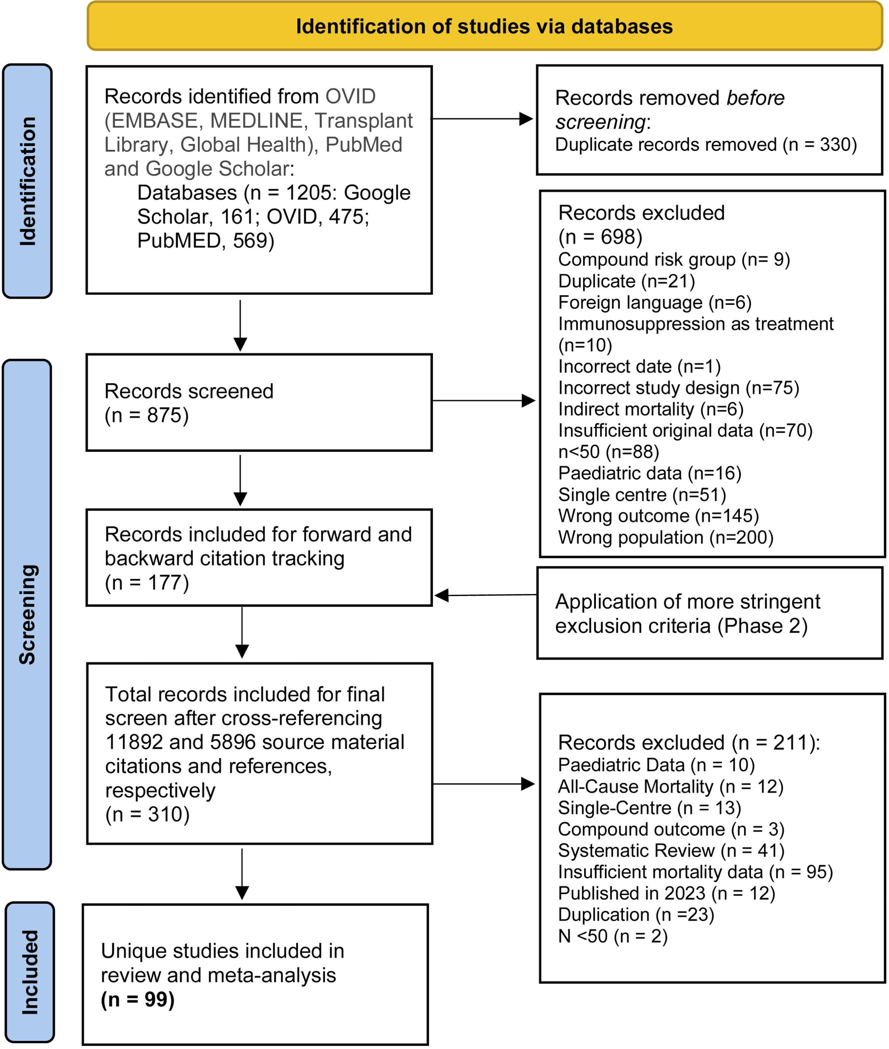


Study screening, extraction and bias assessment were performed blinded and independently by two researchers; conflicts were resolved with the oversight of a third. Information extracted included: title, author(s), publication year, income level (classified by World Bank as 'higher' for high-income countries and 'lower' for low, lower-middle, and upper-middle income), immunosuppression type, case type (All Cases or Hospitalised Cases), time to mortality as well as average age, sample size, number of deaths and/or comparative effect measure type, value, confidence interval (CI) and p-value for both immunosuppressed and immunocompetent cohorts. Odds ratios were imputed where necessary. Overall, data from 1,542,097 immunosuppressed patients and 56,248,181 immunocompetent comparators were analysed.

Odds Ratios (OR) and 95% confidence intervals (CI) of COVID-19 mortality were meta-analysed by immunosuppressed category and subcategory, specified in Table 1 below. The weighted average of these odds ratios denoted the pooled intervention effect (Effect size) of the specific form of immunosuppression over COVID-19 mortality. Subgroup analyses differentiated these estimates by effect measure, country income, case type, level of adjustment, use of matching and publication year. Meta regressions for average participant age were also run to establish whether excess mortality risk amongst immunosuppressed COVID patients was constant across the lifespan.

**Table 1: Immunosuppressed categories and their constituent subcategories**


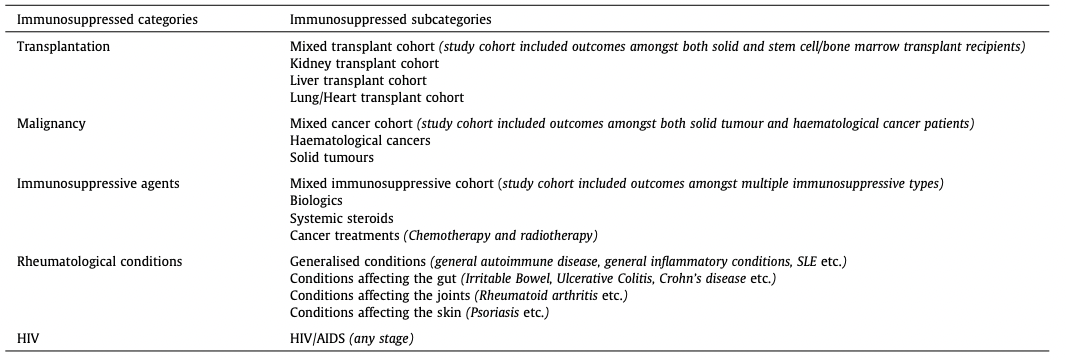


Figure 2 presents the results of meta-analysis run at the immunosuppressed category level as a summary forest plot. Our findings suggested that compared to the immunocompetent, transplantation recipients, malignancy patients and recipients of immunosuppressive agents had greater COVID-19 mortality risk than rheumatological or HIV patients. Within-category heterogeneity was substantial.

**Figure 2: Excess COVID-19 associated mortality by immunosuppressed category:**


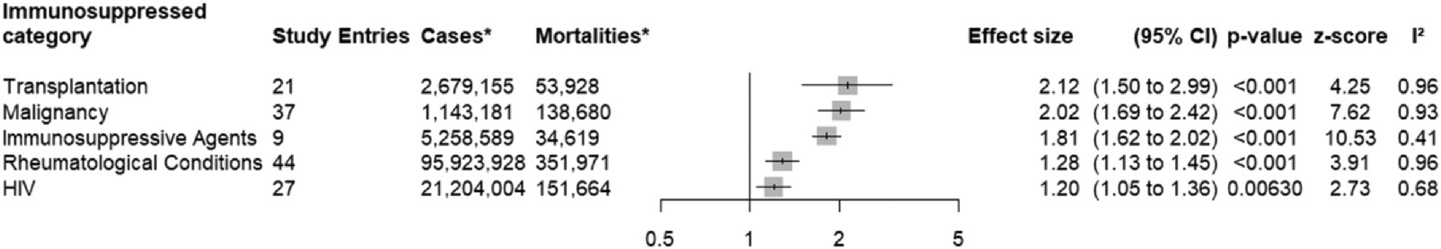


*The column labelled as Cases indicates the total number of participants, immunosuppressed and immunocompetent combined. The column labelled as Mortalities indicates the total number of deaths registered in participants, immunosuppressed and immunocompetent combined. Instances of multiple counts were not removed.

Figure 3 presents the results of meta-analysis run at the immunosuppressed subcategory level as a summary forest plot. This demonstrates the influence of certain subcategories over category estimates of excess mortality.

**Figure 3: Excess COVID-19 associated mortality by immunosuppressed subcategory:**


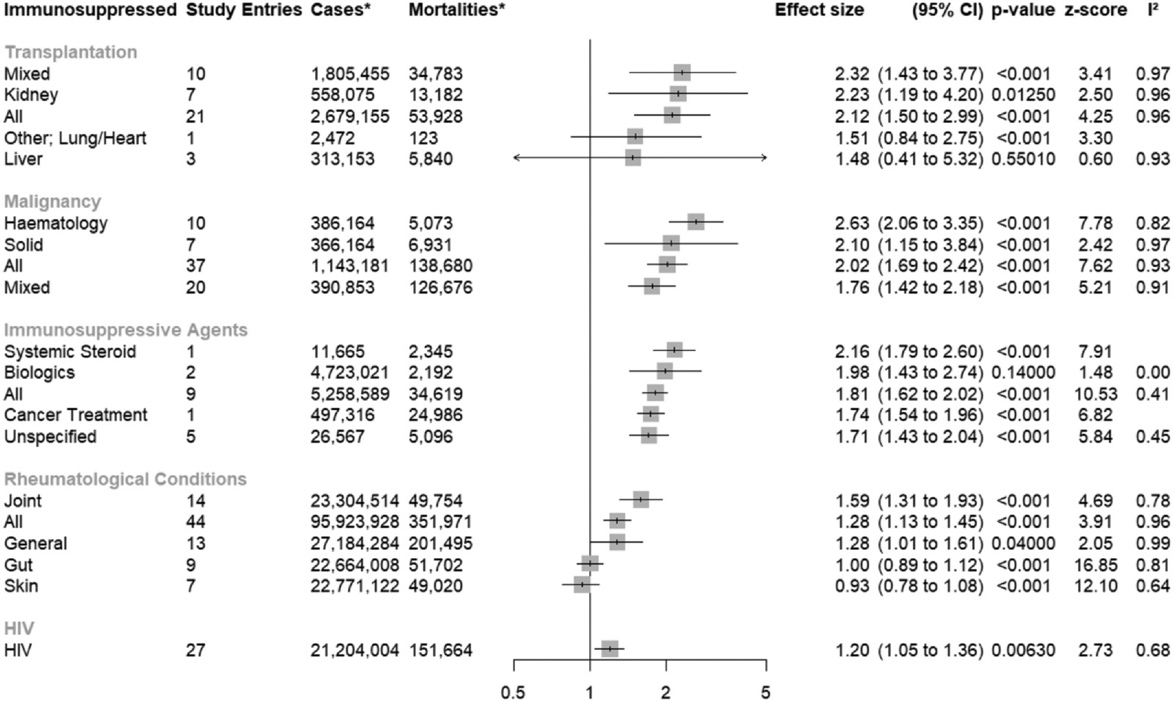


*The column labelled as Cases indicates the total number of participants, immunosuppressed and immunocompetent combined. The column labelled as Mortalities indicates the total number of deaths registered in participants, immunosuppressed and immunocompetent combined. Instances of multiple counts were not removed.

The elevated vulnerability of haematological malignancy patients, systemic steroid recipients and joint-related rheumatological conditions likely pulled category estimates upwards. Meanwhile, the resilience of skin-related rheumatological conditions as well as the single study estimates of cancer treatments and liver and heart/ other transplantations likely pulled category estimates downwards. Figure 4 highlights these relationships by presenting category and subcategory data in descending order of effect size.

**Figure 4: Excess COVID-19 associated mortality by immunosuppressed category and subcategory in descending order of effect size.**


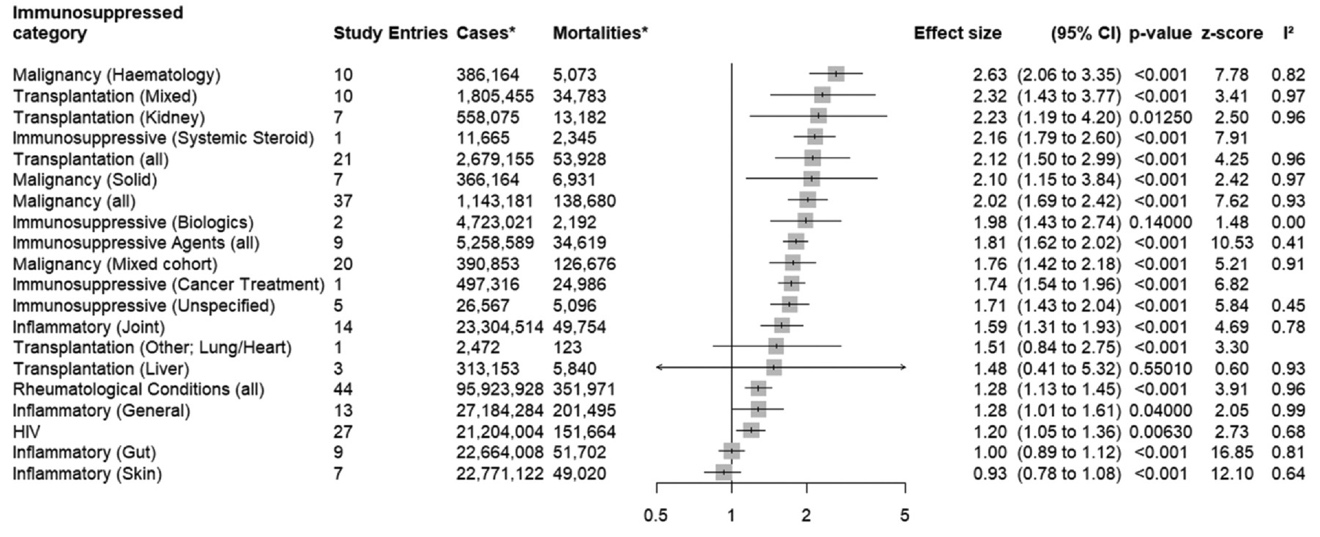


*The column labelled as Cases indicates the total number of participants, immunosuppressed and immunocompetent combined. The column labelled as Mortalities indicates the total number of deaths registered in participants, immunosuppressed and immunocompetent combined. Instances of multiple counts were not removed.

As illustrated in Table 2, subgroup analyses revealed that case type, study income level, level of adjustment (age, sex, comorbidity at a minimum), use of matching and publication year affected estimates for excess COVID-19 mortality compared to the immunocompetent.

**Table 2: Subgroup analyses of excess COVID-19 mortality by immunosuppressed category**


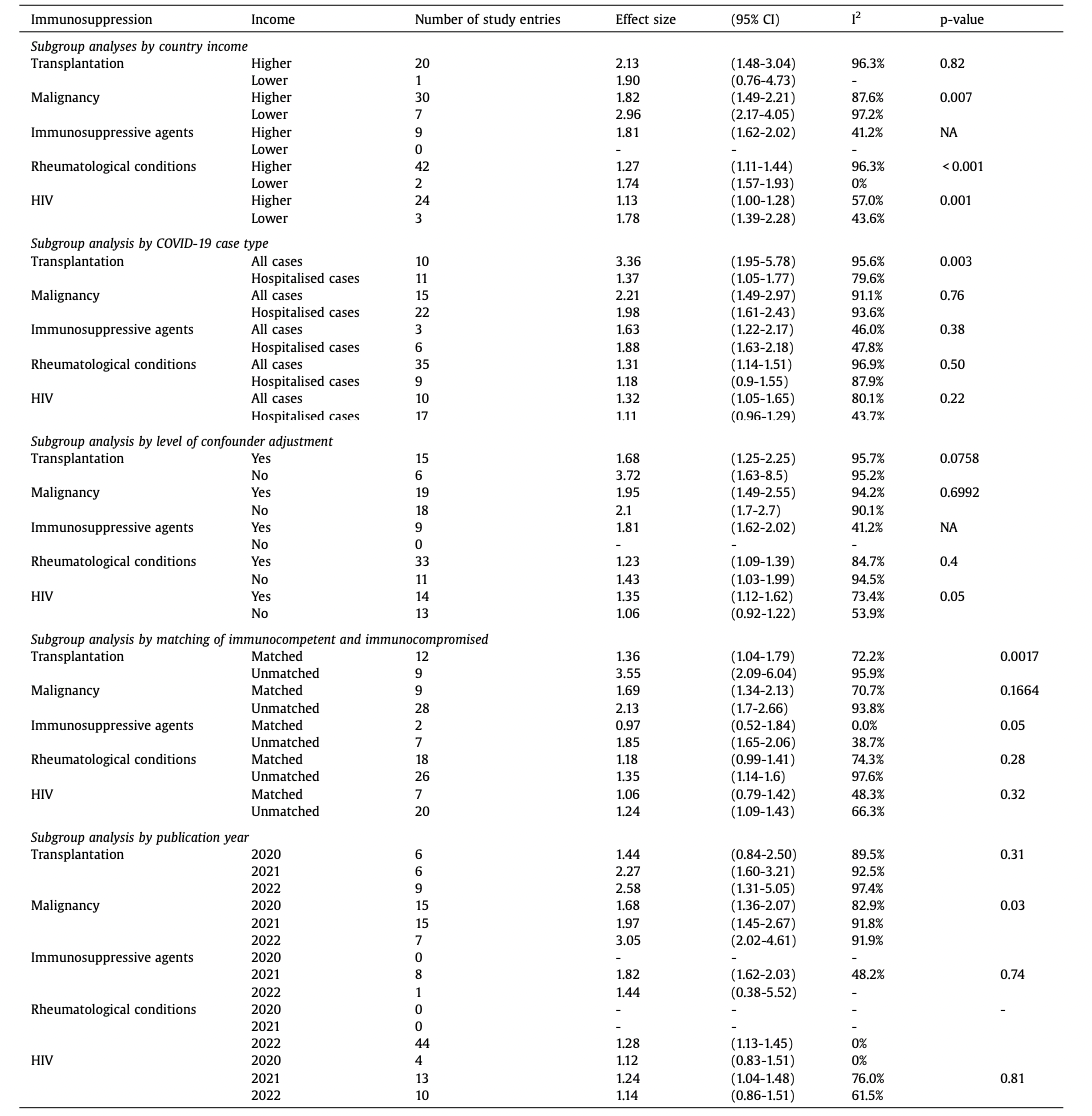


Effect sizes are generally lower in studies that only included hospitalised patients, were conducted in higher income countries, and analysed matched or adjusted data.

Encouragingly, all findings presented here are in broad agreement with preliminary reports of differential COVID-19 outcomes made by the Investigation oFcOvid-19 Risk amongst iMmunocompromised populations (INFORM) study – elaborated upon within these pre-read materials. To the best of authors’ knowledge, however, this work is the first to appraise the immunosuppressed COVID-19 literature comprehensively and comparatively. It has generated insights that are highly informative for disease surveillance. Indeed, the risk gradient in excess COVID-19 mortality observed may prove beneficial for allocating scarce medical resources such as booster vaccine doses, antivirals, monoclonal antibodies, and convalescent plasma. The priority now is to translate these findings into a definitive COVID-19 risk hierarchy or binary via clinical consensus building.

**Executive Summary II: *‘[Impact of COVID-19 on immunocompromised populations during the Omicron era: insights from the observational](https://www.thelancet.com/journals/lanepe/article/PIIS2666-7762(23)00166-7/fulltext#:~:text=Overall%2C%20immunocompromised%20individuals%20accounted%20for,COVID%2D19%20deaths%20in%202022.)***

***[population-based INFORM study’](https://www.thelancet.com/journals/lanepe/article/PIIS2666-7762(23)00166-7/fulltext#:~:text=Overall%2C%20immunocompromised%20individuals%20accounted%20for,COVID%2D19%20deaths%20in%202022.)***

Even as COVID-19 transitions from a pandemic to an endemic health threat, this disease continues to pose a significant risk to immunocompromised patients. Indeed, recent study of a large US health claims database estimated that as much as 23.5% of immunocompromised patients experienced a COVID-19 related hospitalisation. The protection offered against severe illness by COVID-19 vaccination is inconsistent in this diverse patient group; additional preventative methods, including ongoing isolation or social distancing, may be required amongst some.

The INFORM study was developed to characterise the continued risk of severe COVID-19 outcomes amongst the immunosuppressed and to identify the specific subgroups that may benefit from enhanced medical support. Using routinely collected healthcare record data from a random sample of 25% of the total English population over the age of 12 years, this work collected information on COVID vaccine status, COVID-related prescribing, and COVID-associated health outcomes (hospitalisation, ICU admission and mortality) for the entirety of 2022. Baseline demographic information (age, sex, number of non-immunocompromising comorbidities associated with increased risk of severe COVID-19 outcomes, BMI and IMD) was also described for all populations assessed. Sources included General Practice Extraction Service Data for Pandemic Planning and Research (GDPPR), COVID-19 Second Generation Surveillance System (SGSS), COVID-19 vaccination status data, Hospital Episode Statistics (HES), NHS Business Service Authority (BSA) dispensing data, Office of National Statistics (ONS) data and Personal Demographics Service (PDS) data.

The immunocompromised cohort was classified into the categories listed.

1. Broadly defined immunocompromised, including any individual diagnosed or receiving one or more of the following:
   1. Primary immunodeficiency ≤5 years prior to 1 January 2022.
      Secondary immunodeficiency
   2. Immunosuppressive therapy [ies] ≤12 months prior to 1 January 2022. High-dose or long-term moderate dose corticosteroids, chronic immune-mediated inflammatory diseases who received high-dose corticosteroids (≥20 mg prednisolone per day) for >10 days in the month prior to 1 January 2022; or chronic immune- mediated inflammatory disease who received long- term moderate dose corticosteroids (10 mg prednisolone per day for >4weeks) ≤3 months prior to 1 January 2022; or high-dose steroids (>40 mg prednisolone per day for >1 week) for any reason in the month prior to 1 January 2022.
   3. End-stage kidney disease (ESKD) ESKD ≤5 years prior to 1 January 2022.
   4. Solid or islet organ transplant (not including corneal transplants) prior to 1 January 2022.
   5. Stem cell transplants: haematopoietic stem cell transplants 2 years prior to 1 January 2022.
   6. Solid tumour: solid tumour [s] ≤5 years prior to 1 January 2022.
   7. Haematological malignancy: haematological malignancy [ies] ≤5 years prior to 1 January 2022.
   8. Advanced or untreated human immunodeficiency virus (HIV): clinical manifestations of symptomatic HIV (i.e., diagnostic code for HIV/acquired immunodeficiency syndrome (AIDS) or AIDS-defining conditions) ≤12 months prior to 1 January 2022. As CD4 counts were not available in the data, only patients with AIDS-defining illness were captured.
2. Stringently defined immunocompromised, a subset of the above including any individual diagnosed or receiving one or more of the following:
   1. Moderate to severe primary immunodeficiency: common variable immunodeficiency disease, severe combined immunodeficiency, DiGeorge syndrome, or Wiskott-Aldrich syndrome ≤5 years prior to 1 January 2022.
   2. Active treatment with non-corticosteroid immuno- suppressive or immunomodulatory therapy: treatment with alkylating agents, antimetabolites, transplant-related immunosuppressive drugs, cancer chemotherapeutic agents classified as severely immunosuppressive, TNF blockers, and other bio- logic agents that are immunosuppressive or immunomodulatory (e.g., B-cell depleting agents) ≤12 months prior to 1 January 2022.
   3. High-dose corticosteroids: treatment with high-dose corticosteroids steroids (i.e., ≥20 mg prednisone or equivalent per day when administered ≥2 weeks) in the month prior to 1 January 2022.
   4. Solid organ transplant: solid organ transplant (excluding corneal transplants) or islet transplant ≤2 years prior to 1 January 2022.
   5. Stem cell transplant: haematopoietic stem cell transplant ≤2 years prior to 1 January 2022.
   6. Solid or haematologic malignancy on active treat- ment: solid tumour [s] or haematologic malignancies on treatment ≤6 months prior to 1 January 2022.
   7. Chronic lymphocytic leukaemia, non-Hodgkin’s lymphoma, multiple myeloma, acute leukaemia: chronic lymphocytic leukaemia, non-Hodgkin’s lymphoma, multiple myeloma, or acute leukaemia ≤2 years prior to 1 January 2022.
   8. Advanced or untreated HIV: same definition as above.

Immunocompromised groups were not mutually exclusive; individuals could contribute date to any group they were eligible. Additional subgroups were also introduced to provide a more granular view of COVID-19 burden in groups known to be at particular risk. For solid tumours and haematological malignancies, analysis was differentiated by recency of treatment ((≤6 months, 6–12 months, and >12 months); for solid organ transplant recipients, analysis was differentiated by recency of transplantation (≤1 year and >1–2 years prior and on anti-rejection therapy). Data was compared with a non-immunocompromised control group.

The study population amounted to 11,990,730 individuals. 470,910 were classified as immunocompromised in some respect (a prevalence of 3.9% broadly defined immunocompromised and 0.7% stringently defined immunocompromised); 11,519,820 were immunocompetent. Baseline characteristics of study groups are presented in Table 1.

**Table 1: Baseline characteristics of the study population**


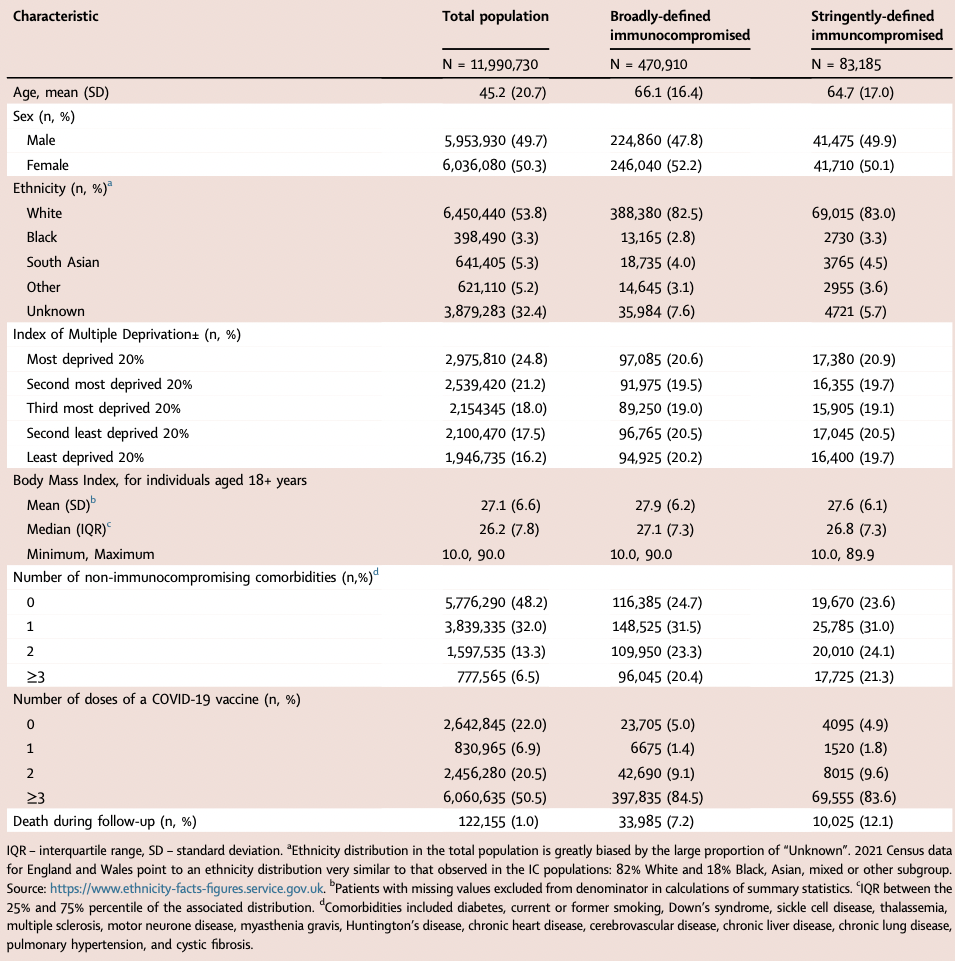


As per Table 2 and 3, a total of 20,910 COVID-19 hospitalisations, 440 ICU admissions and 4810 COVID-19 deaths were recorded for the overall study population. Among the immunocompromised, these figures were 6-fold higher for broadly defined individuals and 11-fold higher for stringently defined immunocompromised than the non-immunocompromised comparator group. Incidence rate ratios remained elevated for both categories even after adjusting for age, sex, and the number of non-immunocompromising comorbidities. Among specific immunocompromised subgroups, aIRRs were highest for stem cell transplantation in the last 2 years (14.24, 95% CI 10.15-19.97) and solid organ transplantation (11.6, 95% CI 10.10-13.31). Immunocompromised individuals were disproportionately represented in severe COVID outcome data. Despite comprising just 3.9% of the total study population, broadly defined immunocompromised represented 21.9% of hospitalisations, 28.1% of ICU admissions and 23.8% of deaths; meanwhile, stringently defined immunocompromised (0.7% of the overall population) represented 8% of hospitalisations, 16.5% of ICU admissions and 7.4% of deaths. These values inflated when only looking at recipients of at least 3 vaccine doses.

**Table 2: COVID-19 related hospitalisations in the study population**


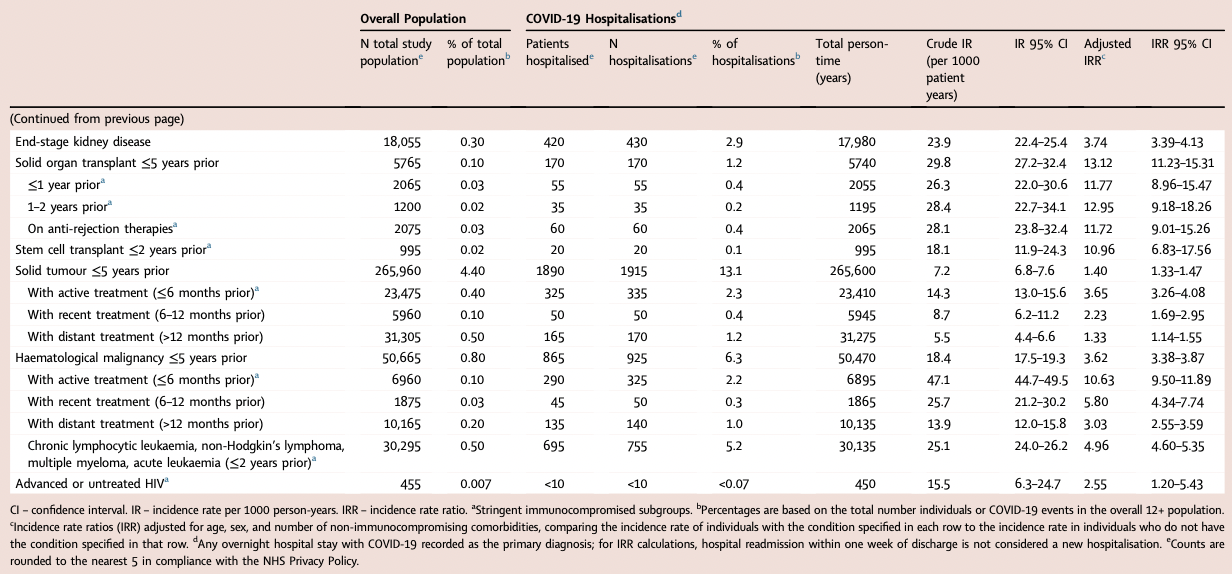

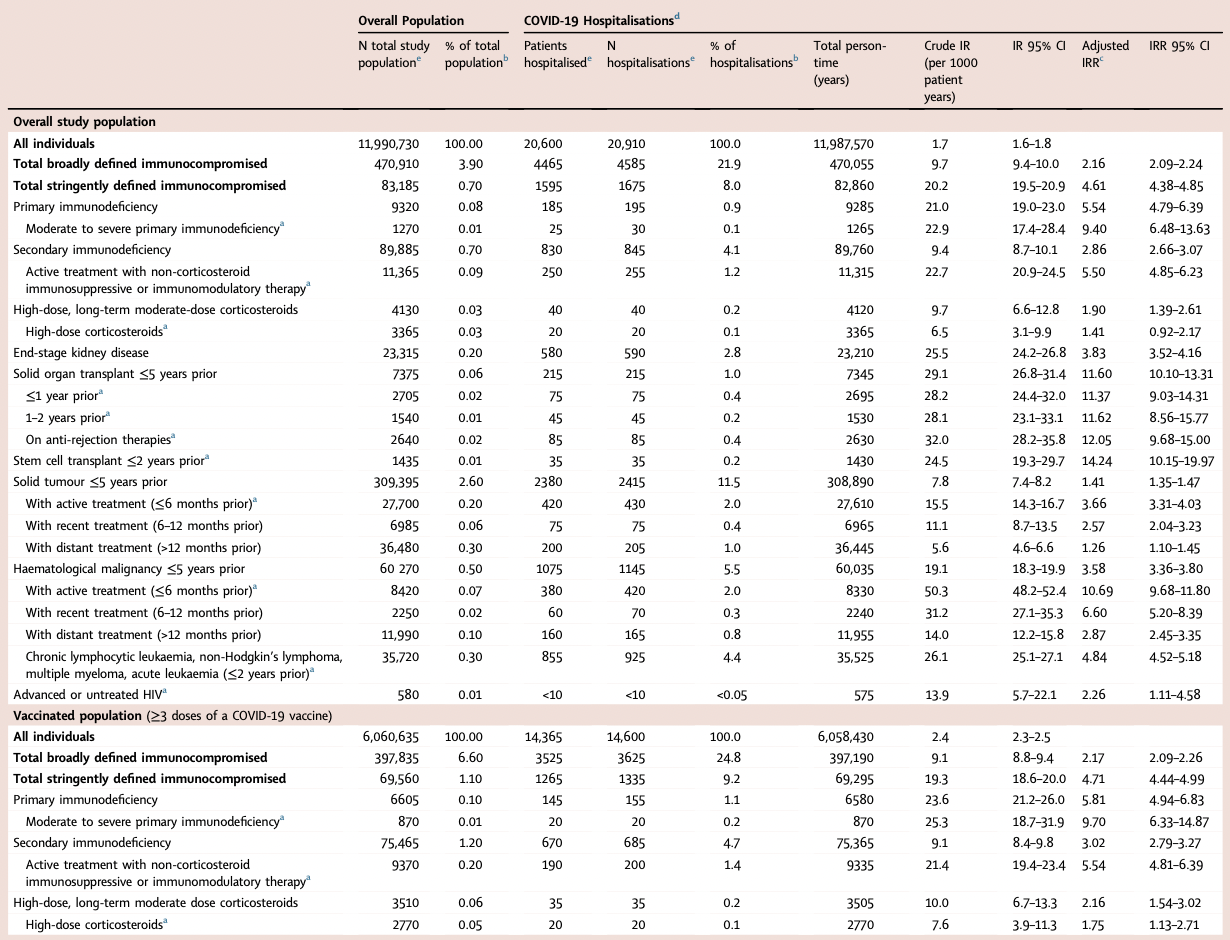


**Table 3: COVID-19 related ICU admissions and mortality in the study population**


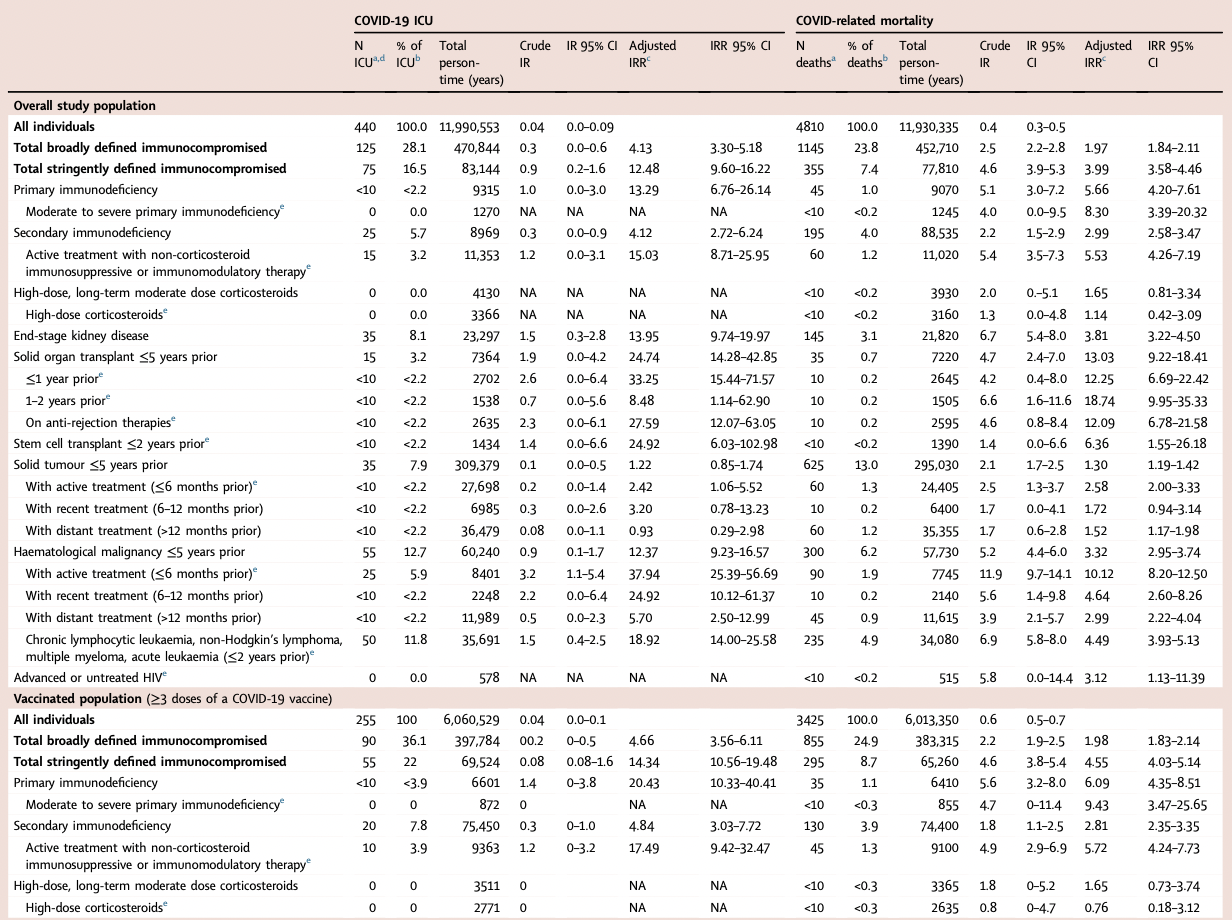


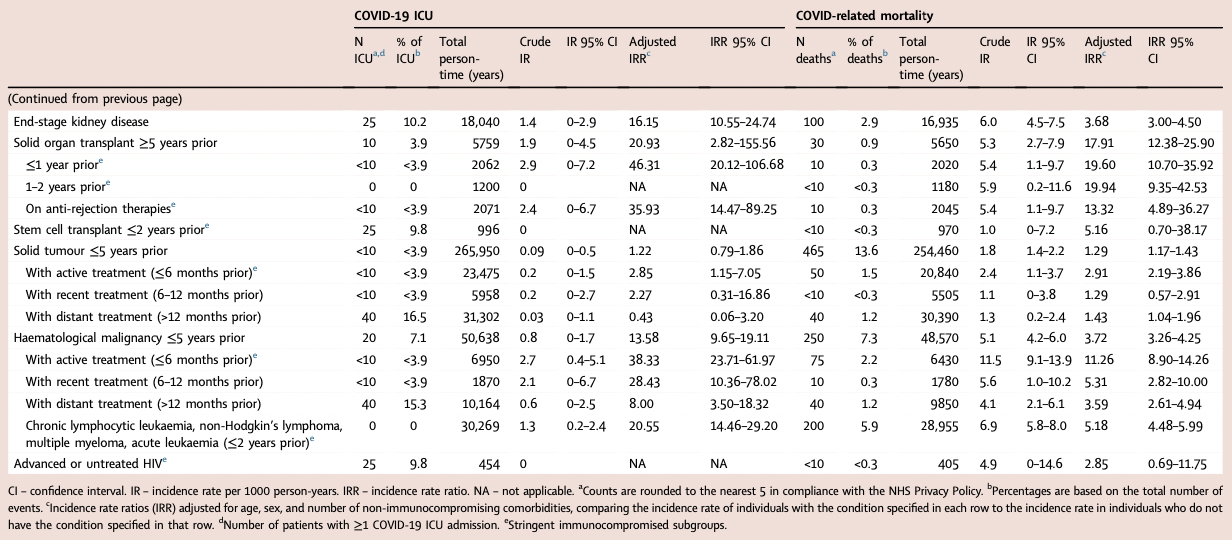


In sum, this work provides clear evidence for the continued vulnerability of the immunocompromised to COVID-19; these individuals represented over 20% of COVID-related hospitalisations, ICU admissions and mortality despite a 3 dose or greater vaccine uptake of over 80%. Although UK pandemic guidelines prioritised the immunosuppressed during its acute phase, more can be done to better target these guidelines to achieve appropriately risk-prioritised policies and recommendations.

**Executive Summary III:** [***‘Immune responses and clinical outcomes following COVID-19 vaccination in patients with immune suppressive diseases (The OCTAVE Study)’***](https://www.ncbi.nlm.nih.gov/pmc/articles/PMC10353927/#:~:text=Population%20studies%20including%20immune%2Dsuppressed,19%20and%20death27%2C28.)

It is well-established that, globally, vaccines against COVID-19 have saved millions of lives. However, while these vaccines continue to confer high protection against critical outcomes, they do not necessarily prevent infection.

The clinical trials and population studies that have investigated the effectiveness of COVID-19 vaccines are generally focused amongst those without pre-existing conditions. This reveals very little about the vaccine outcomes of those with impaired immune systems – patients that we know face parallel risks of severe COVID-19 outcomes and suboptimal vaccine response.

The **O**bservational **C**ohort trial **T** cells, **A**ntibodies and **V**accine **E**fficacy in SARS-CoV-2 (OCTAVE) study was launched to measure COVID-19 vaccine response in patients with different forms of immunosuppression. 2,686 patients were recruited from 11 participating hospitals across the United Kingdom. This cohort included the following patient types:

- Immunosuppressive therapy recipients (for solid cancer, haematological malignancy, ANCA-associated vasculitis on rituximab, inflammatory arthritis, autoimmune liver disease, liver and kidney transplantation, inflammatory bowel disease, ulcerative colitis and undefined inflammatory bowel disease)
- Patients receiving autologous and allogenic hematopoietic stem cell transplant (auto-HSCT and allo-HSCT)
- Patients treated with chimeric antigen receptor (CAR) T cells
- Patients with disease states known to modulate immune responses intrinsically (end-stage kidney disease receiving haemodialysis with or without immunosuppression and patients with advanced liver disease)

All patients had received two vaccine doses - vaccinated with mRNA (BNT162b2 or mRNA-1273) or ChAdOx1 nCov-19 according to UK government-recommended scheduling. Vaccine response was evaluated before and after homologous first dose (V1) and second dose (V2) via antibody and T cell proxies. Responses to variants of concern within patient blood and saliva were also monitored. This data was compared to that of age, sex, prior COVID-19 infection, and vaccine matched healthy controls. Uniquely to this study, antibody and T-cell responses were measured in the same location; direct comparisons could therefore be made between patient groups to identify those most vulnerable to vaccine non-response.

Previous infection, identified via the presence of anti-nucleocapsid antibodies at baseline, was identified in 14% of immunosuppressed patients, with significantly higher rates in those receiving haemodialysis (49%). Two-dose vaccine response could only be evaluated in 2,204 patients and 225 matched controls, however. Seropositivity was denoted as anti-receptor-binding domain (RBD) antibody titers of ≥ 0.8 AU ml−1 after the second dose. By this measure, 99% of healthy controls were seropositive versus 88% of immunosuppressed patients – as illustrated in Figure 1. Seroconversion was lowest in ANCA-associated vasculitis patients receiving rituximab; here only 28% of patients successfully seroconverted. This compares to 50% of CAR-T recipients, 69% of kidney transplant recipients, 75% of liver transplant recipients, 80% of haemodialysis patients, 85% of auto-HSCT recipients and 86% of allo-HSCT recipients. All other immunosuppressed groups under observation returned similar rates of seropositivity to healthy controls; 100% of cirrhosis, Crohn’s disease and ulcerative colitis patients seroconverted, for example. In all, 27% of our immunosuppressed cohort generated low levels of antibodies compared to the healthy group of volunteers, and 12% of patients (around 1 in 10 patients) failed to develop any measurable COVID-19 antibodies at all. Likewise, 12% of immunosuppressed patients failed to generate T-cells against COVID-19; lowest T cell response was observed in patients with kidney disease and liver and kidney transplant recipients.

**Figure 1: Anti-SARS-CoV-2 RBD total Ig responses in whole OCTAVE cohort at post V2 timepoint**


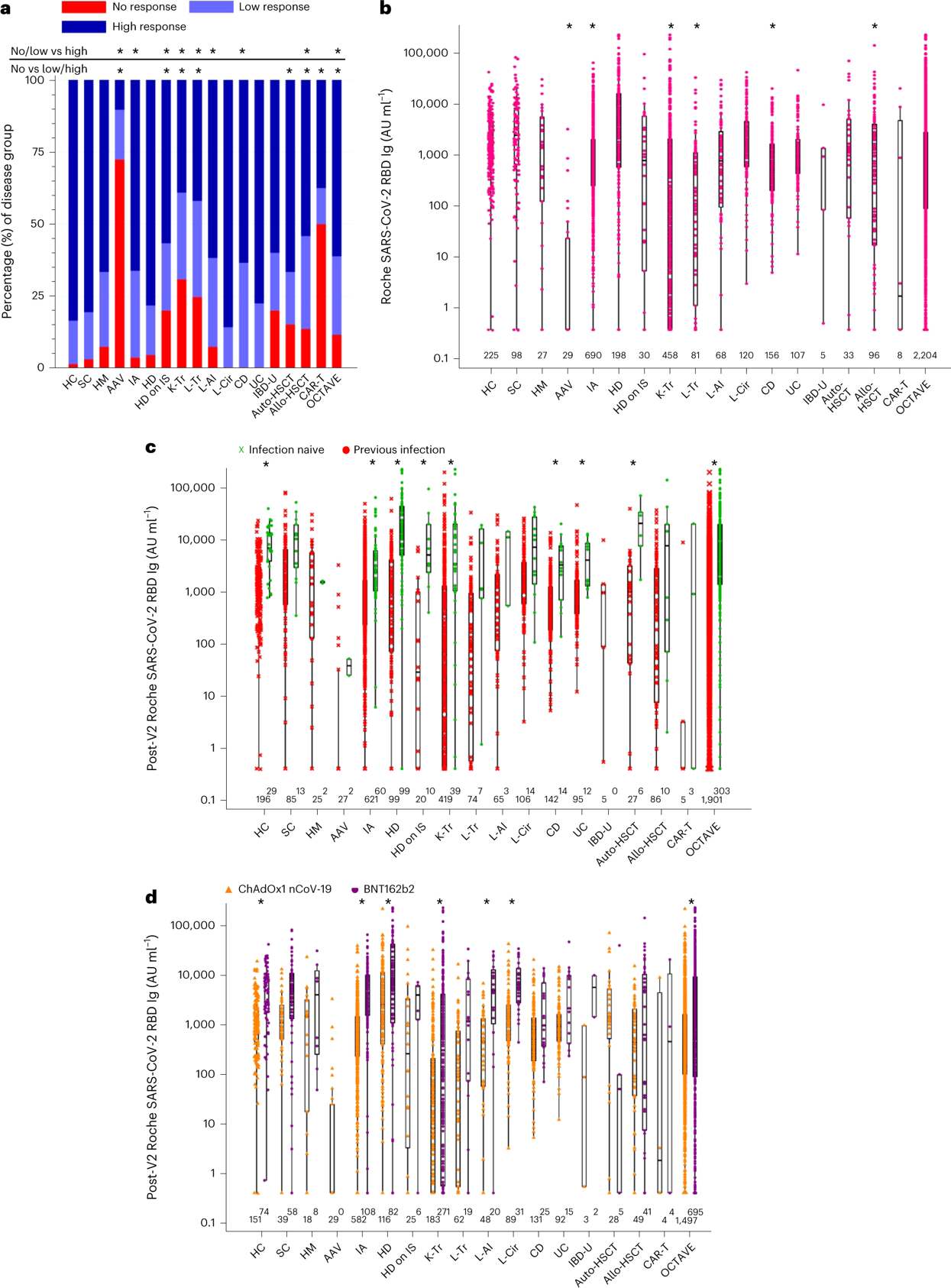


**a,** Proportion of group 1 and group 2 non (<0.8 AU ml−1), low (<380 AU ml−1) and high (>380 AU ml−1) anti-SARS-CoV-2 spike RBD total Ig responses. Statistical comparisons of the proportion of low and no versus high response and no versus low and high response in disease groups compared to healthy controls are presented. **b,** Magnitude of serological response in disease groups and healthy controls. Statistical comparisons comparing disease group to healthy controls are presented. **c,** Anti-SARS-CoV-2 spike RBD total Ig responses comparing previously infected with infection-naive patents. Statistical comparison of infection-naive individuals and previously infected individuals within each group is presented. **d,** Anti-SARS-CoV-2 spike RBD total Ig responses separated by vaccine type. Statistical comparison of vaccine type in each disease group is presented. Unpaired statistical comparison was made on all groups using a two-sided Kruskal–Wallis with post hoc Dunn’s testing. Comparisons of proportions were performed using χ2 or Fisher’s exact tests adjusted for significance using Bonferroni correction (adjusted alpha = 0.003). Only significant comparisons are presented. * indicates statistically significant by Bonferroni-adjusted alpha. Boxes represent median and IQR; whiskers represent ±1.5× IQR.AAV, ANCA-associated vasculitis; CD, Crohn’s disease; HC, healthy controls; HD, hemodialysis; HD on IS, hemodialysis on immunosuppression; HM, hemotological malignancy; IA, inflammatory arthritis; L-AI, autoimmune hepatitis; L-Cir, liver cirrhosis; L-Tr, liver transplant; SC, solid cancer; UC, ulcerative colitis.

When assessing the cross-reactivity of SARS-CoV-2 spike ancestral antibody responses to various variants of concern (Alpha [B.1.1.7], Beta [B.1.351], Gamma [P.1], Delta [B.1.617.2] and Omicron [B.1.1.529 and BA.1]), median spike IgG and spike-ACE-2 binding was significantly decreased to all variants except Alpha in all immunosuppressed groups assessed (liver transplant, autoimmune liver disease, cirrhosis, inflammatory arthritis cohorts). This was most apparent for the Omicron BA.1 variant, though previously infected patients were better able to neutralise. When comparing vaccines, mRNA recipients demonstrated higher antibody but lower T cell responses compared to ChAdOx1 nCov-19 counterparts.

Over the course of the study, 474 patients became infected with COVID-19; of these, 33 were admitted to hospital and 15 died. There was a higher rate of infection amongst patients with absent serological or T cell responses compared to those with high levels of response.

Overall, the OCTAVE study identifies patient groups that exhibit impaired response to COVID-19 vaccines. Failure to seroconvert is also linked to severe disease in this work. That said, it is reassuring that the majority of immunosuppressed patients generate robust immune responses to vaccination, at least proxied by antibody production and T-cell activity. The priority now is to ensure that those identified as being at greatest risk for poor seroconversion are appropriately prioritised for booster doses and alternative therapeutic strategies.

**Executive Summary IV:** [*‘****Consensus position statement on advancing the standardised reporting of infection events in immunocompromised patients’***](https://www.thelancet.com/journals/laninf/article/PIIS1473-3099(23)00377-8/fulltext#:~:text=The%20standardised%20reporting%20of%20infectious,research%20in%20this%20patient%20group.)

The immunocompromised research landscape is rife with inconsistency, especially in respect to reporting on their disease outcomes. Different approaches to detecting, diagnosing, classifying, and dismissing infection events have led to significant interstudy heterogeneity. Even with the introduction of standardised reporting tools and regular data audits, largescale research efforts in immunocompromised infectivity have still failed to document the aetiology and causative pathogen in 40-60% of infection-related deaths. Poor categorisation and limited codification of immunocompromised infection events have also undermined researcher ability to pool or differentiate what data is available. This has knock on implications for directing patient care or public policy.

This effort was designed to identify workable middle ground between an idealised set of immunocompromised infection reporting criteria and that which would be both feasible and convenient for researchers to adopt in a busy clinical or study environment. A study group of experts in infection outcomes in the immunocompromised convened in Lisbon, Portugal (during the European Congress of Clinical Microbiology and Infectious Diseases 2022) to discuss these issues, conduct a literature search for existing reporting guidelines and identify the core minimum reporting items that could harmonise these offerings. Study group members were affiliated with a number of global immunosuppressed societies – all of which ratified the final position statement. These included (1) the International Immunocompromised Host Society; (2) the European Society of Clinical Microbiology and Infectious Diseases Study Group for Infections in Compromised Hosts; (3) the European Conference on Infections in Leukaemia; (4) the European Society for Blood and Marrow Transplantation Infectious Diseases Working Party; (5) the Transplant Infectious Disease Section of The Transplantation Society; (6) the Infectious Disease Community of Practice of the American Society of Transplantation; and (7) the Immunocompromised Host Special Interest Group of the Australasian Society for Infectious Diseases.

The study group established that a core minimum reporting dataset (abbreviated as SCORE and provided in Table 1) for collecting information about infections in the immunocompromised based on strengths of available clinical, microbiological or radiological evidence and must include:

- Site of infection
- Severity of infection, qualified by Grades 1 through 5 (‘localised, requiring no intervention’ to ‘death from or with infection’).
- Category of infection with pathogen (organism) classification (bacterial, viral, fungal, other)
- Infection outcomes

**Table 1: Minimum core reporting dataset**


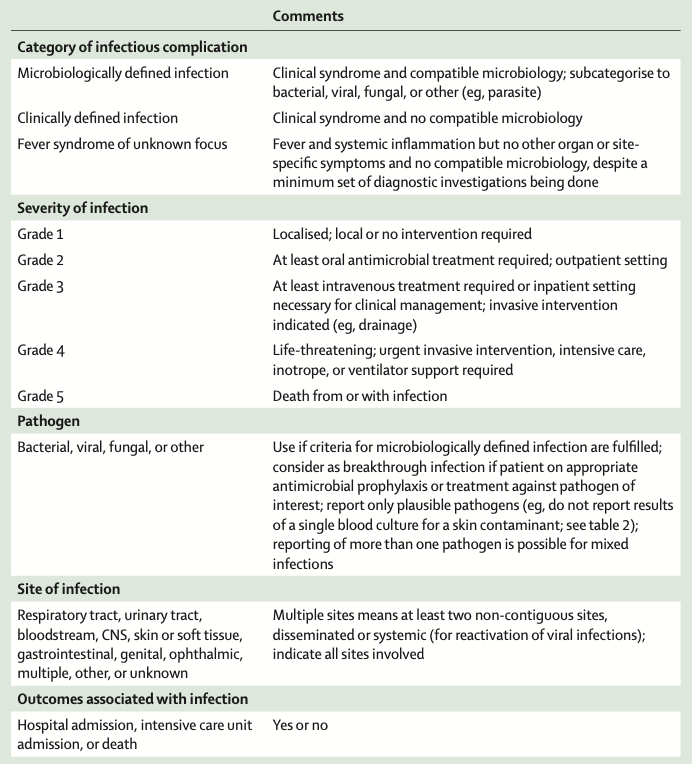


SCORE criteria flex to accommodate additional variables depending on the nature of the research. For example, SCORE criteria for randomised control trials recommending collecting more contextual information, including but not limited to data on infection time course (ongoing vs historical) and grouping (e.g. upper respiratory tract infection), as well as detail on the prophylactic policies utilised by participating centres and the rate of uptake of these. All definitions used to diagnose and classify the patient and their infection (e.g. type, syndrome, and severity) during the trial must also be clearly cited. This ensures participating sites are utilising and reporting against the same definitions. For observational studies, meanwhile, the expert group recommends enhancing the core offering to include data collection on patient treatment history (e.g. chemotherapy) and the clinical factors that could be contributing to infection risks. Pathogen-centric studies should report on detailed pathogen descriptors (e.g. methods and results of susceptibility testing). For immunocompromised patients, the additional challenges when it comes to clinical interpretation of microbiological results were highlighted. These recommendations both standardise and enhance current reporting on infections amongst immunosuppressed patients and subsequent health outcomes.

However, it is acknowledged that many research centres do not have access to the full battery of diagnostic tests that would be required to meet enhanced SCORE criteria. Conversely, the study group acknowledges that future iterations will also have to accommodate novel testing technologies, including genomic-based or metabolomic-based pathogen detection, for those institutions where this is standard practice. Better data capture methods must also be explored as a matter of urgency: collecting standardised infection data from electronic medical records has significant prospective value for precision research. At present, free text, inaccurate or unstructured data entry have undermined these efforts and the codification and linkage of laboratory results have been especially poor. Harnessing natural language processing is one recommendation to facilitate these improvements - a technology that has only very recently been applied to immunocompromised patient data.

Despite the limitations recognised, as a collective, the collective expert groups identified a standardised method for reporting infections events and associated complications in the immunocompromised. If adopted widely, this will have significant prospective benefits for the diagnosis and treatment of immunocompromised patients and, in turn, the targeting of infection prevention strategies for this diverse group.

**A brief introduction to digital phenotyping**

A digital phenotype is a machine-readable taxonomy, made up of relationships and rules that can be applied to computerised medical records (CMRs) in algorithmic format to extract specific cohorts or events of interest. Rules are comprised of logical expressions and data elements – in this instance, relevant clinical codes or concepts – and are implemented into CMR systems via computerised queries.

In this way, digital phenotypes can be constructed for all manner of clinical populations, procedures, and events. In the spirit of open science, these resources can be shared between organisations to expedite research efforts. A range of public phenotype libraries have been created for this purpose.

However, organisations differ in their approach to phenotype development. At the Royal College of General Practitioners (RCGP) Research and Surveillance Centre (RSC), the English Primary Care Sentinel Network, this process is traditionally three-step. As elaborated upon below and illustrated in Figure 1, a conceptual phenotype is processed via an ontological layer, a coding layer, and a logical data extract layer – all of which are quality assured by additional team members.

1. The ontological layer - a human-readable description of key concepts within, and relationships between, a given phenotype.
2. The coding layer – where conceptual phenotype layers are translated into their constituent clinical codes and thematic categories.
3. The logical data extract layer – where ontological rules, expressing the relationships between phenotype levels and their respective eligibility criteria, are converted into Structured Query Language (SQL) expressions to extract desired data from the overall pool(s) available.

Once this processing is complete, phenotype-derived extracts are tested for face validity and compared to reference sources. Major disparities at this stage are used to refine previous layers’ input. Those that pass this additional scrutiny are then uploaded into the aforementioned libraries and implemented into real-world evidence.

**Figure 1: A three-layered approach to instituting a novel phenotype, as implemented by the RCGP RSC**

**
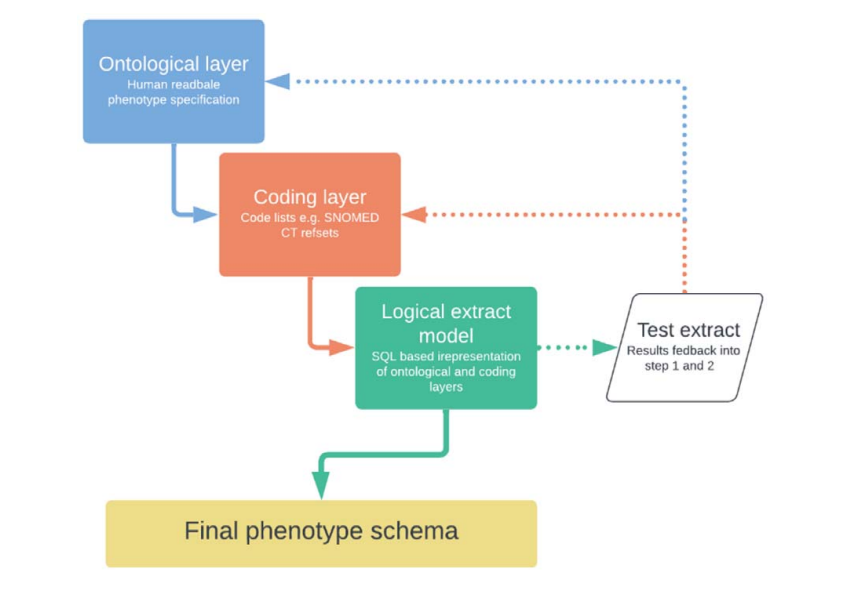
**

Visualised in Figure 2, this process was applied to convert a COVID risk-stratified taxonomy for adult immunosuppression into a multi-level, CMR compatible phenotype. Serving as the ontological layer in this example, this taxonomy surfaced through rapid literature review into differential COVID-19 mortality amongst the immunosuppressed (as classified by Chapter 14a of the *Immunisation against infectious disease: The Green Book*.). Out of this exercise, the following four levels were identified as most valuable for subdividing the immunosuppressed in descending order of vulnerability to COVID-19: 1) Bone marrow compromising conditions, 2) Solid Organ Transplant recipients, 3) Immunosuppressive treatment recipients and 4) Immunodeficiency.

The coding layer, meanwhile, leveraged existing RSC data assets and clinical risk group codelists from the Primary Care Information Services (PRIMIS) at the University of Nottingham. Here, immunosuppressed concepts from pre-curated RSC cancer reference sets and PRIMIS versions 1.3 and 2.3 (all designated in Systematised Nomenclature of Medicine Clinical Terms [SNOMED CT]) were aggregated, refined, and subdivided to create the valueset that would target data extraction. Finally, a logical extract model was constructed to incorporate the time and dose conditions of immunosuppression specified in The Green Book. Hierarchical principles and mutual exclusivity were also codified to ensure that, once applied within real-world data, this pilot immunosuppressed phenotype would only allocate patients into the highest risk level that they were eligible. Now fully curated, this pilot phenotype is ready to extract immunosuppressed populations from SNOMED CT compatible databases and subdivide desired outcomes by level of COVID risk.

Figure 2: Clinical logic flow of the pilot immunosuppressed phenotype


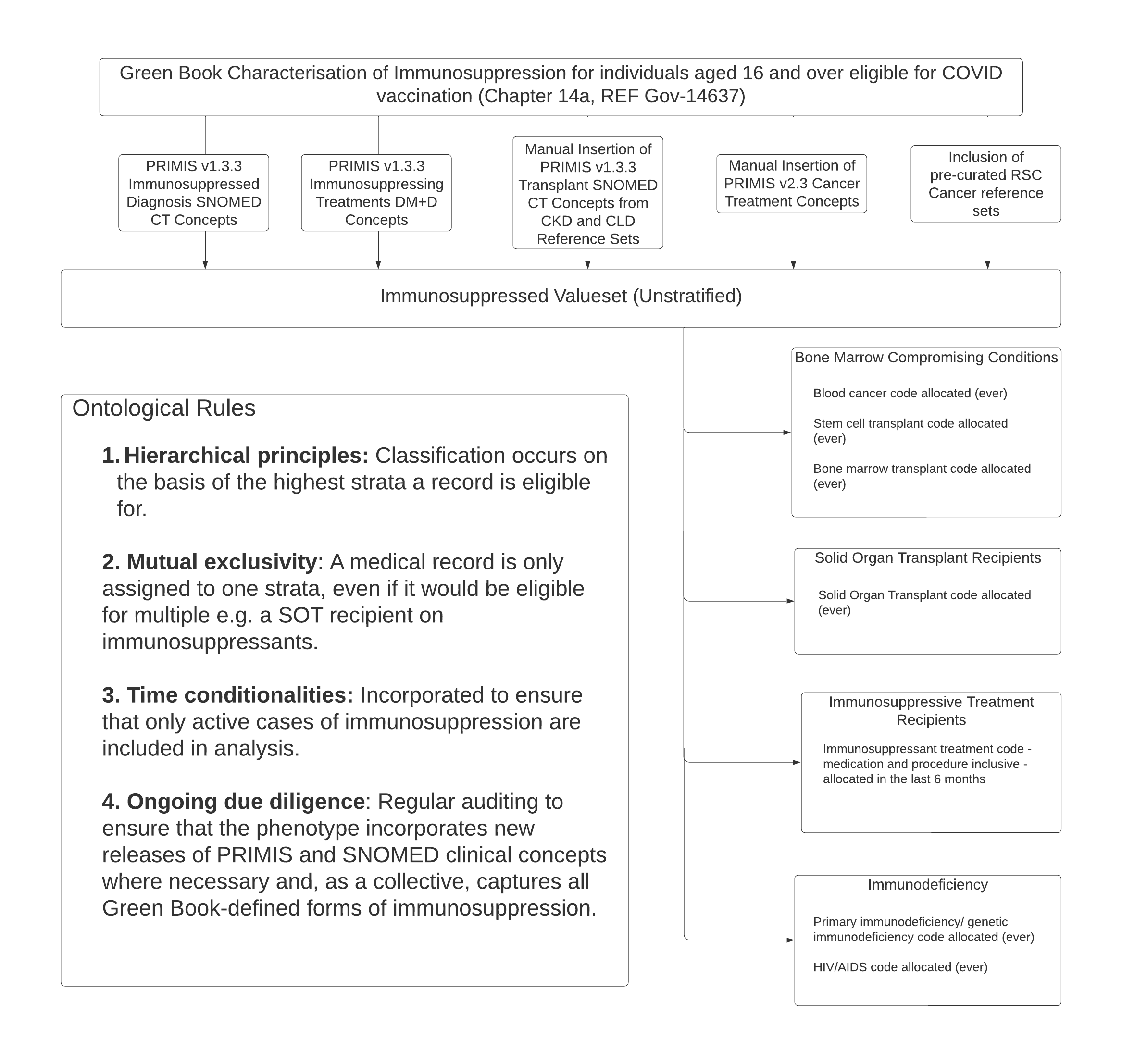


It is our intention to leverage the collective expertise of the eDelphi panel to construct a more definitive COVID risk-stratified phenotype for adult immunosuppression. We will present panellists with draft taxonomies – one multi-level (as above), the other higher vs lower risk categorised - that will be refined through each round of eDelphi questioning. The final discussion group will be an opportunity for panellists to voice any remaining points of disagreement before these taxonomies are converted into digital phenotypes as above.

Once machine-readable, these eDelphi phenotypes for adult immunosuppression will be incorporated as standard into RSC dataflows to enhance disease surveillance and pharmacovigilance amongst this diverse clinical risk group.


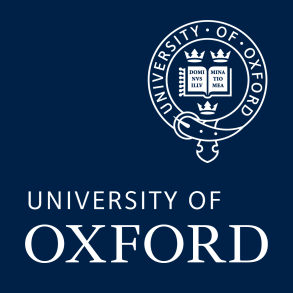


Meredith Leston

Nuffield Department of Primary Care Health Sciences

Eagle House

7 Walton Well Road

Oxford, OX2 6ED

[Meredith.leston@phc.ox.ac.uk](mailto:Meredith.leston@phc.ox.ac.uk)

+447896980320

**CONSENT FORM**

**The inaugural UK eDElphi STudy to DefINe and Risk-Stratify ImmunosupprESsion (The DESTINIES Study)**

*Name of Researcher: Meredith Leston If you agree, please initial box*

| 1. I confirm that I have read all pre-read materials for this study. |  |
| --- | --- |
| 1. I confirm that I have had the opportunity to consider the information, ask questions, and have these answered satisfactorily. |  |
| 1. I understand that my participation is voluntary and that I am free to withdraw at any time without giving any reason. |  |
| 1. I agree to the final discussion group being audio recorded. |  |
| 1. I agree to take part in this study. |  |

| *_______________________* | *_________________* | *___________________________* |
| --- | --- | --- |
| *Name of Participant* | *Date* | *Signature* |
| Meredith Leston | 18.04.24 | 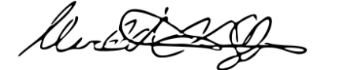 |
| *Name of Person taking Consent* | *Date* | *Signature* |

1. **Questionnaire items**

Round 1, n = 64

- *Appraisal of candidate diagnoses’ appropriateness for inclusion in a digital phenotype for ‘adult immunosuppression’, higher versus lower risk status for COVID-19 severe outcomes and excess vulnerability compared to the immunocompetent.*
- *Opportunity for panellists to recommend additional diagnoses for consideration.*
- *Assessment of a range of clinical consensus statements.*

| Please enter your Panellist ID number. |
| --- |
| Q1a: To what extent do you agree with the following statements? [Immunosuppression is poorly defined] |
| Q1a: To what extent do you agree with the following statements? [Our current clinical definition for immunosuppression is too expansive] |
| Q1a: To what extent do you agree with the following statements? [Our current clinical definition for immunosuppression is not expansive enough] |
| Q1a: To what extent do you agree with the following statements? [The terms 'immunosuppression' and 'immunocompromised' can be used interchangeably] |
| Q1a: To what extent do you agree with the following statements? [The needs of the immunosuppressed are sufficiently prioritised in times of public health emergency] |
| Q1a: To what extent do you agree with the following statements? [COVID-19 no longer poses a real risk to the immunosuppressed] |
| Q1a: To what extent do you agree with the following statements? [Vulnerability to COVID-19 is similar for all immunosuppressed subgroups] |
| Q1a: To what extent do you agree with the following statements? [It is easy to pick out the immunosuppressed subgroups at most risk for COVID-19] |
| Q1a: To what extent do you agree with the following statements? [The immunosuppressed subgroups that are at the most risk for COVID-19 are the same subgroups at most risk for other infectious diseases (RSV, influenza, bacterial pneumonia etc.)] |
| Q1a: To what extent do you agree with the following statements? [COVID care for the immunosuppressed – including vaccination and the release of antivirals and other resources – is overly one size fits all] |
| Q1b: Please justify your answers to Q1a, specifying if there were any areas where you were uncertain and why. |
| Q2a: The following conditions are explicitly referenced in the UK characterisation of immunosuppression. In your expert opinion, how appropriate is their inclusion? [Solid organ transplantation] |
| Q2a: The following conditions are explicitly referenced in the UK characterisation of immunosuppression. In your expert opinion, how appropriate is their inclusion? [Bone marrow transplantation] |
| Q2a: The following conditions are explicitly referenced in the UK characterisation of immunosuppression. In your expert opinion, how appropriate is their inclusion? [Stem cell transplantation] |
| Q2a: The following conditions are explicitly referenced in the UK characterisation of immunosuppression. In your expert opinion, how appropriate is their inclusion? [Haematological malignancies] |
| Q2a: The following conditions are explicitly referenced in the UK characterisation of immunosuppression. In your expert opinion, how appropriate is their inclusion? [HIV infection at all stages] |
| Q2a: The following conditions are explicitly referenced in the UK characterisation of immunosuppression. In your expert opinion, how appropriate is their inclusion? [Genetic disorders affecting the immune system (e.g. SCID, complement disorder, IRAK-4, NEMO)] |
| Q2a: The following conditions are explicitly referenced in the UK characterisation of immunosuppression. In your expert opinion, how appropriate is their inclusion? [Systemic lupus erythematosus] |
| Q2a: The following conditions are explicitly referenced in the UK characterisation of immunosuppression. In your expert opinion, how appropriate is their inclusion? [Rheumatoid arthritis] |
| Q2a: The following conditions are explicitly referenced in the UK characterisation of immunosuppression. In your expert opinion, how appropriate is their inclusion? [Inflammatory bowel disease] |
| Q2a: The following conditions are explicitly referenced in the UK characterisation of immunosuppression. In your expert opinion, how appropriate is their inclusion? [Scleroderma] |
| Q2a: The following conditions are explicitly referenced in the UK characterisation of immunosuppression. In your expert opinion, how appropriate is their inclusion? [Psoriasis] |
| Q2b: Please justify your answers to Q2a, specifying if there were any areas where you were uncertain and why. |
| Q3a: The following conditions are not explicitly referenced in the UK characterisation of immunosuppression but appear in comparable national and international resources. In your expert opinion, how appropriate would it be to include them going forwards? [Multi-organ transplantation] |
| Q3a: The following conditions are not explicitly referenced in the UK characterisation of immunosuppression but appear in comparable national and international resources. In your expert opinion, how appropriate would it be to include them going forwards? [Islet transplantation] |
| Q3a: The following conditions are not explicitly referenced in the UK characterisation of immunosuppression but appear in comparable national and international resources. In your expert opinion, how appropriate would it be to include them going forwards? [Solid tumours] |
| Q3a: The following conditions are not explicitly referenced in the UK characterisation of immunosuppression but appear in comparable national and international resources. In your expert opinion, how appropriate would it be to include them going forwards? [Generalised malignancies (metastasis)] |
| Q3a: The following conditions are not explicitly referenced in the UK characterisation of immunosuppression but appear in comparable national and international resources. In your expert opinion, how appropriate would it be to include them going forwards? [Drug-managed HIV] |
| Q3a: The following conditions are not explicitly referenced in the UK characterisation of immunosuppression but appear in comparable national and international resources. In your expert opinion, how appropriate would it be to include them going forwards? [Untreated HIV] |
| Q3a: The following conditions are not explicitly referenced in the UK characterisation of immunosuppression but appear in comparable national and international resources. In your expert opinion, how appropriate would it be to include them going forwards? [AIDS] |
| Q3a: The following conditions are not explicitly referenced in the UK characterisation of immunosuppression but appear in comparable national and international resources. In your expert opinion, how appropriate would it be to include them going forwards? [Down Syndrome] |
| Q3a: The following conditions are not explicitly referenced in the UK characterisation of immunosuppression but appear in comparable national and international resources. In your expert opinion, how appropriate would it be to include them going forwards? [Autoimmune skin diseases (e.g. Dermatitis herpetiformis, Vitiligo, Pemphigus, Dermatomyositis, etc.)] |
| Q3a: The following conditions are not explicitly referenced in the UK characterisation of immunosuppression but appear in comparable national and international resources. In your expert opinion, how appropriate would it be to include them going forwards? [Rheumatologic Disorders (e.g. Sjögren's syndrome, Ankylosing spondylitis, Gout etc.)] |
| Q3a: The following conditions are not explicitly referenced in the UK characterisation of immunosuppression but appear in comparable national and international resources. In your expert opinion, how appropriate would it be to include them going forwards? [Gastrointestinal Autoimmune Diseases (e.g. Celiac disease, Ulcerative colitis, Autoimmune hepatitis)] |
| Q3a: The following conditions are not explicitly referenced in the UK characterisation of immunosuppression but appear in comparable national and international resources. In your expert opinion, how appropriate would it be to include them going forwards? [Endocrine Autoimmune disorders (e.g. Type 1 diabetes mellitus, Hashimoto’s thyroiditis, Graves' disease, Addison’s disease etc.)] |
| Q3a: The following conditions are not explicitly referenced in the UK characterisation of immunosuppression but appear in comparable national and international resources. In your expert opinion, how appropriate would it be to include them going forwards? [Neurological Autoimmune Conditions (e.g. Multiple sclerosis, Myasthenia gravis, Autoimmune encephalitis, Guillain-Barré syndrome etc.)] |
| Q3a: The following conditions are not explicitly referenced in the UK characterisation of immunosuppression but appear in comparable national and international resources. In your expert opinion, how appropriate would it be to include them going forwards? [Hematological Autoimmune Disorders (e.g. Evan’s syndrome, Immune thrombocytopenic purpura, Antiphospholipid syndrome etc.)] |
| Q3a: The following conditions are not explicitly referenced in the UK characterisation of immunosuppression but appear in comparable national and international resources. In your expert opinion, how appropriate would it be to include them going forwards? [Renal Autoimmune Diseases (e.g. Lupus nephritis, Glomerulonephritis, IgA nephropathy etc.)] |
| Q3a: The following conditions are not explicitly referenced in the UK characterisation of immunosuppression but appear in comparable national and international resources. In your expert opinion, how appropriate would it be to include them going forwards? [Opthalmologic autoimmune conditions (e.g. Uveitis, Graves’ ophthalmopathy etc.)] |
| Q3a: The following conditions are not explicitly referenced in the UK characterisation of immunosuppression but appear in comparable national and international resources. In your expert opinion, how appropriate would it be to include them going forwards? [Underlying aberrant immunity (e.g. graft-vs-host disease, graft rejection, absent or incomplete immune reconstitution, neutropenia ANC, lymphopenia ALC)] |
| Q3a: The following conditions are not explicitly referenced in the UK characterisation of immunosuppression but appear in comparable national and international resources. In your expert opinion, how appropriate would it be to include them going forwards? [Type 2 diabetes mellitus] |
| Q3a: The following conditions are not explicitly referenced in the UK characterisation of immunosuppression but appear in comparable national and international resources. In your expert opinion, how appropriate would it be to include them going forwards? [Asplenia (anatomic/functional)] |
| Q3a: The following conditions are not explicitly referenced in the UK characterisation of immunosuppression but appear in comparable national and international resources. In your expert opinion, how appropriate would it be to include them going forwards? [Aplastic anemia] |
| Q3a: The following conditions are not explicitly referenced in the UK characterisation of immunosuppression but appear in comparable national and international resources. In your expert opinion, how appropriate would it be to include them going forwards? [Sickle cell disease] |
| Q3a: The following conditions are not explicitly referenced in the UK characterisation of immunosuppression but appear in comparable national and international resources. In your expert opinion, how appropriate would it be to include them going forwards? [Chronic kidney disease] |
| Q3a: The following conditions are not explicitly referenced in the UK characterisation of immunosuppression but appear in comparable national and international resources. In your expert opinion, how appropriate would it be to include them going forwards? [Nephrotic syndrome] |
| Q3a: The following conditions are not explicitly referenced in the UK characterisation of immunosuppression but appear in comparable national and international resources. In your expert opinion, how appropriate would it be to include them going forwards? [Dialysis] |
| Q3a: The following conditions are not explicitly referenced in the UK characterisation of immunosuppression but appear in comparable national and international resources. In your expert opinion, how appropriate would it be to include them going forwards? [Cerebrospinal fluid leak] |
| Q3a: The following conditions are not explicitly referenced in the UK characterisation of immunosuppression but appear in comparable national and international resources. In your expert opinion, how appropriate would it be to include them going forwards? [Cochlear implant] |
| Q3a: The following conditions are not explicitly referenced in the UK characterisation of immunosuppression but appear in comparable national and international resources. In your expert opinion, how appropriate would it be to include them going forwards? [Burn injuries] |
| Q3a: The following conditions are not explicitly referenced in the UK characterisation of immunosuppression but appear in comparable national and international resources. In your expert opinion, how appropriate would it be to include them going forwards? [Pregnancy] |
| Q3a: The following conditions are not explicitly referenced in the UK characterisation of immunosuppression but appear in comparable national and international resources. In your expert opinion, how appropriate would it be to include them going forwards? [Chronic stress] |
| Q3a: The following conditions are not explicitly referenced in the UK characterisation of immunosuppression but appear in comparable national and international resources. In your expert opinion, how appropriate would it be to include them going forwards? [Sleep deprivation] |
| Q3a: The following conditions are not explicitly referenced in the UK characterisation of immunosuppression but appear in comparable national and international resources. In your expert opinion, how appropriate would it be to include them going forwards? [Malnutrition] |
| Q3a: The following conditions are not explicitly referenced in the UK characterisation of immunosuppression but appear in comparable national and international resources. In your expert opinion, how appropriate would it be to include them going forwards? [Stunting] |
| Q3a: The following conditions are not explicitly referenced in the UK characterisation of immunosuppression but appear in comparable national and international resources. In your expert opinion, how appropriate would it be to include them going forwards? [Anorexia Nervosa] |
| Q3b: Please justify your answers to Q3a, specifying if there were any areas where you were uncertain and why. |
| Q4: Were there any conditions you were expecting to see that were not listed in either Q2 or Q3? If yes, please name them. |
| Q5a: To what extent do you agree with the following statements: Immunosuppression would not be conferred if... [The patient's cancer is in remission] |
| Q5a: To what extent do you agree with the following statements: Immunosuppression would not be conferred if... [The patient's cancer is currently untreated] |
| Q5a: To what extent do you agree with the following statements: Immunosuppression would not be conferred if... [The patient's cancer is classed as Early Stage (Stage I)] |
| Q5a: To what extent do you agree with the following statements: Immunosuppression would not be conferred if... [The patient's HIV is drug-managed] |
| Q5a: To what extent do you agree with the following statements: Immunosuppression would not be conferred if... [The patient's inflammatory disease is currently untreated] |
| Q5a: To what extent do you agree with the following statements: Immunosuppression would not be conferred if... [The patient has received immunosuppressive therapy for less than 6 months] |
| Q5a: To what extent do you agree with the following statements: Immunosuppression would not be conferred if... [The patient's immunosuppressive regimen was discontinued more than 6 months ago] |
| Q5a: To what extent do you agree with the following statements: Immunosuppression would not be conferred if... [The patient's immunosuppressive regimen was discontinued more than 1 year ago] |
| Q5a: To what extent do you agree with the following statements: Immunosuppression would not be conferred if... [The patient is on low-dose immunosuppressives (equivalent of < 20mg of prednisone per day)] |
| Q5b: Please justify your answers to Q5a, specifying if there were any areas where you were uncertain and why. |
| Q6a: Please specify whether you consider the following conditions at higher or lower risk for severe COVID-19 outcomes? [Solid organ transplantation] |
| Q6a: Please specify whether you consider the following conditions at higher or lower risk for severe COVID-19 outcomes? [Bone marrow transplantation] |
| Q6a: Please specify whether you consider the following conditions at higher or lower risk for severe COVID-19 outcomes? [Stem cell transplantation] |
| Q6a: Please specify whether you consider the following conditions at higher or lower risk for severe COVID-19 outcomes? [Multi-organ transplantation] |
| Q6a: Please specify whether you consider the following conditions at higher or lower risk for severe COVID-19 outcomes? [Islet transplantation] |
| Q6a: Please specify whether you consider the following conditions at higher or lower risk for severe COVID-19 outcomes? [Haematological malignancies] |
| Q6a: Please specify whether you consider the following conditions at higher or lower risk for severe COVID-19 outcomes? [Solid tumours] |
| Q6a: Please specify whether you consider the following conditions at higher or lower risk for severe COVID-19 outcomes? [Generalised malignancies (metastasis)] |
| Q6a: Please specify whether you consider the following conditions at higher or lower risk for severe COVID-19 outcomes? [Actively-treated malignancy] |
| Q6a: Please specify whether you consider the following conditions at higher or lower risk for severe COVID-19 outcomes? [HIV infection all stages] |
| Q6a: Please specify whether you consider the following conditions at higher or lower risk for severe COVID-19 outcomes? [Drug-managed HIV] |
| Q6a: Please specify whether you consider the following conditions at higher or lower risk for severe COVID-19 outcomes? [Untreated HIV] |
| Q6a: Please specify whether you consider the following conditions at higher or lower risk for severe COVID-19 outcomes? [AIDS] |
| Q6a: Please specify whether you consider the following conditions at higher or lower risk for severe COVID-19 outcomes? [Genetic disorders affecting the immune system (e.g. SCID, complement disorder, IRAK-4, NEMO)] |
| Q6a: Please specify whether you consider the following conditions at higher or lower risk for severe COVID-19 outcomes? [Down Syndrome] |
| Q6a: Please specify whether you consider the following conditions at higher or lower risk for severe COVID-19 outcomes? [Autoimmune skin diseases (e.g. Psoriasis, Dermatitis herpetiformis, Vitiligo, Pemphigus, Scleroderma, Dermatomyositis, etc.)] |
| Q6a: Please specify whether you consider the following conditions at higher or lower risk for severe COVID-19 outcomes? [Rheumatologic Disorders (e.g. Systemic lupus erythematosus, Rheumatoid Arthritis, Sjögren's syndrome, Ankylosing spondylitis, Gout etc.)] |
| Q6a: Please specify whether you consider the following conditions at higher or lower risk for severe COVID-19 outcomes? [Gastrointestinal Autoimmune Diseases (e.g. Inflammatory Bowel Syndrome, Celiac disease, Ulcerative colitis, Autoimmune hepatitis)] |
| Q6a: Please specify whether you consider the following conditions at higher or lower risk for severe COVID-19 outcomes? [Endocrine Autoimmune disorders (e.g. Type 1 diabetes mellitus, Hashimoto’s thyroiditis, Graves' disease, Addison’s disease etc.)] |
| Q6a: Please specify whether you consider the following conditions at higher or lower risk for severe COVID-19 outcomes? [Neurological Autoimmune Conditions (e.g. Multiple sclerosis, Myasthenia gravis, Autoimmune encephalitis, Guillain-Barré syndrome etc.)] |
| Q6a: Please specify whether you consider the following conditions at higher or lower risk for severe COVID-19 outcomes? [Hematological Autoimmune Disorders (e.g. Evan’s syndrome, Immune thrombocytopenic purpura, Antiphospholipid syndrome etc.)] |
| Q6a: Please specify whether you consider the following conditions at higher or lower risk for severe COVID-19 outcomes? [Renal Autoimmune Diseases (e.g. Lupus nephritis, Glomerulonephritis, IgA nephropathy etc.)] |
| Q6a: Please specify whether you consider the following conditions at higher or lower risk for severe COVID-19 outcomes? [Opthalmologic autoimmune conditions (e.g. Uveitis, Graves’ ophthalmopathy etc.)] |
| Q6a: Please specify whether you consider the following conditions at higher or lower risk for severe COVID-19 outcomes? [Underlying aberrant immunity (e.g. graft-vs-host disease, graft rejection, absent or incomplete immune reconstitution, neutropenia ANC, lymphopenia ALC)] |
| Q6a: Please specify whether you consider the following conditions at higher or lower risk for severe COVID-19 outcomes? [Type 2 diabetes mellitus] |
| Q6a: Please specify whether you consider the following conditions at higher or lower risk for severe COVID-19 outcomes? [Asplenia (anatomic/ functional)] |
| Q6a: Please specify whether you consider the following conditions at higher or lower risk for severe COVID-19 outcomes? [Aplastic anemia] |
| Q6a: Please specify whether you consider the following conditions at higher or lower risk for severe COVID-19 outcomes? [Sickle cell disease] |
| Q6a: Please specify whether you consider the following conditions at higher or lower risk for severe COVID-19 outcomes? [Chronic kidney disease] |
| Q6a: Please specify whether you consider the following conditions at higher or lower risk for severe COVID-19 outcomes? [Nephrotic syndrome] |
| Q6a: Please specify whether you consider the following conditions at higher or lower risk for severe COVID-19 outcomes? [Dialysis] |
| Q6a: Please specify whether you consider the following conditions at higher or lower risk for severe COVID-19 outcomes? [Cerebrospinal fluid leak] |
| Q6a: Please specify whether you consider the following conditions at higher or lower risk for severe COVID-19 outcomes? [Cochlear implant] |
| Q6a: Please specify whether you consider the following conditions at higher or lower risk for severe COVID-19 outcomes? [Burn injuries] |
| Q6a: Please specify whether you consider the following conditions at higher or lower risk for severe COVID-19 outcomes? [Pregnancy] |
| Q6a: Please specify whether you consider the following conditions at higher or lower risk for severe COVID-19 outcomes? [Chronic stress] |
| Q6a: Please specify whether you consider the following conditions at higher or lower risk for severe COVID-19 outcomes? [Sleep deprivation] |
| Q6a: Please specify whether you consider the following conditions at higher or lower risk for severe COVID-19 outcomes? [Malnutrition] |
| Q6a: Please specify whether you consider the following conditions at higher or lower risk for severe COVID-19 outcomes? [Stunting] |
| Q6a: Please specify whether you consider the following conditions at higher or lower risk for severe COVID-19 outcomes? [Anorexia Nervosa] |
| Q6b: Please justify your answers to Q6a, specifying if there were any areas where you were uncertain and why. |
| Q7a: Please specify how vulnerable you consider the following conditions are to severe COVID-19 infection outcomes. [Solid organ transplantation] |
| Q7a: Please specify how vulnerable you consider the following conditions are to severe COVID-19 infection outcomes. [Bone marrow transplantation] |
| Q7a: Please specify how vulnerable you consider the following conditions are to severe COVID-19 infection outcomes. [Stem cell transplantation] |
| Q7a: Please specify how vulnerable you consider the following conditions are to severe COVID-19 infection outcomes. [Multi-organ transplantation] |
| Q7a: Please specify how vulnerable you consider the following conditions are to severe COVID-19 infection outcomes. [Islet transplantation] |
| Q7a: Please specify how vulnerable you consider the following conditions are to severe COVID-19 infection outcomes. [Haematological malignancies] |
| Q7a: Please specify how vulnerable you consider the following conditions are to severe COVID-19 infection outcomes. [Solid tumours] |
| Q7a: Please specify how vulnerable you consider the following conditions are to severe COVID-19 infection outcomes. [Generalised malignancies (metastasis)] |
| Q7a: Please specify how vulnerable you consider the following conditions are to severe COVID-19 infection outcomes. [Actively-treated malignancy] |
| Q7a: Please specify how vulnerable you consider the following conditions are to severe COVID-19 infection outcomes. [HIV infection all stages] |
| Q7a: Please specify how vulnerable you consider the following conditions are to severe COVID-19 infection outcomes. [Drug-managed HIV] |
| Q7a: Please specify how vulnerable you consider the following conditions are to severe COVID-19 infection outcomes. [Untreated HIV] |
| Q7a: Please specify how vulnerable you consider the following conditions are to severe COVID-19 infection outcomes. [AIDS] |
| Q7a: Please specify how vulnerable you consider the following conditions are to severe COVID-19 infection outcomes. [Genetic disorders affecting the immune system (e.g. SCID, complement disorder, IRAK-4, NEMO)] |
| Q7a: Please specify how vulnerable you consider the following conditions are to severe COVID-19 infection outcomes. [Down Syndrome] |
| Q7a: Please specify how vulnerable you consider the following conditions are to severe COVID-19 infection outcomes. [Autoimmune skin diseases (e.g. Psoriasis, Dermatitis herpetiformis, Vitiligo, Pemphigus, Scleroderma, Dermatomyositis, etc.)] |
| Q7a: Please specify how vulnerable you consider the following conditions are to severe COVID-19 infection outcomes. [Rheumatologic Disorders (e.g. Systemic lupus erythematosus, Rheumatoid Arthritis, Sjögren's syndrome, Ankylosing spondylitis, Gout etc.)] |
| Q7a: Please specify how vulnerable you consider the following conditions are to severe COVID-19 infection outcomes. [Gastrointestinal Autoimmune Diseases (e.g. Inflammatory Bowel Syndrome, Celiac disease, Ulcerative colitis, Autoimmune hepatitis)] |
| Q7a: Please specify how vulnerable you consider the following conditions are to severe COVID-19 infection outcomes. [Endocrine Autoimmune disorders (e.g. Type 1 diabetes mellitus, Hashimoto’s thyroiditis, Graves' disease, Addison’s disease etc.)] |
| Q7a: Please specify how vulnerable you consider the following conditions are to severe COVID-19 infection outcomes. [Neurological Autoimmune Conditions (e.g. Multiple sclerosis, Myasthenia gravis, Autoimmune encephalitis, Guillain-Barré syndrome etc.)] |
| Q7a: Please specify how vulnerable you consider the following conditions are to severe COVID-19 infection outcomes. [Hematological Autoimmune Disorders (e.g. Evan’s syndrome, Immune thrombocytopenic purpura, Antiphospholipid syndrome etc.)] |
| Q7a: Please specify how vulnerable you consider the following conditions are to severe COVID-19 infection outcomes. [Renal Autoimmune Diseases (e.g. Lupus nephritis, Glomerulonephritis, IgA nephropathy etc.)] |
| Q7a: Please specify how vulnerable you consider the following conditions are to severe COVID-19 infection outcomes. [Opthalmologic autoimmune conditions (e.g. Uveitis, Graves’ ophthalmopathy etc.)] |
| Q7a: Please specify how vulnerable you consider the following conditions are to severe COVID-19 infection outcomes. [Underlying aberrant immunity (e.g. graft-vs-host disease, graft rejection, absent or incomplete immune reconstitution, neutropenia ANC, lymphopenia ALC)] |
| Q7a: Please specify how vulnerable you consider the following conditions are to severe COVID-19 infection outcomes. [Type 2 diabetes mellitus] |
| Q7a: Please specify how vulnerable you consider the following conditions are to severe COVID-19 infection outcomes. [Asplenia (anatomic/ functional)] |
| Q7a: Please specify how vulnerable you consider the following conditions are to severe COVID-19 infection outcomes. [Aplastic anemia] |
| Q7a: Please specify how vulnerable you consider the following conditions are to severe COVID-19 infection outcomes. [Sickle cell disease] |
| Q7a: Please specify how vulnerable you consider the following conditions are to severe COVID-19 infection outcomes. [Chronic kidney disease] |
| Q7a: Please specify how vulnerable you consider the following conditions are to severe COVID-19 infection outcomes. [Nephrotic syndrome] |
| Q7a: Please specify how vulnerable you consider the following conditions are to severe COVID-19 infection outcomes. [Dialysis] |
| Q7a: Please specify how vulnerable you consider the following conditions are to severe COVID-19 infection outcomes. [Cerebrospinal fluid leak] |
| Q7a: Please specify how vulnerable you consider the following conditions are to severe COVID-19 infection outcomes. [Cochlear implant] |
| Q7a: Please specify how vulnerable you consider the following conditions are to severe COVID-19 infection outcomes. [Burn injuries] |
| Q7a: Please specify how vulnerable you consider the following conditions are to severe COVID-19 infection outcomes. [Pregnancy] |
| Q7a: Please specify how vulnerable you consider the following conditions are to severe COVID-19 infection outcomes. [Chronic stress] |
| Q7a: Please specify how vulnerable you consider the following conditions are to severe COVID-19 infection outcomes. [Sleep deprivation] |
| Q7a: Please specify how vulnerable you consider the following conditions are to severe COVID-19 infection outcomes. [Malnutrition] |
| Q7a: Please specify how vulnerable you consider the following conditions are to severe COVID-19 infection outcomes. [Stunting] |
| Q7a: Please specify how vulnerable you consider the following conditions are to severe COVID-19 infection outcomes. [Anorexia Nervosa] |
| Q7b: Please justify your answers to Q7a, specifying if there were any areas where you were uncertain and why. |

**Round 2, n = 60**

- *Evaluation of first draft of phenotypes (Risk-Categorised and Risk-Stratified).*
- *Appraisal of candidate diagnoses’ (including those recommended by panellists in previous round) appropriateness for inclusion in a digital phenotype for ‘adult immunosuppression’, higher versus lower risk status for COVID-19 severe outcomes and excess vulnerability compared to the immunocompetent.*
- *Assessment of a range of clinical consensus statements.*

| Please enter your Panellist ID number. |
| --- |
| Q1a: To what extent do you agree with the following statements? [Immunosuppression is poorly defined] |
| Q1a: To what extent do you agree with the following statements? [Our current clinical definition for immunosuppression is too expansive] |
| Q1a: To what extent do you agree with the following statements? [Our current clinical definition for immunosuppression is not expansive enough] |
| Q1a: To what extent do you agree with the following statements? [The terms 'immunosuppression' and 'immunocompromised' can be used interchangeably] |
| Q1a: To what extent do you agree with the following statements? [The needs of the immunosuppressed are sufficiently prioritised in times of public health emergency] |
| Q1a: To what extent do you agree with the following statements? [COVID-19 no longer poses a real risk to the immunosuppressed] |
| Q1a: To what extent do you agree with the following statements? [Vulnerability to COVID-19 is similar for all immunosuppressed subgroups] |
| Q1a: To what extent do you agree with the following statements? [It is easy to pick out the immunosuppressed subgroups at most risk for COVID-19] |
| Q1a: To what extent do you agree with the following statements? [The immunosuppressed subgroups that are at the most risk for COVID-19 are the same subgroups at most risk for other infectious diseases (RSV, influenza, bacterial pneumonia etc.)] |
| Q1a: To what extent do you agree with the following statements? [COVID care for the immunosuppressed – including vaccination and the release of antivirals and other resources – is overly one size fits all] |
| Q1b: Please justify your answers to Q1a, specifying if there were any areas where you were uncertain and why. |
| Q2a: The following conditions are explicitly referenced in the UK characterisation of immunosuppression. In your expert opinion, how appropriate is their inclusion? [Solid organ transplantation] |
| Q2a: The following conditions are explicitly referenced in the UK characterisation of immunosuppression. In your expert opinion, how appropriate is their inclusion? [Bone marrow transplantation] |
| Q2a: The following conditions are explicitly referenced in the UK characterisation of immunosuppression. In your expert opinion, how appropriate is their inclusion? [Stem cell transplantation] |
| Q2a: The following conditions are explicitly referenced in the UK characterisation of immunosuppression. In your expert opinion, how appropriate is their inclusion? [Haematological malignancies] |
| Q2a: The following conditions are explicitly referenced in the UK characterisation of immunosuppression. In your expert opinion, how appropriate is their inclusion? [HIV infection at all stages] |
| Q2a: The following conditions are explicitly referenced in the UK characterisation of immunosuppression. In your expert opinion, how appropriate is their inclusion? [Genetic disorders affecting the immune system (e.g. SCID, complement disorder, IRAK-4, NEMO)] |
| Q2a: The following conditions are explicitly referenced in the UK characterisation of immunosuppression. In your expert opinion, how appropriate is their inclusion? [Systemic lupus erythematosus] |
| Q2a: The following conditions are explicitly referenced in the UK characterisation of immunosuppression. In your expert opinion, how appropriate is their inclusion? [Rheumatoid arthritis] |
| Q2a: The following conditions are explicitly referenced in the UK characterisation of immunosuppression. In your expert opinion, how appropriate is their inclusion? [Inflammatory bowel disease] |
| Q2a: The following conditions are explicitly referenced in the UK characterisation of immunosuppression. In your expert opinion, how appropriate is their inclusion? [Scleroderma] |
| Q2a: The following conditions are explicitly referenced in the UK characterisation of immunosuppression. In your expert opinion, how appropriate is their inclusion? [Psoriasis] |
| Q2b: Please justify your answers to Q2a, specifying if there were any areas where you were uncertain and why. |
| Q3a: The following conditions are not explicitly referenced in the UK characterisation of immunosuppression but appear in comparable national and international resources. In your expert opinion, how appropriate would it be to include them going forwards? [Multi-organ transplantation] |
| Q3a: The following conditions are not explicitly referenced in the UK characterisation of immunosuppression but appear in comparable national and international resources. In your expert opinion, how appropriate would it be to include them going forwards? [Islet transplantation] |
| Q3a: The following conditions are not explicitly referenced in the UK characterisation of immunosuppression but appear in comparable national and international resources. In your expert opinion, how appropriate would it be to include them going forwards? [Solid tumours] |
| Q3a: The following conditions are not explicitly referenced in the UK characterisation of immunosuppression but appear in comparable national and international resources. In your expert opinion, how appropriate would it be to include them going forwards? [Generalised malignancies (metastasis)] |
| Q3a: The following conditions are not explicitly referenced in the UK characterisation of immunosuppression but appear in comparable national and international resources. In your expert opinion, how appropriate would it be to include them going forwards? [Drug-managed HIV] |
| Q3a: The following conditions are not explicitly referenced in the UK characterisation of immunosuppression but appear in comparable national and international resources. In your expert opinion, how appropriate would it be to include them going forwards? [Untreated HIV] |
| Q3a: The following conditions are not explicitly referenced in the UK characterisation of immunosuppression but appear in comparable national and international resources. In your expert opinion, how appropriate would it be to include them going forwards? [AIDS] |
| Q3a: The following conditions are not explicitly referenced in the UK characterisation of immunosuppression but appear in comparable national and international resources. In your expert opinion, how appropriate would it be to include them going forwards? [Down Syndrome] |
| Q3a: The following conditions are not explicitly referenced in the UK characterisation of immunosuppression but appear in comparable national and international resources. In your expert opinion, how appropriate would it be to include them going forwards? [Autoimmune skin diseases (e.g. Dermatitis herpetiformis, Vitiligo, Pemphigus, Dermatomyositis, etc.)] |
| Q3a: The following conditions are not explicitly referenced in the UK characterisation of immunosuppression but appear in comparable national and international resources. In your expert opinion, how appropriate would it be to include them going forwards? [Rheumatologic Disorders (e.g. Sjögren's syndrome, Ankylosing spondylitis, Gout etc.)] |
| Q3a: The following conditions are not explicitly referenced in the UK characterisation of immunosuppression but appear in comparable national and international resources. In your expert opinion, how appropriate would it be to include them going forwards? [Gastrointestinal Autoimmune Diseases (e.g. Celiac disease, Ulcerative colitis, Autoimmune hepatitis)] |
| Q3a: The following conditions are not explicitly referenced in the UK characterisation of immunosuppression but appear in comparable national and international resources. In your expert opinion, how appropriate would it be to include them going forwards? [Endocrine Autoimmune disorders (e.g. Type 1 diabetes mellitus, Hashimoto’s thyroiditis, Graves' disease, Addison’s disease etc.)] |
| Q3a: The following conditions are not explicitly referenced in the UK characterisation of immunosuppression but appear in comparable national and international resources. In your expert opinion, how appropriate would it be to include them going forwards? [Neurological Autoimmune Conditions (e.g. Multiple sclerosis, Myasthenia gravis, Autoimmune encephalitis, Guillain-Barré syndrome etc.)] |
| Q3a: The following conditions are not explicitly referenced in the UK characterisation of immunosuppression but appear in comparable national and international resources. In your expert opinion, how appropriate would it be to include them going forwards? [Hematological Autoimmune Disorders (e.g. Evan’s syndrome, Immune thrombocytopenic purpura, Antiphospholipid syndrome etc.)] |
| Q3a: The following conditions are not explicitly referenced in the UK characterisation of immunosuppression but appear in comparable national and international resources. In your expert opinion, how appropriate would it be to include them going forwards? [Renal Autoimmune Diseases (e.g. Lupus nephritis, Glomerulonephritis, IgA nephropathy etc.)] |
| Q3a: The following conditions are not explicitly referenced in the UK characterisation of immunosuppression but appear in comparable national and international resources. In your expert opinion, how appropriate would it be to include them going forwards? [Opthalmologic autoimmune conditions (e.g. Uveitis, Graves’ ophthalmopathy etc.)] |
| Q3a: The following conditions are not explicitly referenced in the UK characterisation of immunosuppression but appear in comparable national and international resources. In your expert opinion, how appropriate would it be to include them going forwards? [Underlying aberrant immunity (e.g. graft-vs-host disease, graft rejection, absent or incomplete immune reconstitution, neutropenia ANC, lymphopenia ALC)] |
| Q3a: The following conditions are not explicitly referenced in the UK characterisation of immunosuppression but appear in comparable national and international resources. In your expert opinion, how appropriate would it be to include them going forwards? [Type 2 diabetes mellitus] |
| Q3a: The following conditions are not explicitly referenced in the UK characterisation of immunosuppression but appear in comparable national and international resources. In your expert opinion, how appropriate would it be to include them going forwards? [Asplenia (anatomic/functional)] |
| Q3a: The following conditions are not explicitly referenced in the UK characterisation of immunosuppression but appear in comparable national and international resources. In your expert opinion, how appropriate would it be to include them going forwards? [Aplastic anemia] |
| Q3a: The following conditions are not explicitly referenced in the UK characterisation of immunosuppression but appear in comparable national and international resources. In your expert opinion, how appropriate would it be to include them going forwards? [Sickle cell disease] |
| Q3a: The following conditions are not explicitly referenced in the UK characterisation of immunosuppression but appear in comparable national and international resources. In your expert opinion, how appropriate would it be to include them going forwards? [Chronic kidney disease] |
| Q3a: The following conditions are not explicitly referenced in the UK characterisation of immunosuppression but appear in comparable national and international resources. In your expert opinion, how appropriate would it be to include them going forwards? [Nephrotic syndrome] |
| Q3a: The following conditions are not explicitly referenced in the UK characterisation of immunosuppression but appear in comparable national and international resources. In your expert opinion, how appropriate would it be to include them going forwards? [Dialysis] |
| Q3a: The following conditions are not explicitly referenced in the UK characterisation of immunosuppression but appear in comparable national and international resources. In your expert opinion, how appropriate would it be to include them going forwards? [Cerebrospinal fluid leak] |
| Q3a: The following conditions are not explicitly referenced in the UK characterisation of immunosuppression but appear in comparable national and international resources. In your expert opinion, how appropriate would it be to include them going forwards? [Cochlear implant] |
| Q3a: The following conditions are not explicitly referenced in the UK characterisation of immunosuppression but appear in comparable national and international resources. In your expert opinion, how appropriate would it be to include them going forwards? [Burn injuries] |
| Q3a: The following conditions are not explicitly referenced in the UK characterisation of immunosuppression but appear in comparable national and international resources. In your expert opinion, how appropriate would it be to include them going forwards? [Pregnancy] |
| Q3a: The following conditions are not explicitly referenced in the UK characterisation of immunosuppression but appear in comparable national and international resources. In your expert opinion, how appropriate would it be to include them going forwards? [Chronic stress] |
| Q3a: The following conditions are not explicitly referenced in the UK characterisation of immunosuppression but appear in comparable national and international resources. In your expert opinion, how appropriate would it be to include them going forwards? [Sleep deprivation] |
| Q3a: The following conditions are not explicitly referenced in the UK characterisation of immunosuppression but appear in comparable national and international resources. In your expert opinion, how appropriate would it be to include them going forwards? [Malnutrition] |
| Q3a: The following conditions are not explicitly referenced in the UK characterisation of immunosuppression but appear in comparable national and international resources. In your expert opinion, how appropriate would it be to include them going forwards? [Stunting] |
| Q3a: The following conditions are not explicitly referenced in the UK characterisation of immunosuppression but appear in comparable national and international resources. In your expert opinion, how appropriate would it be to include them going forwards? [Anorexia Nervosa] |
| Q3b: Please justify your answers to Q3a, specifying if there were any areas where you were uncertain and why. |
| Q4: Were there any conditions you were expecting to see that were not listed in either Q2 or Q3? If yes, please name them. |
| Q5a: To what extent do you agree with the following statements: Immunosuppression would not be conferred if... [The patient's cancer is in remission] |
| Q5a: To what extent do you agree with the following statements: Immunosuppression would not be conferred if... [The patient's cancer is currently untreated] |
| Q5a: To what extent do you agree with the following statements: Immunosuppression would not be conferred if... [The patient's cancer is classed as Early Stage (Stage I)] |
| Q5a: To what extent do you agree with the following statements: Immunosuppression would not be conferred if... [The patient's HIV is drug-managed] |
| Q5a: To what extent do you agree with the following statements: Immunosuppression would not be conferred if... [The patient's inflammatory disease is currently untreated] |
| Q5a: To what extent do you agree with the following statements: Immunosuppression would not be conferred if... [The patient has received immunosuppressive therapy for less than 6 months] |
| Q5a: To what extent do you agree with the following statements: Immunosuppression would not be conferred if... [The patient's immunosuppressive regimen was discontinued more than 6 months ago] |
| Q5a: To what extent do you agree with the following statements: Immunosuppression would not be conferred if... [The patient's immunosuppressive regimen was discontinued more than 1 year ago] |
| Q5a: To what extent do you agree with the following statements: Immunosuppression would not be conferred if... [The patient is on low-dose immunosuppressives (equivalent of < 20mg of prednisone per day)] |
| Q5b: Please justify your answers to Q5a, specifying if there were any areas where you were uncertain and why. |
| Q6a: Please specify whether you consider the following conditions at higher or lower risk for severe COVID-19 outcomes? [Solid organ transplantation] |
| Q6a: Please specify whether you consider the following conditions at higher or lower risk for severe COVID-19 outcomes? [Bone marrow transplantation] |
| Q6a: Please specify whether you consider the following conditions at higher or lower risk for severe COVID-19 outcomes? [Stem cell transplantation] |
| Q6a: Please specify whether you consider the following conditions at higher or lower risk for severe COVID-19 outcomes? [Multi-organ transplantation] |
| Q6a: Please specify whether you consider the following conditions at higher or lower risk for severe COVID-19 outcomes? [Islet transplantation] |
| Q6a: Please specify whether you consider the following conditions at higher or lower risk for severe COVID-19 outcomes? [Haematological malignancies] |
| Q6a: Please specify whether you consider the following conditions at higher or lower risk for severe COVID-19 outcomes? [Solid tumours] |
| Q6a: Please specify whether you consider the following conditions at higher or lower risk for severe COVID-19 outcomes? [Generalised malignancies (metastasis)] |
| Q6a: Please specify whether you consider the following conditions at higher or lower risk for severe COVID-19 outcomes? [Actively-treated malignancy] |
| Q6a: Please specify whether you consider the following conditions at higher or lower risk for severe COVID-19 outcomes? [HIV infection all stages] |
| Q6a: Please specify whether you consider the following conditions at higher or lower risk for severe COVID-19 outcomes? [Drug-managed HIV] |
| Q6a: Please specify whether you consider the following conditions at higher or lower risk for severe COVID-19 outcomes? [Untreated HIV] |
| Q6a: Please specify whether you consider the following conditions at higher or lower risk for severe COVID-19 outcomes? [AIDS] |
| Q6a: Please specify whether you consider the following conditions at higher or lower risk for severe COVID-19 outcomes? [Genetic disorders affecting the immune system (e.g. SCID, complement disorder, IRAK-4, NEMO)] |
| Q6a: Please specify whether you consider the following conditions at higher or lower risk for severe COVID-19 outcomes? [Down Syndrome] |
| Q6a: Please specify whether you consider the following conditions at higher or lower risk for severe COVID-19 outcomes? [Autoimmune skin diseases (e.g. Psoriasis, Dermatitis herpetiformis, Vitiligo, Pemphigus, Scleroderma, Dermatomyositis, etc.)] |
| Q6a: Please specify whether you consider the following conditions at higher or lower risk for severe COVID-19 outcomes? [Rheumatologic Disorders (e.g. Systemic lupus erythematosus, Rheumatoid Arthritis, Sjögren's syndrome, Ankylosing spondylitis, Gout etc.)] |
| Q6a: Please specify whether you consider the following conditions at higher or lower risk for severe COVID-19 outcomes? [Gastrointestinal Autoimmune Diseases (e.g. Inflammatory Bowel Syndrome, Celiac disease, Ulcerative colitis, Autoimmune hepatitis)] |
| Q6a: Please specify whether you consider the following conditions at higher or lower risk for severe COVID-19 outcomes? [Endocrine Autoimmune disorders (e.g. Type 1 diabetes mellitus, Hashimoto’s thyroiditis, Graves' disease, Addison’s disease etc.)] |
| Q6a: Please specify whether you consider the following conditions at higher or lower risk for severe COVID-19 outcomes? [Neurological Autoimmune Conditions (e.g. Multiple sclerosis, Myasthenia gravis, Autoimmune encephalitis, Guillain-Barré syndrome etc.)] |
| Q6a: Please specify whether you consider the following conditions at higher or lower risk for severe COVID-19 outcomes? [Hematological Autoimmune Disorders (e.g. Evan’s syndrome, Immune thrombocytopenic purpura, Antiphospholipid syndrome etc.)] |
| Q6a: Please specify whether you consider the following conditions at higher or lower risk for severe COVID-19 outcomes? [Renal Autoimmune Diseases (e.g. Lupus nephritis, Glomerulonephritis, IgA nephropathy etc.)] |
| Q6a: Please specify whether you consider the following conditions at higher or lower risk for severe COVID-19 outcomes? [Opthalmologic autoimmune conditions (e.g. Uveitis, Graves’ ophthalmopathy etc.)] |
| Q6a: Please specify whether you consider the following conditions at higher or lower risk for severe COVID-19 outcomes? [Underlying aberrant immunity (e.g. graft-vs-host disease, graft rejection, absent or incomplete immune reconstitution, neutropenia ANC, lymphopenia ALC)] |
| Q6a: Please specify whether you consider the following conditions at higher or lower risk for severe COVID-19 outcomes? [Type 2 diabetes mellitus] |
| Q6a: Please specify whether you consider the following conditions at higher or lower risk for severe COVID-19 outcomes? [Asplenia (anatomic/ functional)] |
| Q6a: Please specify whether you consider the following conditions at higher or lower risk for severe COVID-19 outcomes? [Aplastic anemia] |
| Q6a: Please specify whether you consider the following conditions at higher or lower risk for severe COVID-19 outcomes? [Sickle cell disease] |
| Q6a: Please specify whether you consider the following conditions at higher or lower risk for severe COVID-19 outcomes? [Chronic kidney disease] |
| Q6a: Please specify whether you consider the following conditions at higher or lower risk for severe COVID-19 outcomes? [Nephrotic syndrome] |
| Q6a: Please specify whether you consider the following conditions at higher or lower risk for severe COVID-19 outcomes? [Dialysis] |
| Q6a: Please specify whether you consider the following conditions at higher or lower risk for severe COVID-19 outcomes? [Cerebrospinal fluid leak] |
| Q6a: Please specify whether you consider the following conditions at higher or lower risk for severe COVID-19 outcomes? [Cochlear implant] |
| Q6a: Please specify whether you consider the following conditions at higher or lower risk for severe COVID-19 outcomes? [Burn injuries] |
| Q6a: Please specify whether you consider the following conditions at higher or lower risk for severe COVID-19 outcomes? [Pregnancy] |
| Q6a: Please specify whether you consider the following conditions at higher or lower risk for severe COVID-19 outcomes? [Chronic stress] |
| Q6a: Please specify whether you consider the following conditions at higher or lower risk for severe COVID-19 outcomes? [Sleep deprivation] |
| Q6a: Please specify whether you consider the following conditions at higher or lower risk for severe COVID-19 outcomes? [Malnutrition] |
| Q6a: Please specify whether you consider the following conditions at higher or lower risk for severe COVID-19 outcomes? [Stunting] |
| Q6a: Please specify whether you consider the following conditions at higher or lower risk for severe COVID-19 outcomes? [Anorexia Nervosa] |
| Q6b: Please justify your answers to Q6a, specifying if there were any areas where you were uncertain and why. |
| Q7a: Please specify how vulnerable you consider the following conditions are to severe COVID-19 infection outcomes. [Solid organ transplantation] |
| Q7a: Please specify how vulnerable you consider the following conditions are to severe COVID-19 infection outcomes. [Bone marrow transplantation] |
| Q7a: Please specify how vulnerable you consider the following conditions are to severe COVID-19 infection outcomes. [Stem cell transplantation] |
| Q7a: Please specify how vulnerable you consider the following conditions are to severe COVID-19 infection outcomes. [Multi-organ transplantation] |
| Q7a: Please specify how vulnerable you consider the following conditions are to severe COVID-19 infection outcomes. [Islet transplantation] |
| Q7a: Please specify how vulnerable you consider the following conditions are to severe COVID-19 infection outcomes. [Haematological malignancies] |
| Q7a: Please specify how vulnerable you consider the following conditions are to severe COVID-19 infection outcomes. [Solid tumours] |
| Q7a: Please specify how vulnerable you consider the following conditions are to severe COVID-19 infection outcomes. [Generalised malignancies (metastasis)] |
| Q7a: Please specify how vulnerable you consider the following conditions are to severe COVID-19 infection outcomes. [Actively-treated malignancy] |
| Q7a: Please specify how vulnerable you consider the following conditions are to severe COVID-19 infection outcomes. [HIV infection all stages] |
| Q7a: Please specify how vulnerable you consider the following conditions are to severe COVID-19 infection outcomes. [Drug-managed HIV] |
| Q7a: Please specify how vulnerable you consider the following conditions are to severe COVID-19 infection outcomes. [Untreated HIV] |
| Q7a: Please specify how vulnerable you consider the following conditions are to severe COVID-19 infection outcomes. [AIDS] |
| Q7a: Please specify how vulnerable you consider the following conditions are to severe COVID-19 infection outcomes. [Genetic disorders affecting the immune system (e.g. SCID, complement disorder, IRAK-4, NEMO)] |
| Q7a: Please specify how vulnerable you consider the following conditions are to severe COVID-19 infection outcomes. [Down Syndrome] |
| Q7a: Please specify how vulnerable you consider the following conditions are to severe COVID-19 infection outcomes. [Autoimmune skin diseases (e.g. Psoriasis, Dermatitis herpetiformis, Vitiligo, Pemphigus, Scleroderma, Dermatomyositis, etc.)] |
| Q7a: Please specify how vulnerable you consider the following conditions are to severe COVID-19 infection outcomes. [Rheumatologic Disorders (e.g. Systemic lupus erythematosus, Rheumatoid Arthritis, Sjögren's syndrome, Ankylosing spondylitis, Gout etc.)] |
| Q7a: Please specify how vulnerable you consider the following conditions are to severe COVID-19 infection outcomes. [Gastrointestinal Autoimmune Diseases (e.g. Inflammatory Bowel Syndrome, Celiac disease, Ulcerative colitis, Autoimmune hepatitis)] |
| Q7a: Please specify how vulnerable you consider the following conditions are to severe COVID-19 infection outcomes. [Endocrine Autoimmune disorders (e.g. Type 1 diabetes mellitus, Hashimoto’s thyroiditis, Graves' disease, Addison’s disease etc.)] |
| Q7a: Please specify how vulnerable you consider the following conditions are to severe COVID-19 infection outcomes. [Neurological Autoimmune Conditions (e.g. Multiple sclerosis, Myasthenia gravis, Autoimmune encephalitis, Guillain-Barré syndrome etc.)] |
| Q7a: Please specify how vulnerable you consider the following conditions are to severe COVID-19 infection outcomes. [Hematological Autoimmune Disorders (e.g. Evan’s syndrome, Immune thrombocytopenic purpura, Antiphospholipid syndrome etc.)] |
| Q7a: Please specify how vulnerable you consider the following conditions are to severe COVID-19 infection outcomes. [Renal Autoimmune Diseases (e.g. Lupus nephritis, Glomerulonephritis, IgA nephropathy etc.)] |
| Q7a: Please specify how vulnerable you consider the following conditions are to severe COVID-19 infection outcomes. [Opthalmologic autoimmune conditions (e.g. Uveitis, Graves’ ophthalmopathy etc.)] |
| Q7a: Please specify how vulnerable you consider the following conditions are to severe COVID-19 infection outcomes. [Underlying aberrant immunity (e.g. graft-vs-host disease, graft rejection, absent or incomplete immune reconstitution, neutropenia ANC, lymphopenia ALC)] |
| Q7a: Please specify how vulnerable you consider the following conditions are to severe COVID-19 infection outcomes. [Type 2 diabetes mellitus] |
| Q7a: Please specify how vulnerable you consider the following conditions are to severe COVID-19 infection outcomes. [Asplenia (anatomic/ functional)] |
| Q7a: Please specify how vulnerable you consider the following conditions are to severe COVID-19 infection outcomes. [Aplastic anemia] |
| Q7a: Please specify how vulnerable you consider the following conditions are to severe COVID-19 infection outcomes. [Sickle cell disease] |
| Q7a: Please specify how vulnerable you consider the following conditions are to severe COVID-19 infection outcomes. [Chronic kidney disease] |
| Q7a: Please specify how vulnerable you consider the following conditions are to severe COVID-19 infection outcomes. [Nephrotic syndrome] |
| Q7a: Please specify how vulnerable you consider the following conditions are to severe COVID-19 infection outcomes. [Dialysis] |
| Q7a: Please specify how vulnerable you consider the following conditions are to severe COVID-19 infection outcomes. [Cerebrospinal fluid leak] |
| Q7a: Please specify how vulnerable you consider the following conditions are to severe COVID-19 infection outcomes. [Cochlear implant] |
| Q7a: Please specify how vulnerable you consider the following conditions are to severe COVID-19 infection outcomes. [Burn injuries] |
| Q7a: Please specify how vulnerable you consider the following conditions are to severe COVID-19 infection outcomes. [Pregnancy] |
| Q7a: Please specify how vulnerable you consider the following conditions are to severe COVID-19 infection outcomes. [Chronic stress] |
| Q7a: Please specify how vulnerable you consider the following conditions are to severe COVID-19 infection outcomes. [Sleep deprivation] |
| Q7a: Please specify how vulnerable you consider the following conditions are to severe COVID-19 infection outcomes. [Malnutrition] |
| Q7a: Please specify how vulnerable you consider the following conditions are to severe COVID-19 infection outcomes. [Stunting] |
| Q7a: Please specify how vulnerable you consider the following conditions are to severe COVID-19 infection outcomes. [Anorexia Nervosa] |
| Q7b: Please justify your answers to Q7a, specifying if there were any areas where you were uncertain and why. |

**Data Collection prior to Final Discussion, n = 29**

- *For those set to attend final discussion groups in-person; 1 panellist that participated in final discussion did not return this intake form.*
- *Evaluation of second drafts of phenotypes (Risk-Categorised and Risk-Stratified).*
- *Assessment of clinical consensus statements panel had yet to reach agreement on.*

| Please enter your Panellist ID number. |
| --- |
| 1a. To what extent to you agree with the following descriptions of the draft COVID-19 Risk Categorised Phenotype for Adult Immunosuppression (Phenotype 1)? For your convenience, please reference the the the draft phenotypes document linked while answering this question. [This version is an improvement to the last draft] |
| 1a. To what extent to you agree with the following descriptions of the draft COVID-19 Risk Categorised Phenotype for Adult Immunosuppression (Phenotype 1)? For your convenience, please reference the the the draft phenotypes document linked while answering this question. [I prefer the three-part categorisation to the previous two-part categorisation] |
| 1a. To what extent to you agree with the following descriptions of the draft COVID-19 Risk Categorised Phenotype for Adult Immunosuppression (Phenotype 1)? For your convenience, please reference the the the draft phenotypes document linked while answering this question. [Drug-managed HIV is a fair active control group for this phenotype ] |
| 1a. To what extent to you agree with the following descriptions of the draft COVID-19 Risk Categorised Phenotype for Adult Immunosuppression (Phenotype 1)? For your convenience, please reference the the the draft phenotypes document linked while answering this question. [It would be useful to compare phenotype COVID-19 outcomes to those of this active control group ] |
| 1a. To what extent to you agree with the following descriptions of the draft COVID-19 Risk Categorised Phenotype for Adult Immunosuppression (Phenotype 1)? For your convenience, please reference the the the draft phenotypes document linked while answering this question. [This phenotype is an accurate representation of results from the last round] |
| 1a. To what extent to you agree with the following descriptions of the draft COVID-19 Risk Categorised Phenotype for Adult Immunosuppression (Phenotype 1)? For your convenience, please reference the the the draft phenotypes document linked while answering this question. [In reference to the immunosuppressed spectrum, this phenotype correctly allocates conditions into High Risk vs Moderate Risk vs Low Risk for severe COVID-19 outcomes ] |
| 1a. To what extent to you agree with the following descriptions of the draft COVID-19 Risk Categorised Phenotype for Adult Immunosuppression (Phenotype 1)? For your convenience, please reference the the the draft phenotypes document linked while answering this question. [This phenotype represents a step forward for subdividing immunosuppressed COVID infection outcomes] |
| 1a. To what extent to you agree with the following descriptions of the draft COVID-19 Risk Categorised Phenotype for Adult Immunosuppression (Phenotype 1)? For your convenience, please reference the the the draft phenotypes document linked while answering this question. [It would be useful to apply this phenotype to subdivide immunosuppressed outcomes for other diseases (seasonal influenza, pneumonia, RSV etc.)] |
| Q1b: Please justify your answers to Q1a, specifying if there were any areas where you were uncertain and why. |
| 2a. To what extent to you agree with the following descriptions of the draft COVID-19 Risk Stratified Phenotype for Adult Immunosuppression (Phenotype 2)? For your convenience, please reference the the the draft phenotypes document linked while answering this question. [This version is an improvement to the last draft] |
| 2a. To what extent to you agree with the following descriptions of the draft COVID-19 Risk Stratified Phenotype for Adult Immunosuppression (Phenotype 2)? For your convenience, please reference the the the draft phenotypes document linked while answering this question. [This phenotype is an accurate representation of results from the last round] |
| 2a. To what extent to you agree with the following descriptions of the draft COVID-19 Risk Stratified Phenotype for Adult Immunosuppression (Phenotype 2)? For your convenience, please reference the the the draft phenotypes document linked while answering this question. [Drug-managed HIV is a fair active control group for this phenotype] |
| 2a. To what extent to you agree with the following descriptions of the draft COVID-19 Risk Stratified Phenotype for Adult Immunosuppression (Phenotype 2)? For your convenience, please reference the the the draft phenotypes document linked while answering this question. [It would be useful to compare phenotype COVID-19 outcomes to those of this active control group] |
| 2a. To what extent to you agree with the following descriptions of the draft COVID-19 Risk Stratified Phenotype for Adult Immunosuppression (Phenotype 2)? For your convenience, please reference the the the draft phenotypes document linked while answering this question. [The levels of this phenotype are presented in the correct order (descending levels of risk for severe COVID-19 outcomes)] |
| 2a. To what extent to you agree with the following descriptions of the draft COVID-19 Risk Stratified Phenotype for Adult Immunosuppression (Phenotype 2)? For your convenience, please reference the the the draft phenotypes document linked while answering this question. [Conditions are correctly allocated to phenotype levels] |
| 2a. To what extent to you agree with the following descriptions of the draft COVID-19 Risk Stratified Phenotype for Adult Immunosuppression (Phenotype 2)? For your convenience, please reference the the the draft phenotypes document linked while answering this question. [This phenotype represents a step forward for subdividing immunosuppressed COVID-19 infection outcomes] |
| 2a. To what extent to you agree with the following descriptions of the draft COVID-19 Risk Stratified Phenotype for Adult Immunosuppression (Phenotype 2)? For your convenience, please reference the the the draft phenotypes document linked while answering this question. [It would be useful to apply this phenotype to subdivide immunosuppressed outcomes for other diseases (seasonal influenza, pneumonia, RSV etc.)] |
| Q2b: Please justify your answers to Q2a, specifying if there were any areas where you were uncertain and why. |
| Q3a: To what extent do you agree with the following statements? [Our current clinical definition for immunosuppression is too expansive] |
| Q3a: To what extent do you agree with the following statements? [Our current clinical definition for immunosuppression is not expansive enough] |
| Q3a: To what extent do you agree with the following statements? [The terms 'immunosuppression' and 'immunocompromised' can be used interchangeably] |
| Q3a: To what extent do you agree with the following statements? [The needs of the immunosuppressed are sufficiently prioritised in times of public health emergency] |
| Q3a: To what extent do you agree with the following statements? [It is easy to pick out the immunosuppressed subgroups at most risk for COVID-19] |
| Q3a: To what extent do you agree with the following statements? [The immunosuppressed subgroups that are at the most risk for COVID-19 are the same subgroups at most risk for other infectious diseases (RSV, influenza, bacterial pneumonia etc.)] |
| Q3a: To what extent do you agree with the following statements? [COVID care for the immunosuppressed – including vaccination and the release of antivirals and other resources – is overly one size fits all] |
| 3b. Please justify your answers to Q3a, specifying if there were any areas where you were uncertain and why. |
| Q4a: To what extent do you agree with the following statements: 'A patient would not be considered immunosuppressed if...' [The patient's cancer is in remission] |
| Q4a: To what extent do you agree with the following statements: 'A patient would not be considered immunosuppressed if...' [The patient's cancer is current untreated] |
| Q4a: To what extent do you agree with the following statements: 'A patient would not be considered immunosuppressed if...' [The patient's cancer is classed as early stage (I)] |
| Q4a: To what extent do you agree with the following statements: 'A patient would not be considered immunosuppressed if...' [The patient's inflammatory disease is currently untreated] |
| Q4a: To what extent do you agree with the following statements: 'A patient would not be considered immunosuppressed if...' [The patient's immunosuppressive regimen was discontinued more than 6 months ago] |
| Q4a: To what extent do you agree with the following statements: 'A patient would not be considered immunosuppressed if...' [The patient's immunosuppressive regimen was discontinued more than 12 months ago] |
| Q4a: To what extent do you agree with the following statements: 'A patient would not be considered immunosuppressed if...' [The patient is on low-dose immunosuppressives (equivalent of ≤20mg of prednisone per day)] |
| Q4a: To what extent do you agree with the following statements: 'A patient would not be considered immunosuppressed if...' [The patient is on very low-dose immunosuppressives (equivalent of ≤10mg of prednisone per day)] |
| Q4b: Please justify your answers to Q4a, specifying if there were any areas where you were uncertain and why. |

**Written participation in Final Discussion Group, n = 10**

- *For those unable to join final discussion groups in-person.*
- *Evaluation of second drafts of phenotypes (Risk-Categorised and Risk-Stratified).*
- *Assessment of clinical consensus statements panel had yet to reach agreement on.*
- *Feedback on strengths and weaknesses of study.*
- *Recommendations of possible topics affecting immunosuppressed patient care for DESTINIES panel to build consensus on in future.*

| Please enter your Panellist ID number. |
| --- |
| 1a. To what extent to you agree with the following descriptions of the draft COVID-19 Risk Categorised Phenotype for Adult Immunosuppression (Phenotype 1)? For your convenience, please reference the the the draft phenotypes document linked while answering this question. [This version is an improvement to the last draft] |
| 1a. To what extent to you agree with the following descriptions of the draft COVID-19 Risk Categorised Phenotype for Adult Immunosuppression (Phenotype 1)? For your convenience, please reference the the the draft phenotypes document linked while answering this question. [I prefer the three-part categorisation to the previous two-part categorisation] |
| 1a. To what extent to you agree with the following descriptions of the draft COVID-19 Risk Categorised Phenotype for Adult Immunosuppression (Phenotype 1)? For your convenience, please reference the the the draft phenotypes document linked while answering this question. [Drug-managed HIV is a fair active control group for this phenotype ] |
| 1a. To what extent to you agree with the following descriptions of the draft COVID-19 Risk Categorised Phenotype for Adult Immunosuppression (Phenotype 1)? For your convenience, please reference the the the draft phenotypes document linked while answering this question. [It would be useful to compare phenotype COVID-19 outcomes to those of this active control group ] |
| 1a. To what extent to you agree with the following descriptions of the draft COVID-19 Risk Categorised Phenotype for Adult Immunosuppression (Phenotype 1)? For your convenience, please reference the the the draft phenotypes document linked while answering this question. [This phenotype is an accurate representation of results from the last round] |
| 1a. To what extent to you agree with the following descriptions of the draft COVID-19 Risk Categorised Phenotype for Adult Immunosuppression (Phenotype 1)? For your convenience, please reference the the the draft phenotypes document linked while answering this question. [In reference to the immunosuppressed spectrum, this phenotype correctly allocates conditions into High Risk vs Moderate Risk vs Low Risk for severe COVID-19 outcomes ] |
| 1a. To what extent to you agree with the following descriptions of the draft COVID-19 Risk Categorised Phenotype for Adult Immunosuppression (Phenotype 1)? For your convenience, please reference the the the draft phenotypes document linked while answering this question. [This phenotype represents a step forward for subdividing immunosuppressed COVID infection outcomes] |
| 1a. To what extent to you agree with the following descriptions of the draft COVID-19 Risk Categorised Phenotype for Adult Immunosuppression (Phenotype 1)? For your convenience, please reference the the the draft phenotypes document linked while answering this question. [It would be useful to apply this phenotype to subdivide immunosuppressed outcomes for other diseases (seasonal influenza, pneumonia, RSV etc.)] |
| Q1b: Please justify your answers to Q1a, specifying if there were any areas where you were uncertain and why. |
| 2a. To what extent to you agree with the following descriptions of the draft COVID-19 Risk Stratified Phenotype for Adult Immunosuppression (Phenotype 2)? For your convenience, please reference the the the draft phenotypes document linked while answering this question. [This version is an improvement to the last draft] |
| 2a. To what extent to you agree with the following descriptions of the draft COVID-19 Risk Stratified Phenotype for Adult Immunosuppression (Phenotype 2)? For your convenience, please reference the the the draft phenotypes document linked while answering this question. [This phenotype is an accurate representation of results from the last round] |
| 2a. To what extent to you agree with the following descriptions of the draft COVID-19 Risk Stratified Phenotype for Adult Immunosuppression (Phenotype 2)? For your convenience, please reference the the the draft phenotypes document linked while answering this question. [Drug-managed HIV is a fair active control group for this phenotype] |
| 2a. To what extent to you agree with the following descriptions of the draft COVID-19 Risk Stratified Phenotype for Adult Immunosuppression (Phenotype 2)? For your convenience, please reference the the the draft phenotypes document linked while answering this question. [It would be useful to compare phenotype COVID-19 outcomes to those of this active control group] |
| 2a. To what extent to you agree with the following descriptions of the draft COVID-19 Risk Stratified Phenotype for Adult Immunosuppression (Phenotype 2)? For your convenience, please reference the the the draft phenotypes document linked while answering this question. [The levels of this phenotype are presented in the correct order (descending levels of risk for severe COVID-19 outcomes)] |
| 2a. To what extent to you agree with the following descriptions of the draft COVID-19 Risk Stratified Phenotype for Adult Immunosuppression (Phenotype 2)? For your convenience, please reference the the the draft phenotypes document linked while answering this question. [Conditions are correctly allocated to phenotype levels] |
| 2a. To what extent to you agree with the following descriptions of the draft COVID-19 Risk Stratified Phenotype for Adult Immunosuppression (Phenotype 2)? For your convenience, please reference the the the draft phenotypes document linked while answering this question. [This phenotype represents a step forward for subdividing immunosuppressed COVID-19 infection outcomes] |
| 2a. To what extent to you agree with the following descriptions of the draft COVID-19 Risk Stratified Phenotype for Adult Immunosuppression (Phenotype 2)? For your convenience, please reference the the the draft phenotypes document linked while answering this question. [It would be useful to apply this phenotype to subdivide immunosuppressed outcomes for other diseases (seasonal influenza, pneumonia, RSV etc.)] |
| Q2b: Please justify your answers to Q2a, specifying if there were any areas where you were uncertain and why. |
| Q3a: To what extent do you agree with the following statements? [Our current clinical definition for immunosuppression is too expansive] |
| Q3a: To what extent do you agree with the following statements? [Our current clinical definition for immunosuppression is not expansive enough] |
| Q3a: To what extent do you agree with the following statements? [The terms 'immunosuppression' and 'immunocompromised' can be used interchangeably] |
| Q3a: To what extent do you agree with the following statements? [The needs of the immunosuppressed are sufficiently prioritised in times of public health emergency] |
| Q3a: To what extent do you agree with the following statements? [It is easy to pick out the immunosuppressed subgroups at most risk for COVID-19] |
| Q3a: To what extent do you agree with the following statements? [The immunosuppressed subgroups that are at the most risk for COVID-19 are the same subgroups at most risk for other infectious diseases (RSV, influenza, bacterial pneumonia etc.)] |
| Q3a: To what extent do you agree with the following statements? [COVID care for the immunosuppressed – including vaccination and the release of antivirals and other resources – is overly one size fits all] |
| 3b. Please justify your answers to Q3a, specifying if there were any areas where you were uncertain and why. |
| Q4a: To what extent do you agree with the following statements: 'A patient would not be considered immunosuppressed if...' [The patient's cancer is in remission] |
| Q4a: To what extent do you agree with the following statements: 'A patient would not be considered immunosuppressed if...' [The patient's cancer is current untreated] |
| Q4a: To what extent do you agree with the following statements: 'A patient would not be considered immunosuppressed if...' [The patient's cancer is classed as early stage (I)] |
| Q4a: To what extent do you agree with the following statements: 'A patient would not be considered immunosuppressed if...' [The patient's inflammatory disease is currently untreated] |
| Q4a: To what extent do you agree with the following statements: 'A patient would not be considered immunosuppressed if...' [The patient's immunosuppressive regimen was discontinued more than 6 months ago] |
| Q4a: To what extent do you agree with the following statements: 'A patient would not be considered immunosuppressed if...' [The patient's immunosuppressive regimen was discontinued more than 12 months ago] |
| Q4a: To what extent do you agree with the following statements: 'A patient would not be considered immunosuppressed if...' [The patient is on low-dose immunosuppressives (equivalent of ≤20mg of prednisone per day)] |
| Q4a: To what extent do you agree with the following statements: 'A patient would not be considered immunosuppressed if...' [The patient is on very low-dose immunosuppressives (equivalent of ≤10mg of prednisone per day)] |
| Q4b: Please justify your answers to Q4a, specifying if there were any areas where you were uncertain and why. |
| Q5a: To what extent do you agree with the following statements? [I have enjoyed participating in the DESTINIES Study] |
| Q5a: To what extent do you agree with the following statements? [I would recommend that members of my professional network consider joining the DESTINIES Consortium to conduct further consensus building exercises on issues affecting immunosuppressed patients] |
| Q5a: To what extent do you agree with the following statements? [I am looking forward to seeing the insights of this exercise implemented in real-world research and policymaking] |
| Q5a: To what extent do you agree with the following statements? [I would be willing to contribute to additional consensus building exercises as a DESTINIES Consortium member] |
| Q5a: To what extent do you agree with the following statements? [I would be willing to assess whether the outputs of these digital phenotypes in real-world data are clinically meaningful] |
| Q5b: Please justify your answers to 5a, specifying if there were any areas where you were uncertain and why. |
| Q6: List the strengths of this exercise as you see them; please provide as much detail as possible. |
| Q7: List the limitations of this exercise as you see them; please provide as much detail as possible. |
| Q8: Are there any other issues affecting immunosuppressed patients that would benefit from a similar consensus building exercise? If yes, please list them. |

**Final ratification of DESTINIES phenotype, n = 33**

- *Evaluation of DESTINIES phenotype.*
- *Feedback on strengths and weaknesses of study.*
- *Recommendations of possible topics affecting immunosuppressed patient care for DESTINIES panel to build consensus on in future.*

| Please enter your Panellist ID number. |
| --- |
| 1a. To what extent do you agree with the following statements regarding the DESTINIES phenotype? You can find this phenotype on the The final phenotype document; it is provided under the title 'The DESTINIES Phenotype: ‘Adult immunosuppression, classified by COVID-19 vulnerability’. [I am happy with this phenotype being trialled within real-world data flows] |
| 1a. To what extent do you agree with the following statements regarding the DESTINIES phenotype? You can find this phenotype on the The final phenotype document; it is provided under the title 'The DESTINIES Phenotype: ‘Adult immunosuppression, classified by COVID-19 vulnerability’. [This phenotype is an accurate representation of results from the last round] |
| 1a. To what extent do you agree with the following statements regarding the DESTINIES phenotype? You can find this phenotype on the The final phenotype document; it is provided under the title 'The DESTINIES Phenotype: ‘Adult immunosuppression, classified by COVID-19 vulnerability’. [This version is an improvement to the last draft] |
| 1a. To what extent do you agree with the following statements regarding the DESTINIES phenotype? You can find this phenotype on the The final phenotype document; it is provided under the title 'The DESTINIES Phenotype: ‘Adult immunosuppression, classified by COVID-19 vulnerability’. [I prefer the banded version of this phenotype] |
| 1a. To what extent do you agree with the following statements regarding the DESTINIES phenotype? You can find this phenotype on the The final phenotype document; it is provided under the title 'The DESTINIES Phenotype: ‘Adult immunosuppression, classified by COVID-19 vulnerability’. [The risk-banding of this phenotype includes the correct levels ] |
| 1a. To what extent do you agree with the following statements regarding the DESTINIES phenotype? You can find this phenotype on the The final phenotype document; it is provided under the title 'The DESTINIES Phenotype: ‘Adult immunosuppression, classified by COVID-19 vulnerability’. [The levels of this phenotype are presented in the correct order (descending levels of risk for severe COVID-19 outcomes)] |
| 1a. To what extent do you agree with the following statements regarding the DESTINIES phenotype? You can find this phenotype on the The final phenotype document; it is provided under the title 'The DESTINIES Phenotype: ‘Adult immunosuppression, classified by COVID-19 vulnerability’. [Conditions are correctly allocated to phenotype levels] |
| 1a. To what extent do you agree with the following statements regarding the DESTINIES phenotype? You can find this phenotype on the The final phenotype document; it is provided under the title 'The DESTINIES Phenotype: ‘Adult immunosuppression, classified by COVID-19 vulnerability’. [Drug-managed HIV is a fair internal reference group for this phenotype ] |
| 1a. To what extent do you agree with the following statements regarding the DESTINIES phenotype? You can find this phenotype on the The final phenotype document; it is provided under the title 'The DESTINIES Phenotype: ‘Adult immunosuppression, classified by COVID-19 vulnerability’. [It would be useful to compare phenotype COVID-19 outcomes to those of the internal reference group and general population control group] |
| 1a. To what extent do you agree with the following statements regarding the DESTINIES phenotype? You can find this phenotype on the The final phenotype document; it is provided under the title 'The DESTINIES Phenotype: ‘Adult immunosuppression, classified by COVID-19 vulnerability’. [This phenotype represents a step forward for subdividing immunosuppressed COVID-19 infection outcomes in surveillance data flows] |
| 1a. To what extent do you agree with the following statements regarding the DESTINIES phenotype? You can find this phenotype on the The final phenotype document; it is provided under the title 'The DESTINIES Phenotype: ‘Adult immunosuppression, classified by COVID-19 vulnerability’. [As a hypothesis generating exercise, it would be interesting to apply this phenotype to subdivide immunosuppressed outcomes for other diseases (seasonal influenza, pneumonia, RSV etc.)] |
| Q1b: Please justify your answers to Q1a, specifying if there were any areas where you were uncertain and why. |
| Q2: List the strengths of this exercise as you see them; please provide as much detail as possible. Please write 'NA' if you addressed this question in Final Discussion. |
| Q3: List the limitations of this exercise as you see them; please provide as much detail as possible. Please write 'NA' if you addressed this question in Final Discussion. |
| Q4: Are there any other issues affecting immunosuppressed patients that would benefit from a similar consensus building exercise? If yes, please list them. Please write 'NA' if you addressed this question in Final Discussion. |

1. **List of candidate immunosuppressed terms and their sources**

| Candidate Term | Source(s) |
| --- | --- |
| Actively treated malignancy | Green Book Chapter 14a; National Institutes of Health |
| AIDS-defining illness | National Cancer Institute |
| Anorexia Nervosa | National Cancer Institute |
| Aplastic anaemia | Australian Immunisation Handbook |
| Asplenia (anatomic/ functional) | Centers for Disease Control and Prevention; Infectious Diseases Society of America; ICD-10; Immunisation Guidelines for Ireland |
| Autoimmune skin diseases (e.g. Psoriasis, Dermatitis herpetiformis, Vitiligo, Pemphigus, Scleroderma, Dermatomyositis, etc.) | Green Book Chapter 14a; John's Hopkins Medicine; USA Social Security |
| Bone marrow transplantation | Green Book Chapter 14a |
| Burn injuries | Infectious Diseases Society of America |
| Cerebrospinal fluid leak | Infectious Diseases Society of America |
| Chronic kidney disease | Centers for Disease Control and Prevention |
| Chronic Obstructive Pulmonary Disease | Panellist recommendation |
| Chronic stress | Wider determinant of immunosuppression |
| Cochlear implant | Infectious Diseases Society of America |
| Cystic Fibrosis | Panellist recommendation |
| Dialysis | Canadian Immunization Guide; Centers for Disease Control and Prevention |
| Down Syndrome | New Zealand Immunisation Handbook |
| Drug-Managed HIV | Centers for Disease Control and Prevention; Green Book Chapter 14a |
| Endocrine Autoimmune disorders (e.g. Type 1 diabetes mellitus, Hashimoto’s thyroiditis, Graves' disease, Addison’s disease etc.) | Global Autoimmune Institute |
| Gastrointestinal Autoimmune Diseases (e.g. Inflammatory Bowel Syndrome, Celiac disease, Ulcerative colitis, Autoimmune hepatitis) | Green Book Chapter 14a |
| Generalised malignancies (metastasis) | Centers for Disease Control and Prevention |
| Genetic disorders affecting the immune system (e.g. SCID, complement disorder, IRAK-4, NEMO, inherited blood disorders) | Green Book Chapter 14a; ICD-10 |
| Haematological malignancies | Green Book Chapter 14a |
| Haematological Autoimmune Disorders (e.g. Evan’s syndrome, Immune thrombocytopenic purpura, Antiphospholipid syndrome, VEXAS etc.) | Green Book Chapter 28a; Centers for Disease Control and Prevention; Global Autoimmune Institute |
| HIV infection all stages | Green Book Chapter 14a |
| Islet transplantation | National Institutes of Health |
| Liver disease (including cirrhotic) | Panellist recommendation |
| Malnutrition | National Cancer Institute |
| Multi-organ transplantation | British Columbia Centre for Disease Control; National Institutes of Health |
| Myalgic Encephalomyelitis/ Chronic Fatigue Syndrome | Panellist recommendation |
| Nephrotic syndrome | Centers for Disease Control and Prevention |
| Neurological Autoimmune Conditions (e.g. Multiple sclerosis, Myasthenia gravis, Autoimmune encephalitis, Guillain-Barré syndrome etc.) | Global Autoimmune Institute |
| Obesity | Panellist recommendation |
| Ophthalmologic autoimmune conditions (e.g. Uveitis, Graves’ ophthalmopathy etc.) | Global Autoimmune Institute |
| Pregnancy | Wider determinant of immunosuppression |
| Preterm births | Panellist recommendation |
| Renal Autoimmune Diseases (e.g. Lupus nephritis, Glomerulonephritis, IgA nephropathy etc.) | British Columbia Centre for Disease Control; Global Autoimmune Institute |
| Rheumatologic Disorders (e.g. Systemic lupus erythematosus, Rheumatoid Arthritis, Sjögren's syndrome, Idiopathic inflammatory myopathies, Ankylosing spondylitis, Vasculitides, Gout etc.) | Green Book Chapter 14a; World Health Organisation Vaccine Prioritisation Roadmap; USA Social Security |
| Sickle cell disease | Infectious Diseases Society of America |
| Skin graft recipients | Panellist recommendation |
| Sleep deprivation | Wider determinant of immunosuppression |
| Solid organ transplantation | Green Book Chapter 14a |
| Solid tumours | World Health Organisation Interim Recommendations |
| Stem cell transplantation | Green Book Chapter 14a |
| Stunting | National Cancer Institute |
| Type 2 diabetes mellitus | National Cancer Institute |
| Underlying aberrant immunity (e.g. graft-vs-host disease, graft rejection, absent or incomplete immune reconstitution, neutropenia ANC, lymphopenia ALC) | American Society of Hematology |
| Unmanaged HIV | National Institutes of Health |

**Source references:**

1. COVID-19: the green book, chapter 14a. GOV.UK. 2020. URL: https://www.gov.uk/government/publications/  covid-19-the-green-book-chapter-14a [accessed 2024-09-02]
2. Royal College of Physicians of Ireland. *Immunisation Guidelines for Ireland*. 2024, [www.rcpi.ie/Healthcare-Leadership/NIAC/Immunisation-Guidelines-for-Ireland](http://www.rcpi.ie/Healthcare-Leadership/NIAC/Immunisation-Guidelines-for-Ireland).
3. Public Health Agency of Canada. *Canadian Immunization Guide*. 2024, [www.canada.ca/en/public-health/services/canadian-immunization-guide.html](http://www.canada.ca/en/public-health/services/canadian-immunization-guide.html).
4. Health New Zealand. “Immunisation Handbook – Health New Zealand | Te Whatu Ora.” *Www.tewhatuora.govt.nz*, 2024, [www.tewhatuora.govt.nz/for-health-professionals/clinical-guidance/immunisation-handbook](http://www.tewhatuora.govt.nz/for-health-professionals/clinical-guidance/immunisation-handbook).
5. Centers for Disease Control and Prevention (2022). *Clinical Guidance for COVID-19 Vaccination | CDC*. [online] www.cdc.gov. Available at: https://www.cdc.gov/vaccines/covid-19/clinical-considerations/interim-considerations-us.html.
6. National Cancer Institute. “Https://Www.cancer.gov/Publications/Dictionaries/Cancer-Terms/Def/Immunosuppressed.” *Www.cancer.gov*, 2 Feb. 2011, [www.cancer.gov/publications/dictionaries/cancer-terms/def/immunosuppressed](http://www.cancer.gov/publications/dictionaries/cancer-terms/def/immunosuppressed).
7. Australian Government Department of Health and Aged Care. *The Australian Immunisation Handbook*. 31 July 2018, [www.health.gov.au/resources/publications/the-australian-immunisation-handbook?language=en](http://www.health.gov.au/resources/publications/the-australian-immunisation-handbook?language=en).
8. Centers for Disease Control and Prevention. “ACIP Altered Immunocompetence Guidelines for Immunizations | Recommendations | CDC.” *Www.cdc.gov*, 20 Aug. 2019, [www.cdc.gov/vaccines/hcp/acip-recs/general-recs/immunocompetence.html](http://www.cdc.gov/vaccines/hcp/acip-recs/general-recs/immunocompetence.html).
9. Centers for Disease Control and Prevention. “People with Certain Medical Conditions and COVID-19 Risk Factors.” *COVID-19*, 15 July 2024, [www.cdc.gov/covid/risk-factors/index.html](http://www.cdc.gov/covid/risk-factors/index.html).
10. Rubin, Lorry G., et al. “2013 IDSA Clinical Practice Guideline for Vaccination of the Immunocompromised Host.” *Clinical Infectious Diseases*, vol. 58, no. 3, 1 Feb. 2014, pp. e44–e100, academic.oup.com/cid/article/58/3/e44/336537, <https://doi.org/10.1093/cid/cit684>.
11. American Academy of Professional Coders. “ICD-10 Code for Immunodeficiency, Unspecified- D84.9- Codify by AAPC.” *Aapc.com*, 2024, www.aapc.com/codes/icd-10-codes/D84.9. Accessed 9 Oct. 2024.
12. John Hopkins Medicine. “Disorders of the Immune System.” *John Hopkins Medicine*, The Johns Hopkins University, The Johns Hopkins Hospital, and Johns Hopkins Health System, 2019, [www.hopkinsmedicine.org/health/conditions-and-diseases/disorders-of-the-immune-system](http://www.hopkinsmedicine.org/health/conditions-and-diseases/disorders-of-the-immune-system).
13. Social Security Administration. *14.00-Immune-Adult*. 2021, [www.ssa.gov/disability/professionals/bluebook/14.00-Immune-Adult.htm](http://www.ssa.gov/disability/professionals/bluebook/14.00-Immune-Adult.htm).
14. Global Autoimmune Institute. “Autoimmune Disease List.” *Global Autoimmune Institute*, 2024, [www.autoimmuneinstitute.org/resources/autoimmune-disease-list/](http://www.autoimmuneinstitute.org/resources/autoimmune-disease-list/).
15. GOV.UK. *Shingles (Herpes Zoster): The Green Book, Chapter 28a*. 2024, [www.gov.uk/government/publications/shingles-herpes-zoster-the-green-book-chapter-28a](http://www.gov.uk/government/publications/shingles-herpes-zoster-the-green-book-chapter-28a).
16. British Columbia Centre for Disease Control. “Part 2: Immunization of Special Populations.” *Www.bccdc.ca*, 2024, [www.bccdc.ca/health-professionals/clinical-resources/communicable-disease-control-manual/immunization/immunization-of-special-populations](http://www.bccdc.ca/health-professionals/clinical-resources/communicable-disease-control-manual/immunization/immunization-of-special-populations).
17. World Health Organisation. *WHO SAGE Roadmap for Prioritizing Uses of COVID-19 Vaccines: An Approach to Optimize the Global Impact of COVID-19 Vaccines, Based on Public Health Goals, Global and National Equity, and Vaccine Access and Coverage Scenarios*. 2023, [www.who.int/publications/i/item/WHO-2019-nCoV-Vaccines-SAGE-Prioritization-2023.1](http://www.who.int/publications/i/item/WHO-2019-nCoV-Vaccines-SAGE-Prioritization-2023.1).
18. World Health Organisation. *Interim Recommendations for an Extended Primary Series with an Additional Vaccine Dose for COVID-19 Vaccination in Immunocompromised Persons*. 2021, iris.who.int/bitstream/handle/10665/347079/WHO-2019-nCoV-Vaccination-SAGE-recommendation-Immunocompromised-persons-2021.1-eng.pdf?sequence=2.
19. American Society of Hematology. *Prevention of COVID-19 in Immunocompromised Patients with Hematologic Conditions - Hematology.org*. 2020, www.hematology.org/covid-19/archived-resources-for-clinicians/prevention-of-covid-19-in-immunocompromised-patients-with-hematologic-conditions. Accessed 9 Oct. 2024.
20. National Institutes of Health. *Immunocompromised Host - MeSH - NCBI*. 2024, www.ncbi.nlm.nih.gov/mesh?Db=mesh&Cmd=DetailsSearch&Term=%22Immunocompromised+Host%22%5BMeSH+Terms%5D. Accessed 9 Oct. 2024.
21. **Characteristics of the DESTINIES Consortium**

|  |  | **n** |
| --- | --- | --- |
| **Country** | Canada | 1 |
|  | Denmark | 1 |
|  | France | 2 |
|  | Germany | 2 |
|  | Ireland | 1 |
|  | Israel | 5 |
|  | Italy | 2 |
|  | Mexico | 1 |
|  | Netherlands | 3 |
|  | Nigeria | 1 |
|  | Singapore | 1 |
|  | Spain | 1 |
|  | Sweden | 1 |
|  | Switzerland | 1 |
|  | United Kingdom | 29 |
|  | United States | 12 |
| **Gender** | Female | 28 |
|  | Male | 36 |
| **Affiliation** | CDC ACIP | 2 |
|  | CEPI SAC | 2 |
|  | EMA Vaccines Working Party | 1 |
|  | EULAR | 18 |
|  | Food and Drug Administration (FDA) Vaccine Advisory Panel | 4 |
|  | Former WHO GACVS | 1 |
|  | GIHC | 7 |
|  | JCVI | 3 |
|  | Panellist recommendation | 5 |
|  | NDM | 4 |
|  | NDPCHS | 1 |
|  | SAGE | 1 |
|  | Van Tam Advisory Group | 15 |
| **Discipline** | **Generalist** |  |
|  | Immunology | 3 |
|  | Infectious Diseases | 14 |
|  | Vaccinology | 9 |
|  | **Specialist** |  |
|  | Autoimmunity | 4 |
|  | Clinical Informatics | 3 |
|  | Dermatology | 1 |
|  | Gastroenterology | 1 |
|  | Haematology | 1 |
|  | HIV | 3 |
|  | Nephrology | 2 |
|  | Oncology | 2 |
|  | Primary Immunodeficiencies | 1 |
|  | Rheumatology | 16 |
|  | Transplantation | 4 |

1. **DESTINIES panel participation by study round**

| **Delphi Round** | **n** |
| --- | --- |
| Round 1 | 64 |
| Round 2 | 60 |
| Final Discussion | 40 |
| Panel Ratification | 34 |

1. **Interim Datasets**

**Round 1 Interim Data**

*Appropriateness for inclusion in a phenotype of ‘adult immunosuppression’ data*

| **Diagnosis** | **Not appropriate** | **%** | **Somewhat inappropriate** | **%** | **Unsure** | **%** | **Somewhat appropriate** | **%** | **Appropriate** | **%** | **SUM disagree** | **SUM agree** | **Determination** |
| --- | --- | --- | --- | --- | --- | --- | --- | --- | --- | --- | --- | --- | --- |
| Multi-organ transplantation | 0 | 0.00 | 2 | 3.13 | 1 | 1.56 | 2 | 3.13 | 59 | 92.19 | 3.13 | 95.31 | Appropriate |
| Islet transplantation | 2 | 3.13 | 1 | 1.56 | 18 | 28.13 | 17 | 26.56 | 26 | 40.63 | 4.69 | 67.19 | Contested |
| Solid tumours | 0 | 0.00 | 5 | 7.81 | 10 | 15.63 | 25 | 39.06 | 24 | 37.50 | 7.81 | 76.56 | Appropriate |
| Generalised malignancies (metastasis) | 1 | 1.56 | 4 | 6.25 | 10 | 15.63 | 18 | 28.13 | 31 | 48.44 | 7.81 | 76.56 | Appropriate |
| Drug-managed HIV | 18 | 28.13 | 12 | 18.75 | 13 | 20.31 | 14 | 21.88 | 7 | 10.94 | 46.88 | 32.81 | Contested |
| Untreated HIV | 4 | 6.25 | 1 | 1.56 | 5 | 7.81 | 14 | 21.88 | 40 | 62.50 | 7.81 | 84.38 | Appropriate |
| AIDS | 0 | 0.00 | 0 | 0.00 | 2 | 3.13 | 7 | 10.94 | 55 | 85.94 | 0.00 | 96.88 | Appropriate |
| Down Syndrome | 2 | 3.13 | 7 | 10.94 | 18 | 28.13 | 23 | 35.94 | 14 | 21.88 | 14.06 | 57.81 | Contested |
| Autoimmune skin diseases | 5 | 7.81 | 14 | 21.88 | 18 | 28.13 | 22 | 34.38 | 5 | 7.81 | 29.69 | 42.19 | Contested |
| Rheumatologic disorders | 3 | 4.69 | 11 | 17.19 | 14 | 21.88 | 28 | 43.75 | 8 | 12.50 | 21.88 | 56.25 | Contested |
| Gastrointestinal autoimmune disorders | 2 | 3.13 | 13 | 20.31 | 19 | 29.69 | 25 | 39.06 | 5 | 7.81 | 23.44 | 46.88 | Contested |
| Endocrine autoimmune disorders | 9 | 14.06 | 17 | 26.56 | 18 | 28.13 | 16 | 25.00 | 4 | 6.25 | 40.63 | 31.25 | Contested |
| Neurologic autoimmune disorders | 1 | 1.56 | 14 | 21.88 | 15 | 23.44 | 23 | 35.94 | 11 | 17.19 | 23.44 | 53.13 | Contested |
| Hematologic autoimmune disorders | 2 | 3.13 | 17 | 26.56 | 16 | 25.00 | 20 | 31.25 | 9 | 14.06 | 29.69 | 45.31 | Contested |
| Renal autoimmune disorders | 2 | 3.13 | 14 | 21.88 | 8 | 12.50 | 25 | 39.06 | 15 | 23.44 | 25.00 | 62.50 | Contested |
| Opthalmologic autoimmune disorders | 6 | 9.38 | 18 | 28.13 | 21 | 32.81 | 16 | 25.00 | 3 | 4.69 | 37.50 | 29.69 | Contested |
| Underlying aberrant immunity | 1 | 1.56 | 1 | 1.56 | 2 | 3.13 | 21 | 32.81 | 39 | 60.94 | 3.13 | 93.75 | Appropriate |
| Type 2 Diabetes | 10 | 15.63 | 17 | 26.56 | 13 | 20.31 | 19 | 29.69 | 5 | 7.81 | 42.19 | 37.50 | Contested |
| Asplenia | 6 | 9.38 | 5 | 7.81 | 8 | 12.50 | 18 | 28.13 | 27 | 42.19 | 17.19 | 70.31 | Contested |
| Aplastic anemia | 2 | 3.13 | 9 | 14.06 | 11 | 17.19 | 18 | 28.13 | 24 | 37.50 | 17.19 | 65.63 | Contested |
| Sickle cell disease | 5 | 7.81 | 13 | 20.31 | 16 | 25.00 | 15 | 23.44 | 15 | 23.44 | 28.13 | 46.88 | Contested |
| Chronic kidney disease | 4 | 6.25 | 10 | 15.63 | 5 | 7.81 | 33 | 51.56 | 12 | 18.75 | 21.88 | 70.31 | Contested |
| Nephrotic disease | 4 | 6.25 | 12 | 18.75 | 10 | 15.63 | 27 | 42.19 | 11 | 17.19 | 25.00 | 59.38 | Contested |
| Dialysis | 2 | 3.13 | 6 | 9.38 | 10 | 15.63 | 23 | 35.94 | 23 | 35.94 | 12.50 | 71.88 | Contested |
| Cerebrospinal fluid leak | 11 | 17.19 | 14 | 21.88 | 18 | 28.13 | 11 | 17.19 | 10 | 15.63 | 39.06 | 32.81 | Contested |
| Cochlear implant | 20 | 31.25 | 10 | 15.63 | 20 | 31.25 | 8 | 12.50 | 6 | 9.38 | 46.88 | 21.88 | Contested |
| Burn injuries | 13 | 20.31 | 11 | 17.19 | 12 | 18.75 | 20 | 31.25 | 8 | 12.50 | 37.50 | 43.75 | Contested |
| Pregnancy | 15 | 23.44 | 10 | 15.63 | 9 | 14.06 | 23 | 35.94 | 7 | 10.94 | 39.06 | 46.88 | Contested |
| Chronic stress | 29 | 45.31 | 19 | 29.69 | 13 | 20.31 | 3 | 4.69 | 0 | 0.00 | 75.00 | 4.69 | Inappropriate |
| Sleep deprivation | 31 | 48.44 | 17 | 26.56 | 14 | 21.88 | 2 | 3.13 | 0 | 0.00 | 75.00 | 3.13 | Inappropriate |
| Malnutrition | 10 | 15.63 | 8 | 12.50 | 19 | 29.69 | 17 | 26.56 | 10 | 15.63 | 28.13 | 42.19 | Contested |
| Stunting | 17 | 26.56 | 14 | 21.88 | 25 | 39.06 | 7 | 10.94 | 1 | 1.56 | 48.44 | 12.50 | Contested |
| Anorexia | 13 | 20.31 | 13 | 20.31 | 19 | 29.69 | 14 | 21.88 | 5 | 7.81 | 40.63 | 29.69 | Contested |
| SOT | 0 | 0.00 | 1 | 1.56 | 0 | 0.00 | 2 | 3.13 | 61 | 95.31 | 1.56 | 98.44 | Appropriate |
| Bone marrow transplantation | 0 | 0.00 | 0 | 0.00 | 1 | 1.56 | 7 | 10.94 | 56 | 87.50 | 0.00 | 98.44 | Appropriate |
| Stem cell transplantation | 0 | 0.00 | 1 | 1.56 | 0 | 0.00 | 6 | 9.38 | 57 | 89.06 | 1.56 | 98.44 | Appropriate |
| Haematological malignancies | 0 | 0.00 | 0 | 0.00 | 0 | 0.00 | 8 | 12.50 | 56 | 87.50 | 0.00 | 100.00 | Appropriate |
| HIV infection | 4 | 6.25 | 23 | 35.94 | 7 | 10.94 | 20 | 31.25 | 10 | 15.63 | 42.19 | 46.88 | Contested |
| Genetic disorders affecting the immune system | 0 | 0.00 | 0 | 0.00 | 5 | 7.81 | 7 | 10.94 | 52 | 81.25 | 0.00 | 92.19 | Appropriate |
| Lupus | 2 | 3.13 | 8 | 12.50 | 10 | 15.63 | 16 | 25.00 | 28 | 43.75 | 15.63 | 68.75 | Contested |
| Rheumatoid Arthritis | 3 | 4.69 | 7 | 10.94 | 7 | 10.94 | 25 | 39.06 | 22 | 34.38 | 15.63 | 73.44 | Contested |
| Inflammatory bowel disease | 4 | 6.25 | 10 | 15.63 | 10 | 15.63 | 27 | 42.19 | 13 | 20.31 | 21.88 | 62.50 | Contested |
| Scleroderma | 6 | 9.38 | 8 | 12.50 | 14 | 21.88 | 18 | 28.13 | 18 | 28.13 | 21.88 | 56.25 | Contested |
| Psoriasis | 8 | 12.50 | 15 | 23.44 | 18 | 28.13 | 16 | 25.00 | 7 | 10.94 | 35.94 | 35.94 | Contested |

*Clinical consensus statements - general*

| **Statement** | **Strongly disagree** | **%** | **Slightly disagree** | **%** | **Unsure** | **%** | **Slightly agree** | **%** | **Strongly agree** | **%** | **SUM disagree** | **SUM agree** | **Determination** |
| --- | --- | --- | --- | --- | --- | --- | --- | --- | --- | --- | --- | --- | --- |
| Immunosuppression is poorly defined | 3 | 4.69 | 5 | 7.81 | 0 | 0.00 | 19 | 29.69 | 37 | 57.81 | 12.50 | 87.50 | Agree |
| Current clinical definition is too expansive | 5 | 7.81 | 18 | 28.13 | 11 | 17.19 | 18 | 28.13 | 12 | 18.75 | 35.94 | 46.88 | Contested |
| Current clinical definition isn't expansive enough | 11 | 17.19 | 18 | 28.13 | 13 | 20.31 | 18 | 28.13 | 4 | 6.25 | 45.31 | 34.38 | Contested |
| Immunosuppressed and immunocompromised are exchangable terms | 19 | 29.69 | 20 | 31.25 | 4 | 6.25 | 7 | 10.94 | 14 | 21.88 | 60.94 | 32.81 | Contested |
| Immunosuppressed are sufficiently prioritised | 19 | 29.69 | 24 | 37.50 | 3 | 4.69 | 9 | 14.06 | 9 | 14.06 | 67.19 | 28.13 | Contested |
| COVID-19 no longer poses a threat to the immunocompromised | 41 | 64.06 | 16 | 25.00 | 2 | 3.13 | 4 | 6.25 | 1 | 1.56 | 89.06 | 7.81 | Disagree |
| Vulnerability to COVID-19 is similar for all immunosuppressed subgroups | 49 | 76.56 | 11 | 17.19 | 2 | 3.13 | 1 | 1.56 | 1 | 1.56 | 93.75 | 3.13 | Disagree |
| It is easy to pick out the immunosuppressed subgroups at most risk for COVID-19 | 14 | 21.88 | 32 | 50.00 | 6 | 9.38 | 9 | 14.06 | 3 | 4.69 | 71.88 | 18.75 | Contested |
| COVID most at-risk subgroups are the same as seen in influenza | 6 | 9.38 | 22 | 34.38 | 13 | 20.31 | 16 | 25.00 | 7 | 10.94 | 43.75 | 35.94 | Contested |
| COVID care is overly one size fits all | 10 | 15.63 | 9 | 14.06 | 5 | 7.81 | 23 | 35.94 | 17 | 26.56 | 29.69 | 62.50 | Contested |

*Clinical consensus statements - dependencies*

| **Statement** | **Strongly disagree** | **%** | **Slightly disagree** | **%** | **Unsure** | **%** | **Slightly agree** | **%** | **Strongly agree** | **%** | **SUM disagree** | **SUM agree** | **Determination** |
| --- | --- | --- | --- | --- | --- | --- | --- | --- | --- | --- | --- | --- | --- |
| The patient's cancer is in remission | 2 | 3.13 | 16 | 25.00 | 11 | 17.19 | 16 | 25.00 | 19 | 29.69 | 28.13 | 54.69 | Contested |
| The patient's cancer is current untreated | 11 | 17.19 | 27 | 42.19 | 11 | 17.19 | 11 | 17.19 | 4 | 6.25 | 59.38 | 23.44 | Contested |
| The patient's cancer is classed as early stage (I) | 4 | 6.25 | 15 | 23.44 | 11 | 17.19 | 23 | 35.94 | 11 | 17.19 | 29.69 | 53.13 | Contested |
| The patient's HIV is drug-managed | 1 | 1.56 | 6 | 9.38 | 12 | 18.75 | 23 | 35.94 | 22 | 34.38 | 10.94 | 70.31 | Contested |
| The patient's inflammatory disease is currently untreated | 8 | 12.50 | 21 | 32.81 | 16 | 25.00 | 12 | 18.75 | 7 | 10.94 | 45.31 | 29.69 | Contested |
| The patient has been on immunosuppressive treatment for less than 6 months | 26 | 40.63 | 25 | 39.06 | 9 | 14.06 | 4 | 6.25 | 0 | 0.00 | 79.69 | 6.25 | Disagree |
| The patient's immunosuppressive regimen discontinued more than 6 months ago | 5 | 7.81 | 20 | 31.25 | 11 | 17.19 | 18 | 28.13 | 10 | 15.63 | 39.06 | 43.75 | Contested |
| The patient's immunosuppressive regimen discontinued more than 12 months ago | 2 | 3.13 | 14 | 21.88 | 10 | 15.63 | 15 | 23.44 | 23 | 35.94 | 25.00 | 59.38 | Contested |
| The patient is on low-dose immunosuppressives | 10 | 15.63 | 22 | 34.38 | 10 | 15.63 | 13 | 20.31 | 9 | 14.06 | 50.00 | 34.38 | Contested |

*Higher vs lower COVID-19 risk categorised data*

| **Diagnosis** | **Higher Risk (n)** | **%** | **Lower Risk (n)** | **%** | **Consensus categorisation** |
| --- | --- | --- | --- | --- | --- |
| Chronic stress | 7 | 10.94 | 57 | 89.06 | Lower |
| Sleep deprivation | 7 | 10.94 | 57 | 89.06 | Lower |
| Cochlear implant | 11 | 17.19 | 53 | 82.81 | Lower |
| Stunting | 11 | 17.19 | 53 | 82.81 | Lower |
| Drug-managed HIV | 14 | 21.88 | 50 | 78.13 | Lower |
| Ophthalmologic autoimmune | 15 | 23.44 | 49 | 76.56 | Lower |
| Cerebrospinal fluid leak | 17 | 26.56 | 47 | 73.44 | Lower |
| Anorexia nervosa | 21 | 32.81 | 43 | 67.19 | Lower |
| Autoimmune skin diseases | 23 | 35.94 | 41 | 64.06 | Lower |
| Endocrine autoimmune | 23 | 35.94 | 41 | 64.06 | Lower |
| Burn injuries | 24 | 37.50 | 40 | 62.50 | Lower |
| Gastrointestinal autoimmune | 30 | 46.88 | 34 | 53.13 | Lower |
| T2D | 31 | 48.44 | 33 | 51.56 | Lower |
| Malnutrition | 32 | 50.00 | 32 | 50.00 | Lower |
| Haematologic autoimmune | 33 | 51.56 | 31 | 48.44 | Higher |
| HIV infection | 33 | 51.56 | 31 | 48.44 | Higher |
| Neurologic autoimmune | 34 | 53.13 | 30 | 46.88 | Higher |
| Sickle cell disease | 34 | 53.13 | 30 | 46.88 | Higher |
| Nephrotic syndrome | 35 | 54.69 | 29 | 45.31 | Higher |
| Pregnancy | 36 | 56.25 | 28 | 43.75 | Higher |
| Asplenia | 38 | 59.38 | 26 | 40.63 | Higher |
| Solid tumours | 38 | 59.38 | 26 | 40.63 | Higher |
| Rheumatological disorders | 40 | 62.50 | 24 | 37.50 | Higher |
| Aplastic anemia | 41 | 64.06 | 23 | 35.94 | Higher |
| Renal autoimmune | 42 | 65.63 | 22 | 34.38 | Higher |
| Down syndrome | 44 | 68.75 | 20 | 31.25 | Higher |
| Chronic kidney disease | 45 | 70.31 | 19 | 29.69 | Higher |
| Islet transplant | 45 | 70.31 | 19 | 29.69 | Higher |
| Dialysis | 52 | 81.25 | 12 | 18.75 | Higher |
| Gernalised malignancies (metastasis) | 53 | 82.81 | 11 | 17.19 | Higher |
| Untreated HIV | 55 | 85.94 | 9 | 14.06 | Higher |
| Actively-treated malignancies | 56 | 87.50 | 8 | 12.50 | Higher |
| Underlying aberrant immunity | 60 | 93.75 | 4 | 6.25 | Higher |
| AIDS | 62 | 96.88 | 2 | 3.13 | Higher |
| Genetic disorders of immune system (PIDs) | 62 | 96.88 | 2 | 3.13 | Higher |
| Bone marrow | 63 | 98.44 | 1 | 1.56 | Higher |
| Haematological malignancies | 64 | 100.00 | 0 | 0.00 | Higher |
| Multi-organ transplant | 64 | 100.00 | 0 | 0.00 | Higher |
| SOT | 64 | 100.00 | 0 | 0.00 | Higher |
| Stem cell transplantation | 64 | 100.00 | 0 | 0.00 | Higher |

*Excess vulnerability data*

| **Diagnosis** | **No additional vulnerability** | **%** | **Slightly elevated vulnerability** | **%** | **Significantly elevated vulnerability** | **%** | **High vulnerability** | **%** | **Extremely high vulnerability** | **%** | **SUM minimal additional vulnerability** | **SUM major additional vulnerability** | **Determination** |
| --- | --- | --- | --- | --- | --- | --- | --- | --- | --- | --- | --- | --- | --- |
| Solid organ transplantation | 1 | 1.56 | 0 | 0.00 | 8 | 12.50 | 22 | 34.38 | 33 | 51.56 | 1.56 | 85.94 | Major |
| Bone marrow transplantation | 1 | 1.56 | 1 | 1.56 | 5 | 7.81 | 14 | 21.88 | 43 | 67.19 | 3.13 | 89.06 | Major |
| Stem cell transplantation | 1 | 1.56 | 1 | 1.56 | 6 | 9.38 | 13 | 20.31 | 43 | 67.19 | 3.13 | 87.50 | Major |
| Multi organ transplantation | 1 | 1.56 | 0 | 0.00 | 7 | 10.94 | 15 | 23.44 | 41 | 64.06 | 1.56 | 87.50 | Major |
| Islet transplantation | 4 | 6.25 | 15 | 23.44 | 18 | 28.13 | 15 | 23.44 | 12 | 18.75 | 29.69 | 42.19 | Contested |
| Haematological malignancies | 2 | 3.13 | 1 | 1.56 | 5 | 7.81 | 23 | 35.94 | 33 | 51.56 | 4.69 | 87.50 | Major |
| Solid tumours | 2 | 3.13 | 13 | 20.31 | 25 | 39.06 | 20 | 31.25 | 4 | 6.25 | 23.44 | 37.50 | Contested |
| Generalised malignancies | 2 | 3.13 | 6 | 9.38 | 23 | 35.94 | 21 | 32.81 | 12 | 18.75 | 12.50 | 51.56 | Contested |
| Actively treated malignancy | 1 | 1.56 | 5 | 7.81 | 20 | 31.25 | 22 | 34.38 | 16 | 25.00 | 9.38 | 59.38 | Contested |
| HIV infection | 5 | 7.81 | 29 | 45.31 | 19 | 29.69 | 6 | 9.38 | 5 | 7.81 | 53.13 | 17.19 | Contested |
| Drug-managed HIV | 25 | 39.06 | 24 | 37.50 | 12 | 18.75 | 1 | 1.56 | 2 | 3.13 | 76.56 | 4.69 | Minimal |
| Untreated HIV | 4 | 6.25 | 10 | 15.63 | 20 | 31.25 | 16 | 25.00 | 14 | 21.88 | 21.88 | 46.88 | Contested |
| AIDS | 1 | 1.56 | 3 | 4.69 | 16 | 25.00 | 18 | 28.13 | 26 | 40.63 | 6.25 | 68.75 | Contested |
| Genetic disorders affecting the immune system | 1 | 1.56 | 3 | 4.69 | 9 | 14.06 | 25 | 39.06 | 26 | 40.63 | 6.25 | 79.69 | Major |
| Down syndrome | 6 | 9.38 | 18 | 28.13 | 20 | 31.25 | 14 | 21.88 | 6 | 9.38 | 37.50 | 31.25 | Contested |
| Autoimmune skin diseases | 13 | 20.31 | 27 | 42.19 | 18 | 28.13 | 4 | 6.25 | 2 | 3.13 | 62.50 | 9.38 | Contested |
| Rheumatological disorders | 6 | 9.38 | 16 | 25.00 | 27 | 42.19 | 13 | 20.31 | 2 | 3.13 | 34.38 | 23.44 | Contested |
| Gastrointestinal autoimmune | 7 | 10.94 | 28 | 43.75 | 19 | 29.69 | 8 | 12.50 | 2 | 3.13 | 54.69 | 15.63 | Contested |
| Endocrine autoimmune | 14 | 21.88 | 29 | 45.31 | 15 | 23.44 | 5 | 7.81 | 1 | 1.56 | 67.19 | 9.38 | Contested |
| Neurologic autoimmune | 6 | 9.38 | 23 | 35.94 | 23 | 35.94 | 9 | 14.06 | 3 | 4.69 | 45.31 | 18.75 | Contested |
| Haematologic autoimmune | 11 | 17.19 | 22 | 34.38 | 21 | 32.81 | 7 | 10.94 | 3 | 4.69 | 51.56 | 15.63 | Contested |
| Renal autoimmune | 5 | 7.81 | 17 | 26.56 | 22 | 34.38 | 16 | 25.00 | 4 | 6.25 | 34.38 | 31.25 | Contested |
| Opthalmologic autoimmune | 24 | 37.50 | 24 | 37.50 | 9 | 14.06 | 6 | 9.38 | 1 | 1.56 | 75.00 | 10.94 | Minimal |
| Underlying aberrant immunity | 1 | 1.56 | 9 | 14.06 | 11 | 17.19 | 21 | 32.81 | 22 | 34.38 | 15.63 | 67.19 | Contested |
| Type 2 Diabetes | 12 | 18.75 | 24 | 37.50 | 18 | 28.13 | 8 | 12.50 | 2 | 3.13 | 56.25 | 15.63 | Contested |
| Asplenia | 14 | 21.88 | 19 | 29.69 | 16 | 25.00 | 9 | 14.06 | 6 | 9.38 | 51.56 | 23.44 | Contested |
| Aplastic anaemia | 8 | 12.50 | 21 | 32.81 | 13 | 20.31 | 12 | 18.75 | 10 | 15.63 | 45.31 | 34.38 | Contested |
| Sickle cell | 15 | 23.44 | 19 | 29.69 | 16 | 25.00 | 9 | 14.06 | 5 | 7.81 | 53.13 | 21.88 | Contested |
| Chronic kidney disease | 6 | 9.38 | 16 | 25.00 | 25 | 39.06 | 13 | 20.31 | 4 | 6.25 | 34.38 | 26.56 | Contested |
| Nephrotic disease | 11 | 17.19 | 14 | 21.88 | 25 | 39.06 | 7 | 10.94 | 7 | 10.94 | 39.06 | 21.88 | Contested |
| Dialysis | 5 | 7.81 | 10 | 15.63 | 22 | 34.38 | 13 | 20.31 | 14 | 21.88 | 23.44 | 42.19 | Contested |
| Cerebrospinal fluid leak | 29 | 45.31 | 16 | 25.00 | 12 | 18.75 | 5 | 7.81 | 2 | 3.13 | 70.31 | 10.94 | Contested |
| Cochlear implant | 37 | 57.81 | 15 | 23.44 | 7 | 10.94 | 4 | 6.25 | 1 | 1.56 | 81.25 | 7.81 | Minimal |
| Burn injuries | 22 | 34.38 | 22 | 34.38 | 11 | 17.19 | 7 | 10.94 | 2 | 3.13 | 68.75 | 14.06 | Contested |
| Pregnancy | 9 | 14.06 | 24 | 37.50 | 21 | 32.81 | 5 | 7.81 | 5 | 7.81 | 51.56 | 15.63 | Contested |
| Chronic stress | 35 | 54.69 | 24 | 37.50 | 5 | 7.81 | 0 | 0.00 | 0 | 0.00 | 92.19 | 0.00 | Minimal |
| Sleep deprivation | 38 | 59.38 | 18 | 28.13 | 8 | 12.50 | 0 | 0.00 | 0 | 0.00 | 87.50 | 0.00 | Minimal |
| Malnutrition | 15 | 23.44 | 26 | 40.63 | 15 | 23.44 | 6 | 9.38 | 2 | 3.13 | 64.06 | 12.50 | Contested |
| Stunting | 32 | 50.00 | 22 | 34.38 | 8 | 12.50 | 1 | 1.56 | 1 | 1.56 | 84.38 | 3.13 | Minimal |
| Anorexia nervosa | 25 | 39.06 | 23 | 35.94 | 12 | 18.75 | 3 | 4.69 | 1 | 1.56 | 75.00 | 6.25 | Minimal |

**Round 2 Interim Data**

*Evaluation of risk-categorised phenotype draft*

| **Statement** | **Strongly disagree** | **%** | **Somewhat disagree** | **%** | **Unsure** | **%** | **Somewhat agree** | **%** | **Strongly agree** | **%** | **SUM disagree** | **SUM agree** | **Determination** |
| --- | --- | --- | --- | --- | --- | --- | --- | --- | --- | --- | --- | --- | --- |
| Accurate representation of results from last round | 1 | 1.67 | 1 | 1.67 | 5 | 8.33 | 26 | 43.33 | 27 | 45.00 | 3.33 | 88.33 | Agree |
| Correct higher/lower risk allocation | 0 | 0.00 | 8 | 13.33 | 1 | 1.67 | 37 | 61.67 | 14 | 23.33 | 13.33 | 85.00 | Agree |
| Step forward for representing Immunosuppressed outcomes | 2 | 3.33 | 3 | 5.00 | 4 | 6.67 | 25 | 41.67 | 26 | 43.33 | 8.33 | 85.00 | Agree |
| Useful in other disease use-cases | 0 | 0.00 | 7 | 11.67 | 9 | 15.00 | 27 | 45.00 | 17 | 28.33 | 11.67 | 73.33 | Contested |

*Evaluation of risk-stratified phenotype draft*

| **Statement** | **Strongly disagree** | **%** | **Somewhat disagree** | **%** | **Unsure** | **%** | **Somewhat agree** | **%** | **Strongly agree** | **%** | **SUM disagree** | **SUM agree** | **Determination** |
| --- | --- | --- | --- | --- | --- | --- | --- | --- | --- | --- | --- | --- | --- |
| Accurate representation of results from last round | 1 | 1.67 | 2 | 3.33 | 4 | 6.67 | 24 | 40.00 | 29 | 48.33 | 5.00 | 88.33 | Agree |
| Levels in correct order of risk | 1 | 1.67 | 5 | 8.33 | 5 | 8.33 | 34 | 56.67 | 15 | 25.00 | 10.00 | 81.67 | Agree |
| Conditions are correctly allocated to levels | 2 | 3.33 | 7 | 11.67 | 7 | 11.67 | 25 | 41.67 | 19 | 31.67 | 15.00 | 73.33 | Contested |
| Step forward for representing Immunosuppressed outcomes | 2 | 3.33 | 1 | 1.67 | 10 | 16.67 | 24 | 40.00 | 23 | 38.33 | 5.00 | 78.33 | Agree |
| Useful in other disease domains | 1 | 1.67 | 7 | 11.67 | 15 | 25.00 | 24 | 40.00 | 13 | 21.67 | 13.33 | 61.67 | Contested |

*Appropriateness for inclusion in a phenotype of ‘adult immunosuppression’ data*

| **Diagnosis** | **Not appropriate** | **%** | **Somewhat inappropriate** | **%** | **Unsure** | **%** | **Somewhat appropriate** | **%** | **Appropriate** | **%** | **SUM disagree** | **SUM agree** | **Determination** |
| --- | --- | --- | --- | --- | --- | --- | --- | --- | --- | --- | --- | --- | --- |
| Multi-organ transplantation | 0 | 0.00 | 0 | 0.00 | 1 | 1.67 | 1 | 1.67 | 58 | 96.67 | 0.00 | 98.34 | Appropriate |
| Islet transplantation | 1 | 1.67 | 2 | 3.33 | 10 | 16.67 | 20 | 33.33 | 27 | 45.00 | 5.00 | 78.33 | Appropriate |
| Solid tumours | 0 | 0.00 | 3 | 5.00 | 8 | 13.33 | 25 | 41.67 | 24 | 40.00 | 5.00 | 81.67 | Appropriate |
| Generalised malignancies (metastasis) | 0 | 0.00 | 1 | 1.67 | 4 | 6.67 | 16 | 26.67 | 39 | 65.00 | 1.67 | 91.67 | Appropriate |
| Drug-managed HIV | 8 | 13.33 | 23 | 38.33 | 13 | 21.67 | 12 | 20.00 | 4 | 6.67 | 51.66 | 26.67 | Contested |
| Untreated HIV | 1 | 1.67 | 2 | 3.33 | 2 | 3.33 | 19 | 31.67 | 36 | 60.00 | 5.00 | 91.67 | Appropriate |
| AIDS-defining illness | 0 | 0.00 | 1 | 1.67 | 0 | 0.00 | 11 | 18.33 | 48 | 80.00 | 1.67 | 98.33 | Appropriate |
| Down Syndrome | 0 | 0.00 | 7 | 11.67 | 13 | 21.67 | 27 | 45.00 | 13 | 21.67 | 11.67 | 66.67 | Contested |
| Autoimmune skin diseases | 0 | 0.00 | 15 | 25.00 | 19 | 31.67 | 22 | 36.67 | 4 | 6.67 | 25.00 | 43.34 | Contested |
| Rheumatologic disorders | 0 | 0.00 | 9 | 15.00 | 5 | 8.33 | 35 | 58.33 | 11 | 18.33 | 15.00 | 76.66 | Appropriate |
| Gastrointestinal autoimmune disorders | 0 | 0.00 | 11 | 18.33 | 11 | 18.33 | 32 | 53.33 | 6 | 10.00 | 18.33 | 63.33 | Contested |
| Endocrine autoimmune disorders | 2 | 3.33 | 21 | 35.00 | 15 | 25.00 | 19 | 31.67 | 3 | 5.00 | 38.33 | 36.67 | Contested |
| Neurologic autoimmune disorders | 0 | 0.00 | 10 | 16.67 | 13 | 21.67 | 31 | 51.67 | 6 | 10.00 | 16.67 | 61.67 | Contested |
| Hematologic autoimmune disorders | 1 | 1.67 | 8 | 13.33 | 16 | 26.67 | 26 | 43.33 | 9 | 15.00 | 15.00 | 58.33 | Contested |
| Renal autoimmune disorders | 0 | 0.00 | 6 | 10.00 | 10 | 16.67 | 30 | 50.00 | 14 | 23.33 | 10.00 | 73.33 | Contested |
| Ophthalmologic autoimmune disorders | 4 | 6.67 | 23 | 38.33 | 15 | 25.00 | 14 | 23.33 | 4 | 6.67 | 45.00 | 30.00 | Contested |
| Underlying aberrant immunity | 0 | 0.00 | 1 | 1.67 | 1 | 1.67 | 17 | 28.33 | 41 | 68.33 | 1.67 | 96.66 | Appropriate |
| Type 2 Diabetes | 6 | 10.00 | 21 | 35.00 | 10 | 16.67 | 19 | 31.67 | 4 | 6.67 | 45.00 | 38.34 | Contested |
| Asplenia | 5 | 8.33 | 6 | 10.00 | 7 | 11.67 | 21 | 35.00 | 21 | 35.00 | 18.33 | 70.00 | Contested |
| Aplastic anaemia | 1 | 1.67 | 8 | 13.33 | 5 | 8.33 | 20 | 33.33 | 26 | 43.33 | 15.00 | 76.66 | Appropriate |
| Sickle cell disease | 5 | 8.33 | 10 | 16.67 | 14 | 23.33 | 20 | 33.33 | 11 | 18.33 | 25.00 | 51.66 | Contested |
| Chronic kidney disease | 1 | 1.67 | 9 | 15.00 | 11 | 18.33 | 28 | 46.67 | 11 | 18.33 | 16.67 | 65.00 | Contested |
| Nephrotic disease | 1 | 1.67 | 9 | 15.00 | 9 | 15.00 | 26 | 43.33 | 15 | 25.00 | 16.67 | 68.33 | Contested |
| Dialysis | 0 | 0.00 | 4 | 6.67 | 4 | 6.67 | 27 | 45.00 | 25 | 41.67 | 6.67 | 86.67 | Appropriate |
| Cerebrospinal fluid leak | 11 | 18.33 | 16 | 26.67 | 17 | 28.33 | 9 | 15.00 | 7 | 11.67 | 45.00 | 26.67 | Contested |
| Cochlear implant | 19 | 31.67 | 19 | 31.67 | 12 | 20.00 | 5 | 8.33 | 5 | 8.33 | 63.34 | 16.66 | Contested |
| Burn injuries | 7 | 11.67 | 17 | 28.33 | 12 | 20.00 | 19 | 31.67 | 5 | 8.33 | 40.00 | 40.00 | Contested |
| Pregnancy | 10 | 16.67 | 12 | 20.00 | 6 | 10.00 | 25 | 41.67 | 7 | 11.67 | 36.67 | 53.34 | Contested |
| Chronic stress | 35 | 58.33 | 11 | 18.33 | 9 | 15.00 | 4 | 6.67 | 1 | 1.67 | 76.66 | 8.34 | Inappropriate |
| Sleep deprivation | 36 | 60.00 | 11 | 18.33 | 10 | 16.67 | 2 | 3.33 | 1 | 1.67 | 78.33 | 5.00 | Inappropriate |
| Malnutrition | 18 | 30.00 | 10 | 16.67 | 13 | 21.67 | 16 | 26.67 | 3 | 5.00 | 46.67 | 31.67 | Contested |
| Stunting | 27 | 45.00 | 15 | 25.00 | 14 | 23.33 | 3 | 5.00 | 1 | 1.67 | 70.00 | 6.67 | Contested |
| Anorexia | 25 | 41.67 | 9 | 15.00 | 14 | 23.33 | 10 | 16.67 | 2 | 3.33 | 56.67 | 20.00 | Contested |
| SOT | 0 | 0.00 | 0 | 0.00 | 0 | 0.00 | 2 | 3.33 | 58 | 96.67 | 0.00 | 100.00 | Appropriate |
| Bone marrow transplantation | 0 | 0.00 | 0 | 0.00 | 0 | 0.00 | 2 | 3.33 | 58 | 96.67 | 0.00 | 100.00 | Appropriate |
| Stem cell transplantation | 0 | 0.00 | 0 | 0.00 | 0 | 0.00 | 1 | 1.67 | 59 | 98.33 | 0.00 | 100.00 | Appropriate |
| Haematological malignancies | 0 | 0.00 | 0 | 0.00 | 0 | 0.00 | 3 | 5.00 | 57 | 95.00 | 0.00 | 100.00 | Appropriate |
| Actively treated malignancy | 0 | 0.00 | 0 | 0.00 | 1 | 1.67 | 12 | 20.00 | 47 | 78.33 | 0.00 | 98.33 | Appropriate |
| HIV infection | 3 | 5.00 | 28 | 46.67 | 7 | 11.67 | 16 | 26.67 | 6 | 10.00 | 51.67 | 36.67 | Contested |
| Genetic disorders affecting the immune system | 0 | 0.00 | 2 | 3.33 | 2 | 3.33 | 11 | 18.33 | 45 | 75.00 | 3.33 | 93.33 | Appropriate |
| Lupus | 0 | 0.00 | 2 | 3.33 | 10 | 16.67 | 23 | 38.33 | 25 | 41.67 | 3.33 | 80.00 | Appropriate |
| Rheumatoid Arthritis | 0 | 0.00 | 4 | 6.67 | 7 | 11.67 | 35 | 58.33 | 14 | 23.33 | 6.67 | 81.67 | Appropriate |
| Inflammatory bowel disease | 0 | 0.00 | 9 | 15.00 | 12 | 20.00 | 28 | 46.67 | 11 | 18.33 | 15.00 | 65.00 | Contested |
| Scleroderma | 0 | 0.00 | 10 | 16.67 | 13 | 21.67 | 24 | 40.00 | 13 | 21.67 | 16.67 | 61.67 | Contested |
| Psoriasis | 4 | 6.67 | 19 | 16.00 | 16 | 26.67 | 18 | 30.00 | 3 | 5.00 | 22.67 | 35.00 | Contested |

*Appropriateness of panellist recommendations for inclusion in a phenotype of ‘adult immunosuppression’ data*

| **Diagnosis** | **Not appropriate (n)** | **%** | **Somewhat inappropriate (n)** | **%** | **Unsure (n)** | **%** | **Somewhat appropriate (n)** | **%** | **Appropriate (n)** | **%** | **SUM disagree** | **SUM agree** | **Determination** |
| --- | --- | --- | --- | --- | --- | --- | --- | --- | --- | --- | --- | --- | --- |
| Cystic fibrosis | 3 | 5.00 | 10 | 16.67 | 5 | 8.33 | 21 | 35.00 | 21 | 35.00 | 21.67 | 70.00 | Contested |
| COPD | 4 | 6.67 | 14 | 23.33 | 8 | 13.33 | 26 | 43.33 | 8 | 13.33 | 30.00 | 56.67 | Contested |
| Liver disease | 0 | 0.00 | 10 | 16.67 | 7 | 11.67 | 30 | 50.00 | 13 | 21.67 | 16.67 | 71.67 | Contested |
| ME/CFS | 27 | 45.00 | 13 | 21.67 | 17 | 28.33 | 2 | 3.33 | 1 | 1.67 | 66.67 | 5.00 | Contested |
| Skin grafts | 10 | 16.67 | 14 | 23.33 | 16 | 26.67 | 16 | 26.67 | 4 | 6.67 | 40.00 | 33.33 | Contested |
| Obesity | 11 | 18.33 | 20 | 33.33 | 10 | 16.67 | 16 | 26.67 | 3 | 5.00 | 51.67 | 31.67 | Contested |
| Preterm births | 2 | 3.33 | 12 | 20.00 | 13 | 21.67 | 19 | 31.67 | 14 | 23.33 | 23.33 | 55.00 | Contested |

*Clinical consensus statements - general*

| **Statement** | **Strongly disagree** | **%** | **Slightly disagree** | **%** | **Unsure** | **%** | **Slightly agree** | **%** | **Strongly agree** | **%** | **SUM disagree** | **SUM agree** | **Determination** |
| --- | --- | --- | --- | --- | --- | --- | --- | --- | --- | --- | --- | --- | --- |
| Immunosuppression is poorly defined | 0 | 0.00 | 4 | 6.25 | 1 | 1.67 | 25 | 41.67 | 30 | 50.00 | 6.25 | 91.67 | Agree |
| Current clinical definition is too expansive | 1 | 1.67 | 16 | 25.00 | 7 | 11.67 | 28 | 46.67 | 8 | 13.33 | 26.67 | 60.00 | Contested |
| Current clinical definition isn't expansive enough | 6 | 10.00 | 25 | 39.06 | 11 | 18.33 | 17 | 28.33 | 1 | 1.67 | 49.06 | 30.00 | Contested |
| Immunosuppressed and immunocompromised are exchangeable terms | 17 | 28.33 | 21 | 32.81 | 5 | 8.33 | 11 | 18.33 | 6 | 10.00 | 61.15 | 28.33 | Contested |
| Immunosuppressed are sufficiently prioritised | 17 | 28.33 | 20 | 31.25 | 5 | 8.33 | 15 | 25.00 | 3 | 5.00 | 59.58 | 30.00 | Contested |
| COVID-19 no longer poses a threat to the immunocompromised | 42 | 70.00 | 16 | 25.00 | 0 | 0.00 | 2 | 3.33 | 0 | 0.00 | 95.00 | 3.33 | Disagree |
| Vulnerability to COVID-19 is similar for all immunosuppressed subgroups | 44 | 73.33 | 14 | 21.88 | 0 | 0.00 | 0 | 0.00 | 2 | 3.33 | 95.21 | 3.33 | Disagree |
| It is easy to pick out the immunosuppressed subgroups at most risk for COVID-19 | 9 | 15.00 | 35 | 54.69 | 3 | 5.00 | 13 | 21.67 | 0 | 0.00 | 69.69 | 21.67 | Contested |
| COVID most at-risk subgroups are the same as seen in influenza | 6 | 10.00 | 23 | 35.94 | 8 | 13.33 | 20 | 33.33 | 3 | 5.00 | 45.94 | 38.33 | Contested |
| COVID care is overly one size fits all | 4 | 6.67 | 10 | 15.63 | 3 | 5.00 | 33 | 55.00 | 10 | 16.67 | 22.29 | 71.67 | Contested |

*Clinical consensus statements - dependencies*

| **Statement** | **Strongly disagree** | **%** | **Slightly disagree** | **%** | **Unsure** | **%** | **Slightly agree** | **%** | **Strongly agree** | **%** | **SUM disagree** | **SUM agree** | **Determination** |
| --- | --- | --- | --- | --- | --- | --- | --- | --- | --- | --- | --- | --- | --- |
| The patient's cancer is in remission | 1 | 1.67 | 11 | 18.33 | 6 | 10.00 | 26 | 43.33 | 16 | 26.67 | 20.00 | 70.00 | Contested |
| The patient's cancer is current untreated | 13 | 21.67 | 19 | 31.67 | 5 | 8.33 | 9 | 15.00 | 4 | 6.67 | 53.33 | 21.67 | Contested |
| The patient's cancer is classed as early stage (I) | 1 | 1.67 | 15 | 25.00 | 10 | 16.67 | 24 | 40.00 | 10 | 16.67 | 26.67 | 56.67 | Contested |
| The patient's HIV is drug-managed | 3 | 5.00 | 4 | 6.67 | 4 | 6.67 | 28 | 46.67 | 21 | 35.00 | 11.67 | 81.67 | Agree |
| The patient's inflammatory disease is currently untreated | 9 | 15.00 | 17 | 28.33 | 11 | 18.33 | 16 | 26.67 | 7 | 11.67 | 43.33 | 38.33 | Contested |
| The patient has been on immunosuppressive treatment for less than 6 months | 24 | 40.00 | 27 | 45.00 | 5 | 8.33 | 3 | 5.00 | 1 | 1.67 | 85.00 | 6.67 | Disagree |
| The patient's immunosuppressive regimen discontinued more than 6 months ago | 1 | 1.67 | 16 | 26.67 | 11 | 18.33 | 29 | 48.33 | 3 | 5.00 | 28.33 | 53.33 | Contested |
| The patient's immunosuppressive regimen discontinued more than 12 months ago | 1 | 1.67 | 6 | 10.00 | 4 | 6.67 | 29 | 48.33 | 20 | 33.33 | 11.67 | 81.67 | Agree |
| The patient's immunosuppressive regimen discontinued more than 3 years ago | 2 | 3.33 | 0 | 0.00 | 4 | 6.67 | 19 | 31.67 | 35 | 58.33 | 3.33 | 90.00 | Agree |
| The patient is on low-dose immunosuppressives | 14 | 23.33 | 26 | 43.33 | 6 | 10.00 | 11 | 18.33 | 3 | 5.00 | 66.67 | 23.33 | Contested |
| The patient is on very low-dose immunosuppressives | 7 | 11.67 | 15 | 25.00 | 9 | 15.00 | 22 | 36.67 | 7 | 11.67 | 36.67 | 48.33 | Contested |

*Higher vs lower COVID-19 risk categorised data*

| **Diagnosis** | **Higher Risk (n)** | **%** | **Lower Risk (n)** | **%** | **Consensus categorisation** |
| --- | --- | --- | --- | --- | --- |
| ME/CFS | 4 | 6.67 | 56 | 93.33 | Lower |
| Chronic stress | 6 | 10.00 | 54 | 90.00 | Lower |
| Sleep deprivation | 6 | 10.00 | 54 | 90.00 | Lower |
| Drug-managed HIV | 7 | 11.67 | 53 | 88.33 | Lower |
| Stunting | 8 | 13.33 | 52 | 86.67 | Lower |
| Cochlear implants | 11 | 18.33 | 49 | 81.67 | Lower |
| Anorexia | 12 | 20.00 | 48 | 80.00 | Lower |
| Cerebrospinal fluid leak | 14 | 23.33 | 46 | 76.67 | Lower |
| Opthalmologic autoimmune | 16 | 26.67 | 44 | 73.33 | Contested |
| Burn injuries | 17 | 28.33 | 43 | 71.67 | Contested |
| Malnutrition | 17 | 28.33 | 43 | 71.67 | Contested |
| Skin grafts | 17 | 28.33 | 43 | 71.67 | Contested |
| T2D | 20 | 33.33 | 40 | 66.67 | Contested |
| Endocrine autoimmune | 22 | 36.67 | 38 | 63.33 | Contested |
| Autoimmune skin diseases | 23 | 38.33 | 37 | 61.67 | Contested |
| Gastrointestinal autoimmune | 28 | 46.67 | 32 | 53.33 | Contested |
| Obesity | 29 | 48.33 | 31 | 51.67 | Contested |
| Pregnancy | 33 | 55.00 | 27 | 45.00 | Contested |
| Preterm births | 33 | 55.00 | 27 | 45.00 | Contested |
| Asplenia | 36 | 60.00 | 24 | 40.00 | Contested |
| Neurologic autoimmune | 36 | 60.00 | 24 | 40.00 | Contested |
| HIV infection (all levels) | 37 | 61.67 | 23 | 38.33 | Contested |
| Down syndrome | 39 | 65.00 | 21 | 35.00 | Contested |
| Liver disease | 39 | 65.00 | 21 | 35.00 | Contested |
| Sickle cell disease | 39 | 65.00 | 21 | 35.00 | Contested |
| Haematologic autoimmune | 41 | 68.33 | 19 | 31.67 | Contested |
| COPD | 42 | 70.00 | 18 | 30.00 | Contested |
| Cystic fibrosis | 42 | 70.00 | 18 | 30.00 | Contested |
| Nephrotic syndrome | 43 | 71.67 | 17 | 28.33 | Contested |
| Chronic kidney disease | 44 | 73.33 | 16 | 26.67 | Contested |
| Rheumatological disorders | 44 | 73.33 | 16 | 26.67 | Contested |
| Aplastic anaemia | 45 | 75.00 | 15 | 25.00 | Higher |
| Solid tumours | 45 | 75.00 | 15 | 25.00 | Higher |
| Renal autoimmune | 46 | 76.67 | 14 | 23.33 | Higher |
| Dialysis | 50 | 83.33 | 10 | 16.67 | Higher |
| Islet transplant | 52 | 86.67 | 8 | 13.33 | Higher |
| Underlying aberrant immunity | 53 | 88.33 | 7 | 11.67 | Higher |
| Generalised malignancies (metastasis) | 54 | 90.00 | 6 | 10.00 | Higher |
| Untreated HIV | 55 | 91.67 | 5 | 8.33 | Higher |
| Actively treated malignancies | 56 | 93.33 | 4 | 6.67 | Higher |
| AIDS-defining illness | 56 | 93.33 | 4 | 6.67 | Higher |
| Genetic diseases (PIDs) | 56 | 93.33 | 4 | 6.67 | Higher |
| Bone marrow | 59 | 98.33 | 1 | 1.67 | Higher |
| Haematological malignancies | 59 | 98.33 | 1 | 1.67 | Higher |
| Multi-organ transplant | 59 | 98.33 | 1 | 1.67 | Higher |
| SOT | 59 | 98.33 | 1 | 1.67 | Higher |
| Stem cell transplant | 59 | 98.33 | 1 | 1.67 | Higher |

*Excess vulnerability data*

| **Condition** | **No additional vulnerability** | **%** | **Slightly elevated vulnerability** | **%** | **Significantly elevated vulnerability** | **%** | **High vulnerability** | **%** | **Extremely high vulnerability** | **%** | **SUM minimal additional vulnerability** | **SUM major additional vulnerability** | **Determination** |
| --- | --- | --- | --- | --- | --- | --- | --- | --- | --- | --- | --- | --- | --- |
| Solid organ transplantation | 0 | 0.00 | 0 | 0.00 | 6 | 10.00 | 8 | 13.33 | 46 | 76.67 | 0.00 | 90.00 | Major |
| Bone marrow transplantation | 0 | 0.00 | 0 | 0.00 | 4 | 6.67 | 5 | 8.33 | 51 | 85.00 | 0.00 | 93.33 | Major |
| Stem cell transplanatation | 0 | 0.00 | 0 | 0.00 | 3 | 5.00 | 6 | 10.00 | 51 | 85.00 | 0.00 | 95.00 | Major |
| Multi organ transplantation | 0 | 0.00 | 0 | 0.00 | 3 | 5.00 | 6 | 10.00 | 51 | 85.00 | 0.00 | 95.00 | Major |
| Islet transplantation | 3 | 5.00 | 5 | 8.33 | 17 | 28.33 | 20 | 33.33 | 15 | 25.00 | 13.33 | 58.33 | Contested |
| Haematological malignancies | 0 | 0.00 | 1 | 1.67 | 4 | 6.67 | 16 | 26.67 | 39 | 65.00 | 1.67 | 91.67 | Major |
| Solid tumours | 0 | 0.00 | 9 | 15.00 | 19 | 31.67 | 22 | 36.67 | 10 | 16.67 | 15.00 | 53.33 | Contested |
| Generalised malignancies (metastasis) | 0 | 0.00 | 5 | 8.33 | 13 | 21.67 | 24 | 40.00 | 18 | 30.00 | 8.33 | 70.00 | Contested |
| Actively treated malignancy | 1 | 1.67 | 8 | 13.33 | 10 | 16.67 | 24 | 40.00 | 17 | 28.33 | 15.00 | 68.33 | Contested |
| HIV infection all stages | 3 | 5.00 | 29 | 48.33 | 13 | 21.67 | 12 | 20.00 | 3 | 5.00 | 53.33 | 25.00 | Contested |
| Drug-managed HIV | 23 | 38.33 | 26 | 43.33 | 9 | 15.00 | 2 | 3.33 | 0 | 0.00 | 81.67 | 3.33 | Minimal |
| Untreated HIV | 1 | 1.67 | 8 | 13.33 | 16 | 26.67 | 25 | 41.67 | 10 | 16.67 | 15.00 | 58.33 | Contested |
| AIDS-defining illness | 0 | 0.00 | 2 | 3.33 | 13 | 21.67 | 16 | 26.67 | 29 | 48.33 | 3.33 | 75.00 | Major |
| Genetic disorders affecting the immune system | 0 | 0.00 | 2 | 3.33 | 5 | 8.33 | 18 | 30.00 | 35 | 58.33 | 3.33 | 88.33 | Major |
| Down syndrome | 3 | 5.00 | 13 | 21.67 | 25 | 41.67 | 13 | 21.67 | 6 | 10.00 | 26.67 | 31.67 | Contested |
| Autoimmune skin diseases | 5 | 8.33 | 36 | 60.00 | 13 | 21.67 | 5 | 8.33 | 1 | 1.67 | 68.33 | 10.00 | Contested |
| Rheumatological disorders | 2 | 3.33 | 16 | 26.67 | 26 | 43.33 | 14 | 23.33 | 2 | 3.33 | 30.00 | 26.67 | Contested |
| Gastrointestinal autoimmune disorders | 4 | 6.67 | 31 | 51.67 | 16 | 26.67 | 9 | 15.00 | 0 | 0.00 | 58.33 | 15.00 | Contested |
| Endocrine autoimmune disorders | 10 | 16.67 | 30 | 50.00 | 14 | 23.33 | 6 | 10.00 | 0 | 0.00 | 66.67 | 10.00 | Contested |
| Neurologic autoimmune disorders | 4 | 6.67 | 27 | 45.00 | 20 | 33.33 | 9 | 15.00 | 0 | 0.00 | 51.67 | 15.00 | Contested |
| Haematologic autoimmune disorders | 5 | 8.33 | 19 | 31.67 | 22 | 36.67 | 11 | 18.33 | 3 | 5.00 | 40.00 | 23.33 | Contested |
| Renal autoimmune disorders | 3 | 5.00 | 14 | 23.33 | 23 | 38.33 | 17 | 28.33 | 3 | 5.00 | 28.33 | 33.33 | Contested |
| Opthalmologic autoimmune disorders | 17 | 28.33 | 31 | 51.67 | 8 | 13.33 | 4 | 6.67 | 0 | 0.00 | 80.00 | 6.67 | Minimal |
| Underlying aberrant immunity | 0 | 0.00 | 7 | 11.67 | 6 | 10.00 | 22 | 36.67 | 25 | 41.67 | 11.67 | 78.33 | Major |
| Type 2 diabetes mellitus | 9 | 15.00 | 29 | 48.33 | 12 | 20.00 | 10 | 16.67 | 0 | 0.00 | 63.33 | 16.67 | Contested |
| Asplenia (anatomic/ functional) | 12 | 20.00 | 18 | 30.00 | 14 | 23.33 | 13 | 21.67 | 3 | 5.00 | 50.00 | 26.67 | Contested |
| Aplastic anemia | 6 | 10.00 | 16 | 26.67 | 12 | 20.00 | 22 | 36.67 | 4 | 6.67 | 36.67 | 43.33 | Contested |
| Sickle cell disease | 8 | 13.33 | 21 | 35.00 | 17 | 28.33 | 13 | 21.67 | 1 | 1.67 | 48.33 | 23.33 | Contested |
| Chronic kidney disease | 1 | 1.67 | 17 | 28.33 | 24 | 40.00 | 18 | 30.00 | 0 | 0.00 | 30.00 | 30.00 | Contested |
| Nephrotic disease | 2 | 3.33 | 21 | 35.00 | 20 | 33.33 | 16 | 26.67 | 1 | 1.67 | 38.33 | 28.33 | Contested |
| Dialysis | 0 | 0.00 | 13 | 21.67 | 17 | 28.33 | 21 | 35.00 | 9 | 15.00 | 21.67 | 50.00 | Contested |
| Cystic fibrosis | 5 | 8.33 | 14 | 23.33 | 21 | 35.00 | 12 | 20.00 | 8 | 13.33 | 31.67 | 33.33 | Contested |
| Chronic obstructive pulmonary disease | 6 | 10.00 | 13 | 21.67 | 21 | 35.00 | 15 | 25.00 | 5 | 8.33 | 31.67 | 33.33 | Contested |
| Liver disease (including cirrhotic) | 5 | 8.33 | 20 | 33.33 | 22 | 36.67 | 11 | 18.33 | 2 | 3.33 | 41.67 | 21.67 | Contested |
| Cerebrospinal fluid leak | 33 | 55.00 | 17 | 28.33 | 4 | 6.67 | 5 | 8.33 | 1 | 1.67 | 83.33 | 10.00 | Minimal |
| Cochlear implant | 40 | 66.67 | 10 | 16.67 | 4 | 6.67 | 5 | 8.33 | 1 | 1.67 | 83.33 | 10.00 | Minimal |
| Burn injuries | 26 | 43.33 | 17 | 28.33 | 12 | 20.00 | 5 | 8.33 | 0 | 0.00 | 71.67 | 8.33 | Contested |
| Skin graft recipients | 25 | 41.67 | 20 | 33.33 | 6 | 10.00 | 8 | 13.33 | 1 | 1.67 | 75.00 | 15.00 | Minimal |
| Pregnancy | 10 | 16.67 | 23 | 38.33 | 18 | 30.00 | 8 | 13.33 | 1 | 1.67 | 55.00 | 15.00 | Contested |
| Pre-term births | 4 | 6.67 | 26 | 43.33 | 14 | 23.33 | 15 | 25.00 | 1 | 1.67 | 50.00 | 26.67 | Contested |
| Chronic stress | 38 | 63.33 | 19 | 31.67 | 2 | 3.33 | 1 | 1.67 | 0 | 0.00 | 95.00 | 1.67 | Minimal |
| Sleep deprivation | 42 | 70.00 | 16 | 26.67 | 1 | 1.67 | 1 | 1.67 | 0 | 0.00 | 96.67 | 1.67 | Minimal |
| Malnutrition | 19 | 31.67 | 28 | 46.67 | 10 | 16.67 | 3 | 5.00 | 0 | 0.00 | 78.33 | 5.00 | Minimal |
| Stunting | 37 | 61.67 | 19 | 31.67 | 3 | 5.00 | 1 | 1.67 | 0 | 0.00 | 93.33 | 1.67 | Minimal |
| Obesity | 13 | 21.67 | 26 | 43.33 | 11 | 18.33 | 8 | 13.33 | 2 | 3.33 | 65.00 | 16.67 | Contested |
| Anorexia nervosa | 27 | 45.00 | 25 | 41.67 | 7 | 11.67 | 1 | 1.67 | 0 | 0.00 | 86.67 | 1.67 | Minimal |
| Myalgic encephalomyelitis/ Chronic fatigue syndrome | 42 | 70.00 | 16 | 26.67 | 1 | 1.67 | 1 | 1.67 | 0 | 0.00 | 96.67 | 1.67 | Minimal |

**Data presented at Final Discussion**

*Evaluation of risk-categorised phenotype draft*

| **Statement** | **Strongly disagree** | **%** | **Somewhat disagree** | **%** | **Unsure** | **%** | **Somewhat agree** | **%** | **Strongly agree** | **%** | **SUM disagree** | **SUM agree** | **Determination** |
| --- | --- | --- | --- | --- | --- | --- | --- | --- | --- | --- | --- | --- | --- |
| This version was an improvement to the last draft | 0 | 0.00 | 0 | 0.00 | 2 | 5.13 | 22 | 56.41 | 15 | 38.46 | 0.00 | 94.87 | Agree |
| Prefer three part to two part | 0 | 0.00 | 1 | 2.56 | 0 | 0.00 | 10 | 25.64 | 28 | 71.79 | 2.56 | 97.44 | Agree |
| Drug managed HIV is fair active control | 0 | 0.00 | 5 | 12.82 | 10 | 25.64 | 15 | 38.46 | 9 | 23.08 | 12.82 | 61.54 | Contested |
| Useful to compare phenotype data to active control | 0 | 0.00 | 5 | 12.82 | 8 | 20.51 | 18 | 46.15 | 8 | 20.51 | 12.82 | 66.67 | Contested |
| Phenotype is accurate reflection of last round | 0 | 0.00 | 0 | 0.00 | 3 | 7.69 | 20 | 51.28 | 16 | 41.03 | 0.00 | 92.31 | Agree |
| Correct allocation | 0 | 0.00 | 5 | 12.82 | 2 | 5.13 | 16 | 41.03 | 16 | 41.03 | 12.82 | 82.05 | Agree |
| Step forward for subdividing data | 1 | 2.56 | 1 | 2.56 | 2 | 5.13 | 13 | 33.33 | 22 | 56.41 | 5.13 | 89.74 | Agree |
| Clinically useful to test transferability to other disease domains | 1 | 2.56 | 1 | 2.56 | 9 | 23.08 | 16 | 41.03 | 12 | 30.77 | 5.13 | 71.79 | Contested |

*Evaluation of risk-stratified phenotype draft*

| **Statement** | **Strongly disagree** | **%** | **Somewhat disagree** | **%** | **Unsure** | **%** | **Somewhat agree** | **%** | **Strongly agree** | **%** | **SUM disagree** | **SUM agree** | **Determination** |
| --- | --- | --- | --- | --- | --- | --- | --- | --- | --- | --- | --- | --- | --- |
| This version was an improvement to the last draft | 0 | 0.00 | 1 | 2.56 | 3 | 7.69 | 19 | 48.72 | 16 | 41.03 | 2.56 | 89.74 | Agree |
| Accurate representation of last round | 0 | 0.00 | 1 | 2.56 | 3 | 7.69 | 18 | 46.15 | 17 | 43.59 | 2.56 | 89.74 | Agree |
| Drug managed HIV is fair active control | 1 | 2.56 | 3 | 7.69 | 12 | 30.77 | 15 | 38.46 | 8 | 20.51 | 10.26 | 58.97 | Contested |
| Useful to compare phenotype data to active control | 1 | 2.56 | 4 | 10.26 | 9 | 23.08 | 16 | 41.03 | 9 | 23.08 | 12.82 | 64.10 | Contested |
| Phenotype in correct order | 1 | 2.56 | 3 | 7.69 | 4 | 10.26 | 20 | 51.28 | 11 | 28.21 | 10.26 | 79.49 | Agree |
| Conditions correctly allocated | 1 | 2.56 | 6 | 15.38 | 1 | 2.56 | 20 | 51.28 | 11 | 28.21 | 17.95 | 79.49 | Agree |
| Step forward for subdividing data | 1 | 2.56 | 4 | 10.26 | 2 | 5.13 | 14 | 35.90 | 18 | 46.15 | 12.82 | 82.05 | Agree |
| Clinically useful to test transferability to other disease domains | 1 | 2.56 | 3 | 7.69 | 6 | 15.38 | 17 | 43.59 | 12 | 30.77 | 10.26 | 74.36 | Contested |

*Clinical consensus statements – general*

| **Statement** | **Strongly disagree** | **%** | **Somewhat disagree** | **%** | **Unsure** | **%** | **Somewhat agree** | **%** | **Strongly agree** | **%** | **SUM disagree** | **SUM agree** | **Determination** |
| --- | --- | --- | --- | --- | --- | --- | --- | --- | --- | --- | --- | --- | --- |
| Current clinical definition is too expansive | 2 | 5.13 | 4 | 10.26 | 6 | 15.38 | 14 | 35.90 | 13 | 33.33 | 15.38 | 69.23 | Contested |
| Current clinical definition isn't expansive enough | 9 | 23.08 | 17 | 43.59 | 3 | 7.69 | 9 | 23.08 | 1 | 2.56 | 66.67 | 25.64 | Contested |
| Immunosuppressed and immunocompromised are exchangeable terms | 10 | 25.64 | 17 | 43.59 | 1 | 2.56 | 6 | 15.38 | 5 | 12.82 | 69.23 | 28.21 | Contested |
| Immunosuppressed are sufficiently prioritised | 7 | 17.95 | 19 | 48.72 | 3 | 7.69 | 10 | 25.64 | 0 | 0.00 | 66.67 | 25.64 | Contested |
| It is easy to pick out the immunosuppressed subgroups at most risk for COVID-19 | 2 | 5.13 | 18 | 46.15 | 4 | 10.26 | 14 | 35.90 | 1 | 2.56 | 51.28 | 38.46 | Contested |
| COVID most at-risk subgroups are the same as seen in influenza | 5 | 12.82 | 15 | 38.46 | 5 | 12.82 | 13 | 33.33 | 1 | 2.56 | 51.28 | 35.90 | Contested |
| COVID care is overly one size fits all | 2 | 5.13 | 4 | 10.26 | 1 | 2.56 | 22 | 56.41 | 10 | 25.64 | 15.38 | 82.05 | Agree |

*Clinical consensus statements - dependencies*

| **Statement** | **Strongly disagree** | **%** | **Somewhat disagree** | **%** | **Unsure** | **%** | **Somewhat agree** | **%** | **Strongly agree** | **%** | **SUM disagree** | **SUM agree** | **Determination** |
| --- | --- | --- | --- | --- | --- | --- | --- | --- | --- | --- | --- | --- | --- |
| Cancer in remission | 3 | 7.69 | 4 | 10.26 | 2 | 5.13 | 22 | 56.41 | 8 | 20.51 | 17.95 | 76.92 | Agree |
| Cancer untreated | 7 | 17.95 | 23 | 58.97 | 2 | 5.13 | 6 | 15.38 | 1 | 2.56 | 76.92 | 17.95 | Disagree |
| Early cancer | 6 | 15.38 | 5 | 12.82 | 10 | 25.64 | 16 | 41.03 | 2 | 5.13 | 28.21 | 46.15 | Contested |
| Inflammatory disease untreated | 6 | 15.38 | 15 | 38.46 | 3 | 7.69 | 11 | 28.21 | 4 | 10.26 | 53.85 | 38.46 | Contested |
| Discontinuation of immunosuppression more than 6 months ago | 0 | 0.00 | 16 | 41.03 | 5 | 12.82 | 12 | 30.77 | 6 | 15.38 | 41.03 | 46.15 | Contested |
| Low dose pred equivalent | 9 | 23.08 | 17 | 43.59 | 5 | 12.82 | 5 | 12.82 | 3 | 7.69 | 66.67 | 20.51 | Contested |
| Very low dose pred equivalent | 3 | 7.69 | 12 | 30.77 | 4 | 10.26 | 13 | 33.33 | 7 | 17.95 | 38.46 | 51.28 | Contested |

1. **Output of Dendrogram of Excess Vulnerability Compared to the Immunocompetent Scores**

1. **Written Summary of Final Discussion Groups**

**Final Discussion (Round 3): Summary Notes**

*Round 3 participants: n = 40 (pre-discussion group data provided by 39)*

1. **Phenotype 1: Risk-categorised**

*General criticisms:*

- Preference for 3-part over 2-part phenotype – still appetite to see more subdivisions as complexity of these cases and specific immunodeficiencies need to be demarcated while balancing what can be implemented clinically.
  - However, given that this exercise is intended to support binary clinical decision-making (prescribe vs don’t prescribe, boost vaccinate vs don’t boost vaccinate), a case could be made that the previous two-part model was more clinically helpful. Need to give careful thought to which of the three categories should be prioritised for enhanced medical attention – just major additional risk, or also major + modifiable?
  - Need to ensure that high risk category is specific to those who would warrant enhanced medical support; Green Book specifications span 4% of population which is far too high for prioritisation.
- There is significant heterogeneity in the ‘moderate’ group – they could easily tip into high/ low categories depending on medication. Group should be renamed around consensus achieved as cannot infer non-consensus group is middling risk – **‘Consensus lower risk immunosuppressed’, ‘No Consensus’ and ‘Consensus higher risk immunosuppressed’.**
- Significant internal heterogeneity
- Influence of medications not incorporated in phenotype, but seemingly incorporated in the proposed active control
- Messy middle – easily catapulted into higher risk when medication variables are considered
- Continued emphasis that these risk phenotypes must not be used as clinical decision-making tools until fully validated in real-world data.
- Importance to be very cautious with transferability claims – write up must anchor on the prospective, but still unproven, surveillance value of subdividing wider disease outcomes in this same way. COVID is extremely idiosyncratic.

*Specific criticisms:*

- Does the lower risk category really add much value?
- Need to reconsider inclusion of wider determinants – their retention may be academically interesting, but clinically problematic if the net is cast too wide for immunosuppressed call up (vaccines, shielding etc).

1. **Phenotype 2: Multi-level hierarchy**

*General criticisms:*

- Some confusion around how this phenotype was constructed*: researcher had to provide context on parallel data inputs (balancing COVID vulnerability scores with population size considerations and need for common clinical/ anatomical denominator as opposed to stringing together disparate conditions) – data indicates that panellists did think these strata reflected results from last round, however.*
- Felt to be too complicated with not enough data to support internal nuances – first phenotype was preferred by some.
- Same criticisms of high levels of internal heterogeneity, often more influenced by medication than diagnosis. For this hierarchy to be as clinically helpful as possible, it’s vital that the highest risk patients are pulled out from the rest of the pack. Overly generalised definitions – even ‘haematological malignancy’ – swallows up unique risk profiles. Staying at the generic level will still underserve these patients. Surveillance must therefore be iterative, even after this point of subdivision – each level should be interrogated for internal subtrends.
- Slightly more contention with the 10-part hierarchy than the cruder 3-part cut.
- Concerns that this phenotype is over scoped; recommendation to restrict inclusion to those who would be considered eligible for enhanced medical support.
- Careful language is needed to ensure this is not phenotype is not positioned as an aid for clinical decision-making until rigorous validation has occurred. Must establish that the cadence of these risk levels holds when applied in real world data.
- Despite positive feedback, panellists emphasised that it was vital to not position risk as linear/ ordinal/ an even staircase. Certain levels are almost synonymous in risk with considerable departures between first 3 levels to 4^th^. 1-3, 4-7 and 8-10. Risk is not stepwise – each change in level does not denote same scale of change in risk profile. **May be best to band risk levels – reconciling phenotypes 1 and 2.**
- Specific features of COVID make this hierarchy likely disease specific.
- Careful language is also needed about the specificities of this risk cadence – not generalisable to paediatric cases/ fungal or bacterial infections without being tested. For example, because this is an adult risk cadence, it would not be appropriate for conditions where risk is highest in young children. Language therefore needs to specify that these risk categories *‘may’ be transferable*, subject to further validation; this should be considered a framework to be applied to subdividing disease surveillance in future outbreaks/ pandemics. It would be clinically valuable to look for similarities in disease surveillance, however, and to capture major areas of departure.

*Specific criticisms:*

- Confusion over why certain terms were included – *researcher had to explain ‘equal opportunities’ approach to scraping candidate conditions from parallel vaccine decision-making manuals.*
- Some panellists felt existing immunosuppressed risk hierarchies were more comprehensive and clinically accurate (e.g. CDC) – *researcher had to explain their non-compatibility with CMR surveillance data flows.*
- Need to tightly boundary all levels when operationalised e.g. what type of anaemia? What stage of CKD? Solid tumours ever or those without remission codes?
- Operationalising CKD – Later stage (4+).
- Must change autoinflammatory language to immune-mediated inflammatory disease (IMID).
- Gastro-autoimmunity should be in systemic category
- Combine bone marrow and stem cell transplantation into ‘hematopoietic stem cell transplantation’
- Overall agreement that systemic and single site demarcation is useful, however, there was some dissent on this and the instruction to caveat the generalised impacts of single-site conditions e.g. psoriasis.
- Query whether solid tumours should even be situated as high risk as they are currently.
- Concerns about inclusion of wider determinants of immunosuppression specifically – interesting academically but potentially detrimental clinically.
- Questioning inclusion of asplenia and amenia – unsure how much risk is conferred.
- Pregnancy as exceedingly high risk in certain circumstances (unvaccinated third trimester) – not to mention this represents two lives at stake.
- Some panellists took issue with the inclusion of renal forms of immunosuppression and the absence of pulmonary, liver and cardiac candidates – *researcher had to explain that these were put to panellists but did not meet* ≥*75% inclusion.*

1. **The use of drug/well-managed HIV as an active control or internal reference category:**

- General feeling that drug-managed /well-managed HIV would be a valuable internal reference point from a hypothesis generating perspective.
  - However, some confusion around suggested active control group; *researcher had to contextualise this with previous experiences conducting vaccine effectiveness research (disparities in vaccine uptake between immunocompetent and compromised invalidates comparisons).*
  - From an international perspective HIV is still a COVID risk-factor; its use as an active control would be disputed in less resourced contexts
  - Pointed out that rheumatoid arthritis could just as readily be used for this purpose.
- Drug-managed HIV cannot be in lieu of a general population comparator group – important to see how vastly different incidence rates are. General population comparator should be age-stratified, if possible.
- Automating the operationalisation of drug-managed HIV is impossible – an extract will only tell us that an HIV patient has received a drug, we don’t know if they’re taking the drug and we don’t know their prevailing immune state (e.g. CD4 count). Also, impossible to rule out drug holidays etc.
- Drug-managed HIV also has huge internal heterogeneity – when did treatment start, what is adherence level etc.?
- ‘Well-managed’ component would have to be assumptive e.g. extracting all HIV patients, excluding AIDS and conditions consistent with AIDS.
- Ideally CD4 count would be incorporated for absolute assurance on immunocompetence.

1. **General consensus statements:**

- General consensus statements will never obtain international agreement, even if run multiple times more.
- Important to identify throughlines of why consensus couldn’t be made e.g. international scope of panel, diverse interpretations of questions (e.g. disappointment in pharmaceutical companies lack of dedicated trials vs satisfaction with ringfencing attempts determined response to sufficiently prioritised question). Immunosuppressed / pregnant patients were not incorporated into pharmaceutical R&D; post-hoc decisions had to be made which was enormously underserving and potentially fatal.
- Immunocompromise and immunosuppressed are used synonymously in common parlance but this is not clinically correct. That said, even amongst clinicians there is continued confusion over where the line of differentiation is drawn and it would be helpful if consensus could be built on this specific issue. For some, the difference is one of temporality (e.g. immunocompromised as long-term, immunosuppressed as short-term), for others, one is a state (immunocompromised), the other is an action (use of immunosuppressive protocols).
- ‘One size fits all’ care for the immunosuppressed not necessarily a bad thing in all cases: given the sensitivity of the vaccine issue, creating stratifications/ stricter roll-out may just disincentivise uptake wholesale and keep circulation – and resulting dangers – higher than would be achieved without universal vaccination.
- Transferability will have to be tested rigorously – likely that viral conditions exacerbate underlying issues more than bacterial/ fungal counterparts.
- Data on immunosuppressed risk profiles for other respiratory viral infections is scant.
- Recognition that delivery of treatments/ antivirals needs to be more nuanced or qualified than it currently is.

1. **Conditionalities consensus statements:**

- Insufficient data to weigh in on the granularities – felt like splitting hairs between 6 and 12 and 36 months. Only able to build consensus when evidence base is sufficiently differentiated.
- Statements still felt overly generalised overall.
- A categorical / homogenised approach cannot ever be put forward as clinically advisable – decisions need to consider the whole patient ‘at the coal face’ of care.
- Cannot lump all cancers together; e.g. haematological malignancies are immunosuppressing irrespective of stage.
- ‘Early stage’ – difficult to operationalise in medical records.
- Stage language is also not always appropriate in cancer (e.g. grades) so ‘hard and fast’ rules based on stage should be avoided - risk is more related to treatment protocol/ cancer type.
- Patients may be untreated due to palliative care, previous non-responsiveness or ‘watch and wait’ – these are on opposite ends of risk scale, so ‘untreated’ does not reveal much about risk.
- Pharmacokinetic and pharmacodynamic nature of drug determines how long-term immunosuppression is. A short course of rituximab could still see immunosuppression 5 years later and 5-10mg still viewed as immunosuppressive in CDC.
- Equivalence to 10mg/ 20mg prednisolone was highly criticised.
- Consensus achieved on remission/ resolution – caveating on type of cancer, likelihood of resurgence, treatment received (e.g. bone marrow transplant) and time since remission began. Remission codes are also sparsely used – better to consider time constraining cancer diagnosis.

1. **Strengths of the exercise:**

- Panellists acknowledged that this is a massive and complicated exercise and congratulated the team – getting to a fine enough resolution on differential risk is a tall order
- This work is extremely valuable for driving hypothesis/insight generation – just not clinical practice until validated
- Panellists enjoyed the exercise overall – background detail was not overwhelming, information provided beforehand was just enough to set the scene
- Short and simple interface – key for retention.
- Impressive panel in terms of geographical footprint and panel calibre.
- Good balance of condition-general and condition-specific experts.
- Enjoyed its iterative nature – seeing the exact data between rounds. Other eDelphis have not provided data, just feedback that consensus was/ wasn’t reached, which didn’t help for shifting answers.
- Forced panellists out of their comfort zone/ to think about risk in a different way.
- Blind spots of individual panellists covered by wider panel.

1. **Weaknesses of the exercise:**

- Need to emphasise that these resources are not meant to be used for clinical decision-making for COVID or other disease use cases until they have gone through rigorous validation process (checking if real world data is in lock step with risk expectations of phenotypes). Further refinement may be required before these means of subdividing surveillance data are standardised in data flows.
- Frustration around inability to incorporate medication/ demographic variables that have considerable influence – if not determining role in some circumstances – on risk.
- Overly simplistic interface – inability to incorporate modifying variables e.g. medication/ comorbidities/ layering of immunosuppressive conditions. Survey was overly strict – difficult to convey nuances of expertise. This is problematic given that COVID risk all stands to nuance.
- Diagnosis does not necessarily infer immunosuppression (e.g. specific autoimmune/ autoinflammatory conditions) – diagnosis-based subdivision may incorporate non-immunosuppressed patients if they have not been treated.
- No patient and public involvement – hierarchies should be presented to a lay advisory group for feedback (may be viewed as wholly inappropriate). ‘Does this have face validity? Does this look reasonable/ make sense to you?’. Widen pool beyond EULAR PARE for this.
- Panellists felt unable to weigh in on certain topics without referring to the wider literature.
- Issues with recruitment strategy
  - Poor representation of Global South; no representation from patients; oversampling from rheumatological conditions; no representation from private sector; crude approach to just pluck for key decision-making bodies
- Group think concerns e.g. Islet transplantation moving towards upper risk strata after being featured in this position in first phenotype.
- Feeling that COVID is yesterday’s story – unsure how helpful these risk phenotypes will really be now that the immunosuppressed are almost universally vaccinated against the disease.
- PPIE efforts are essential but must be handled with great sensitivity – must ensure that press releases do not suggest these hierarchies are confirmed, validated or generalisable to paediatric/ other disease domains as this could be very disturbing to patients and misleading for clinicians.

1. **Panellist recommendations:**
2. Recommendation to involve PPIE once phenotypes are finalised; discuss study and construction process to allow patients to weigh in with their concerns.
3. Recommendation to publish phenotypes online (open source) to enable ‘translations’ and enable other international research teams to implement them in their own disease surveillance flows.
4. Recommendation to quantify the expansiveness of the immunosuppressed population – newer estimates are as large as 7-10% of the population.
5. Recommendation to consider Long COVID as an immunosuppressed condition.
6. Recommendation to create two parallel risk axes – one condition based, and one medication based. Highest level eligible for determines allocation and prioritisation. Ideally ability to denote an interaction between the two axes.
7. Recommendation that future exercises have group interaction at start of eDelphi (diamond shape) to provide line of sight into others’ thinking and specialisms from the very start. Would have been helpful to leverage the panel to determine questions set, for example.
8. **Prospective future consensus exercises:**

- Medication risk hierarchy to create parallel assessment tool (diagnosis risk vs medication risk axes)
- Consensus exercise on exact demarcations between immunosuppressed, immunodeficient and immunocompromised – tension between layman synonymity and specialist understanding of idiosyncrasies.
- Similar exercises should be run for other disease use-cases e.g. influenza, RSV, bacterial infections etc; key for pandemic preparedness – given that literature is not sufficiently differentiated, individual expert opinion must be collated
- Consensus exercise on the immunosuppressed patients most likely to experience suboptimal seroconversion after vaccination

1. **Final ratification data**
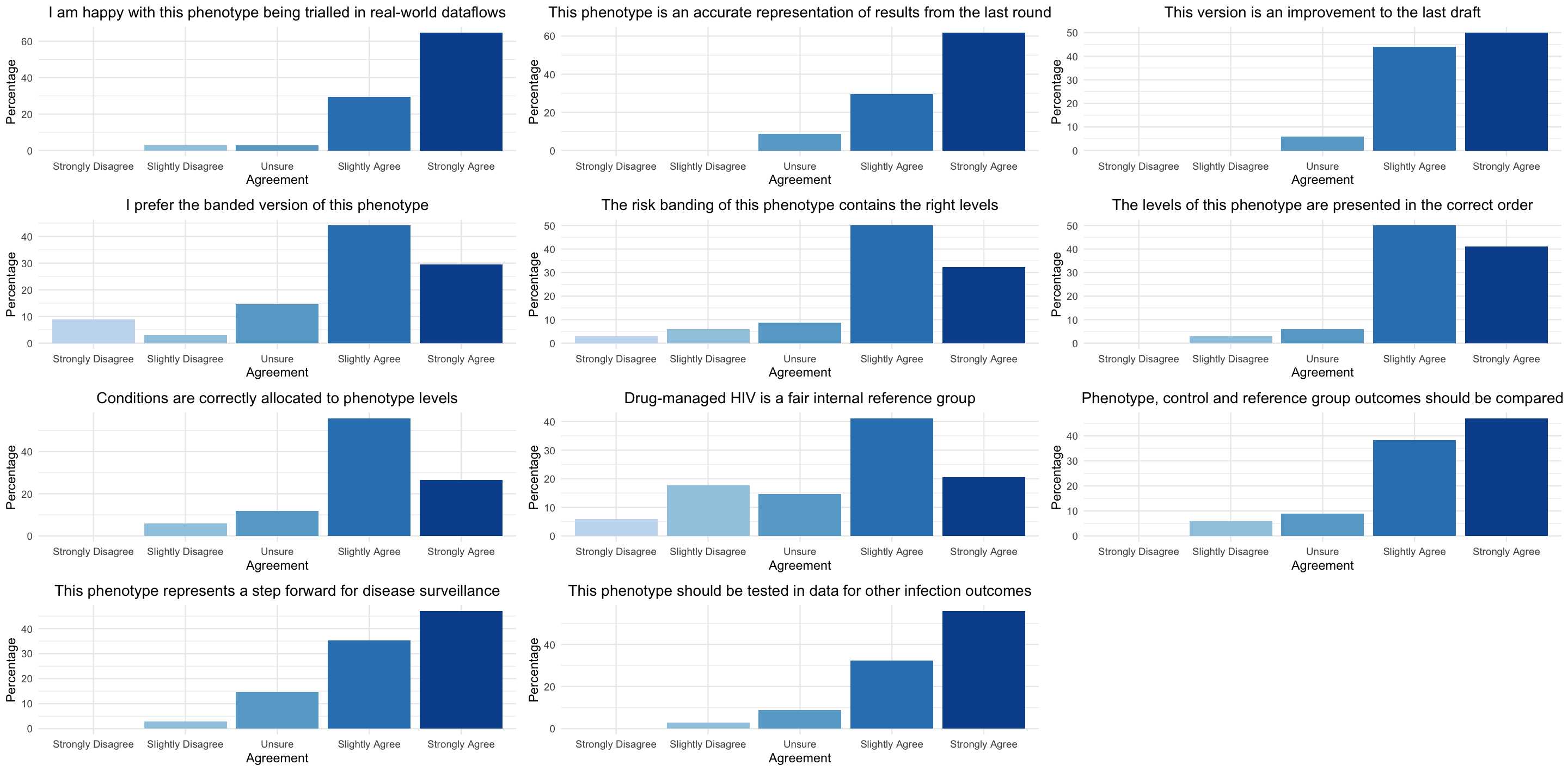


| **Statement** | **Strongly disagree** | **Strongly disagree %** | **Slightly disagree** | **Slightly disagree %** | **Unsure** | **Unsure %** | **Slightly agree** | **Slightly agree %** | **Strongly agree** | **Strongly agree %** | **Sum disagree %** | **Sum agree %** | **Determination** |
| --- | --- | --- | --- | --- | --- | --- | --- | --- | --- | --- | --- | --- | --- |
| I am happy with this phenotype being trialled within real-world data flows | 0 | 0 | 1 | 2.94 | 1 | 2.94 | 10 | 29.41 | 22 | 64.71 | 2.94 | 94.12 | Agreement |
| This phenotype is an accurate representation of results from the last round | 0 | 0 | 0 | 0 | 3 | 8.82 | 10 | 29.41 | 21 | 61.76 | 0 | 91.18 | Agreement |
| This version is an improvement to the last draft | 0 | 0 | 0 | 0 | 2 | 5.88 | 15 | 44.12 | 17 | 50 | 0 | 94.12 | Agreement |
| I prefer the banded version of this phenotype | 3 | 8.82 | 1 | 2.94 | 5 | 14.71 | 15 | 44.12 | 10 | 29.41 | 11.76 | 73.53 | Contested |
| The risk banding of this phenotype contains the right levels | 1 | 2.94 | 2 | 5.88 | 3 | 8.82 | 17 | 50 | 11 | 32.35 | 8.82 | 82.35 | Agreement |
| The levels of this phenotype are presented in the correct order (descending risk for severe COVID outcomes) | 0 | 0 | 1 | 2.94 | 2 | 5.88 | 17 | 50 | 14 | 41.18 | 2.94 | 91.18 | Agreement |
| Conditions are correctly allocated to phenotype levels | 0 | 0 | 2 | 5.88 | 4 | 11.76 | 19 | 55.88 | 9 | 26.47 | 5.88 | 82.35 | Agreement |
| Drug-managed HIV is a fair internal reference group | 2 | 5.88 | 6 | 17.65 | 5 | 14.71 | 14 | 41.18 | 7 | 20.59 | 23.53 | 61.76 | Contested |
| It would be interesting to compare phenotype, internal reference group and healthy control outcomes | 0 | 0 | 2 | 5.88 | 3 | 8.82 | 13 | 38.24 | 16 | 47.06 | 5.88 | 85.29 | Agreement |
| This phenotype represents a step forward for disease surveillance | 0 | 0 | 1 | 2.94 | 5 | 14.71 | 12 | 35.29 | 16 | 47.06 | 2.94 | 82.35 | Agreement |
| As a hypothesis generating exercise, it would be interesting to apply this phenotype to subdivide immunosuppressed outcomes for other diseases (seasonal influenza, pneumonia, RSV etc.) | 0 | 0 | 1 | 2.94 | 3 | 8.82 | 11 | 32.35 | 19 | 55.88 | 2.94 | 88.24 | Agreement |

1. **All phenotype drafts**

**DRAFT AFTER ROUND 1:**

**1.        COVID-19 Risk Categorised Phenotype for Adult Immunosuppression:**

This is Phenotype 1. Conditions were allocated into Higher Risk or Lower Risk categories based on majority vote. Consensus (*) was achieved when ≥75% of panellists agreed with categorisation. Conditions are listed in descending order of votes for allocation into the Higher Risk category.

***Conditions at Higher Risk for Severe COVID-19 Outcomes within the Immunosuppressed Spectrum:***

Stem cell transplantation*

Multi-organ transplantation*

Solid organ transplantation*

Haematological malignancies*

Bone marrow transplantation*

Genetic disorders of the immune system (Primary Immunodeficiencies)*

AIDS-defining illness*

Underlying aberrant immunity*

Actively treated malignancies*

Untreated HIV*

Generalised malignancies (metastasis)*

Dialysis*

Islet transplantation

Chronic kidney disease

Down syndrome

Renal autoimmune diseases

Aplastic anemia

Rheumatological disorders

Solid tumours

Asplenia (functional or anatomic)

Pregnancy

Nephrotic syndrome

Neurologic autoimmune disorders

Sickle cell disease

HIV infection (all levels)

Haematologic autoimmune disorders

*Conditions at Lower Risk for Severe COVID-19 Outcomes within the Immunosuppressed Spectrum:*

Malnutrition

Type 2 Diabetes

Gastrointestinal autoimmune disorders

Burn injuries

Autoimmune skin diseases

Endocrine autoimmune diseases

Anorexia Nervosa

Cerebrospinal fluid leak

Opthamologic autoimmune diseases*

Well-managed HIV*

Cochlear implants*

Stunting*

Chronic stress*

Sleep deprivation*

 *achieved ≥75% panel agreement

**2.        COVID-19 Risk Stratified Phenotype for Adult Immunosuppression:**

This is Phenotype 2. Risk strata allocation is mutually exclusive; this ensures that higher risk patients within a certain population (e.g. AIDS-defining illness and untreated HIV) will be separated from other population terms (e.g. well-managed HIV and HIV infection [all levels]) and allocated into the highest risk strata that they are eligible. The strata below are listed in descending level of risk for severe COVID-19 outcomes.

1.        Transplantations

2.        Haematological and distributed malignancies

3.        Inborn errors of immunity, underlying aberrant immunity and acquired immunodeficiency syndrome

4.        Solid tumours and treatment protocols

5.        Kidney disease and treatment protocols

6.        Autoimmune and hyperinflammatory conditions

7.        Anemia and asplenia

8.        Well-managed HIV

9.        Barrier deficits

10.  Wider determinants of immunosuppression

Transplantations:

Stem cell transplantation

Multi-organ transplantation

Solid organ transplantation

Bone marrow transplantation

Islet transplantation

Haematological and distributed malignancies:

Haematological malignancies

Generalised malignancies (metastasis)

Inborn errors of immunity, underlying aberrant immunity and acquired immunodeficiency syndrome

Genetic disorders of the immune system (Primary Immunodeficiencies)

Underlying aberrant immunity

AIDS-defining illness

Untreated HIV

Solid tumours and treatment protocols

Solid tumours

Actively treated malignancies

Kidney disease and treatment protocols

Chronic kidney disease

Nephrotic disease

Dialysis

Autoimmune and hyperinflammatory conditions

Rheumatological disorders

Renal autoimmune diseases

Neurologic autoimmune disorders

Haematologic autoimmune disorders

Gastrointestinal autoimmune disorders

Autoimmune skin diseases

Endocrine autoimmune diseases

Opthamologic autoimmune diseases

Anemia and asplenia

Aplastic anemia

Asplenia (functional or anatomic)

Sickle cell disease

Well-managed HIV

Well-managed HIV

HIV infection (all levels)

Barrier defects

Cochlear implant

Cerebrospinal fluid link

Burn injuries

Wider determinants of immunosuppression

Malnutrition

Type 2 Diabetes

Anorexia Nervosa

Stunting

Chronic stress

Sleep deprivation

**DRAFT AFTER ROUND 2:**

**1.        COVID-19 Risk Categorised Phenotype for Adult Immunosuppression:**

In this round we have chosen to demarcate drug-managed HIV from the wider immunosuppressed phenotype to serve as an Active Control group for subsequent real-world investigations of COVID-19 vaccine benefit-risk in the immunosuppressed. This is based on consistent panel feedback that this group is inappropriate for inclusion in immunosuppressed cohorts and experiences no additional vulnerability for severe COVID-19 outcomes than the immunocompetent. We believe that using drug-managed HIV as an Active Control group in this way would address known issues comparing immunosuppressed with immunocompetent cohorts (vastly different demographics/ vaccine uptake; difficult to identify a truly immunocompetent cohort etc.), but we put this to the panel.

*High Risk for Severe COVID-19 Outcomes*

Bone marrow transplantation

Haematological Malignancies

Multi-organ transplantation

Solid Organ Transplantation

Stem Cell Transplantation

AIDS-defining Illness

Genetic Diseases (PIDs)

Untreated HIV

Generalised Malignancies (Metastasis)

Underlying Aberrant Immunity

Islet Transplantation

Dialysis

Renal Autoimmune Conditions

Aplastic Anaemia

Solid Tumours

*Moderate Risk for Severe COVID-19 Outcomes*

Chronic Kidney Disease (Late Stage)

Rheumatologic Autoimmune Conditions

Nephrotic Syndrome

Haematologic Autoimmune Conditions

Sickle Cell Disease

Asplenia

Neurological Autoimmune Conditions

Pregnancy

Gastrointestinal Autoimmune Conditions

Autoimmune Skin Conditions

Endocrine Autoimmune Conditions

Type 2 Diabetes

Burn Injuries

Malnutrition

Ophthalmologic Autoimmune Conditions

*Low Risk for Severe COVID-19 Outcomes*

Cerebrospinal Fluid Leak

Anorexia Nervosa

Cochlear Implants

Stunting

Active control: Drug-managed HIV

**2.**        **COVID-19 Risk Stratified Phenotype for Adult Immunosuppression:**

This is Phenotype 2. To ensure this phenotype is both clinically relevant and computerised medical record compatible, it considers the following factors when stratifying the patient spectrum: **1) vulnerability scoring, 2) panellist feedback, 3) population size and 4) common anatomical/ clinical denominators.**

Based on panel instruction, we have chosen to demarcate systemic from single site autoinflammatory conditions in this draft. While it is acknowledged that certain conditions included in the single site autoinflammatory strata have generalised effects on the body, they all have one primary source of damage or dysfunction (e.g. pancreas, thyroid).

For each strata, conditions are listed in descending levels of risk for severe COVID-19 outcomes. Once implemented, risk strata allocation is mutually exclusive; patients eligible for multiple levels are allocated to their highest risk strata.

1.        Transplantation

2.        Haematological or distributed malignancies

3.        Primary immunodeficiencies, unmanaged acquired immunodeficiencies & underlying aberrant immunity

4.        Solid tumours

5.        Renal disease & dialysis

6.        Systemic autoinflammatory conditions

7.        Asplenia & Anaemia

8.        Single site autoinflammatory conditions

9.        Anatomical barrier defects

10.  Wider determinants of immunosuppression

Active Control:

Drug-managed HIV

1. Transplantations:

Stem Cell Transplantation

Multi-Organ Transplantation

Bone Marrow Transplantation

Solid Organ Transplantation

Islet Transplantation

1. Haematological or distributed malignancies

Haematological Malignancies

Generalised Malignancies (metastasis)

1. Primary immunodeficiencies, unmanaged acquired immunodeficiencies & underlying aberrant immunity

Genetic disorders of the immune system (Primary Immunodeficiencies)

AIDS-defining Illness

Underlying aberrant immunity

Untreated HIV

Down syndrome

1. Solid tumours

1. Renal Disease and Dialysis

Dialysis

Renal Autoimmune Conditions

Chronic Kidney Disease (Late Stage)

Nephrotic Syndrome

1. Systemic immune-mediated autoinflammatory conditions

Rheumatological autoimmune diseases

Haematological autoimmune diseases

1. Anaemia & asplenia

Aplastic anaemia

Sickle cell disease

Asplenia (functional or anatomic)

1. Single site immune-mediated autoinflammatory conditions

Neurological autoimmune conditions

Gastrointestinal autoimmune conditions

Autoimmune skin conditions

Endocrine autoimmune conditions

Ophthalmological autoimmune conditions

1. Anatomical Barrier Defects

Burn Injuries

Cerebrospinal Fluid Leak

Cochlear Implant

1. Wider determinants of immunosuppression

Pregnancy

Type 2 Diabetes

Malnutrition

Anorexia Nervosa

Stunting

Active Control Group:

Drug-Managed HIV

**DRAFT AFTER FINAL DISCUSSION GROUPS:**

**1.        COVID-19 Risk Categorised Phenotype for Adult Immunosuppression:**

Upon in-person consultation with the Panel, it became clear that there is widespread discomfort with converting the outcomes of binary ‘higher vs lower risk immunosuppressed’ consensus building into a three-part, linear model of risk categorisation to be incorporated into disease surveillance data flows (previously ‘Phenotype 1’). It cannot be inferred that conditions where consensus wasn’t reached represents ‘moderate’ risk, for example. As such, this data will be reported in our manuscript for clinical interest, but not formalised into a digital phenotype at this moment in time. Instead, a banded approach will be taken to the second phenotype, enabling both granular divisions of immunosuppressed disease surveillance and three-part risk categorisation to support clinical decision-making (subject to real-world validation).

*Consensus Higher COVID Risk Immunosuppressed*

Bone marrow transplantation

Haematological Malignancies

Multi-organ transplantation

Solid Organ Transplantation

Stem Cell Transplantation

AIDS-defining Illness

Genetic Diseases (PIDs)

Untreated HIV

Generalised Malignancies (Metastasis)

Underlying Aberrant Immunity

Islet Transplantation

Dialysis

Renal Autoimmune Conditions

Aplastic Anaemia

Solid Tumours

*No Consensus Reached*

Chronic Kidney Disease (Late Stage)

Rheumatologic Autoimmune Conditions

Nephrotic Syndrome

Haematologic Autoimmune Conditions

Sickle Cell Disease

Asplenia

Neurological Autoimmune Conditions

Pregnancy

Gastrointestinal Autoimmune Conditions

Autoimmune Skin Conditions

Endocrine Autoimmune Conditions

Type 2 Diabetes

Burn Injuries

Malnutrition

Ophthalmologic Autoimmune Conditions

*Consensus Lower COVID Risk Immunosuppressed*

Cerebrospinal Fluid Leak

Anorexia Nervosa

Cochlear Implants

Stunting

**The DESTINIES Phenotype: ‘*Adult immunosuppression, classified by COVID-19 vulnerability’***

Levels are presented in descending vulnerability to severe COVID-19 outcomes compared to the immunocompetent baseline; conditions within each level (operationalised below) are also presented in descending vulnerability. Once implemented, risk level allocation is mutually exclusive; medical records eligible for multiple levels are only allocated to their highest risk level. **Risk banding is currently set at 1-3 (Highest excess vulnerability), 4-7 and 8-10 (Lowest excess vulnerability), though this will be finalised after real-world implementation.**


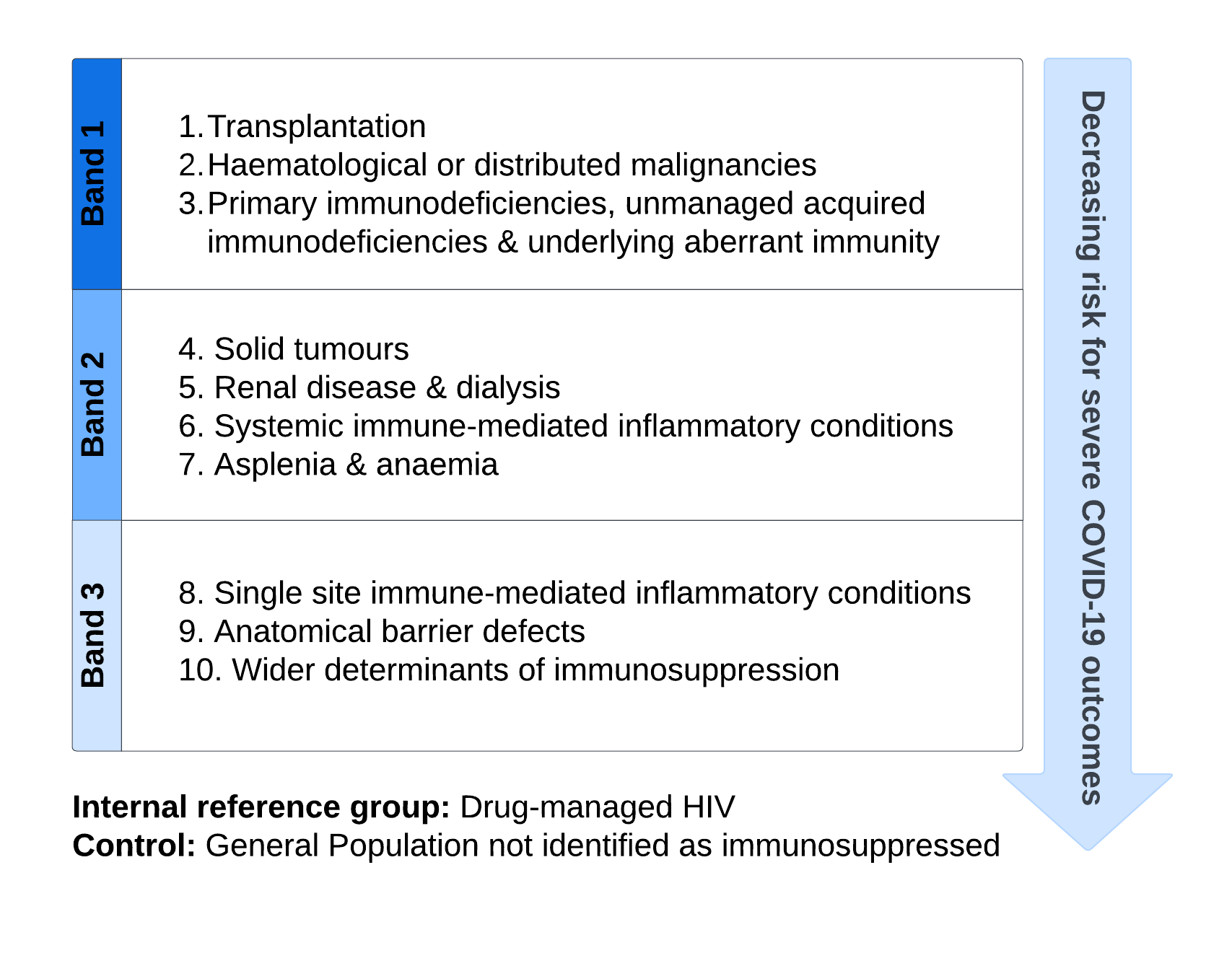


1. Transplantations:

Hematopoietic stem cell transplantation (Bone Marrow/ Stem Cell transplantation)

Multi-Organ Transplantation

Solid Organ Transplantation

Islet Transplantation

1. Haematological or distributed malignancies

Haematological Malignancies

Generalised Malignancies (metastasis)

*Medical records with remission codes excluded*

1. Primary immunodeficiencies, unmanaged acquired immunodeficiencies & underlying aberrant immunity

Genetic disorders of the immune system (Primary Immunodeficiencies: e.g. SCID)

AIDS-defining Illness (HIV-related conditions including tuberculosis, Kapsi’s sarcoma, candidiasis, histoplasmosis, isosporiasis, recurrent pneumonia etc.) & Unmanaged HIV

Underlying aberrant immunity (e.g. graft-vs-host disease, graft rejection, absent or incomplete immune reconstitution, neutropenia ANC, lymphopenia ALC, secondary hypogammaglobinaemia etc.)

Down syndrome

1. Solid tumours

*Medical records with remission codes excluded*

1. Renal Disease & Dialysis

Dialysis

Renal Autoimmune Conditions (e.g. Lupus nephritis, Glomerulonephritis, IgA nephropathy etc.)

Chronic Kidney Disease (Late Stage, 4+)

Nephrotic Syndrome

*Excluding Chronic Kidney Disease < Stage 4*

1. Systemic immune-mediated inflammatory conditions

Rheumatological autoimmune diseases (e.g. systemic lupus erythematosus, rheumatoid arthritis, Sjögren's syndrome, idiopathic inflammatory myopathies, ankylosing spondylitis, vasculitides, gout etc.)

Haematological autoimmune diseases (e.g. Evan’s syndrome, immune thrombocytopenic purpura, antiphospholipid syndrome, autoimmune neutropenia, VEXAS etc.)

Gastrointestinal autoimmune conditions (e.g. celiac disease, Crohn’s disease, ulcerative colitis, autoimmune gastritis, microscopic colitis, autoimmune pancreatitis, autoimmune hepatitis etc.)

Neurological autoimmune conditions (e.g. multiple sclerosis, myasthenia gravis, neuromyelitis optica etc.)

1. Anaemia & asplenia

Severe anaemia (e.g. aplastic anaemia, pernicious anaemia, autoimmune haemolytic anaemia etc.)

Sickle cell disease

Asplenia (functional or anatomic)

*Generic/ mild cases of anaemia excluded*

1. Single site immune-mediated inflammatory conditions

Autoimmune skin conditions (e.g. psoriasis, dermatitis herpetiformis, vitiligo, pemphigus, scleroderma, dermatomyositis etc.)

Endocrine autoimmune conditions (e.g. Type 1 diabetes mellitus, Hashimoto’s thyroiditis, Graves' disease, Addison’s disease etc.)

Ophthalmological autoimmune conditions (e.g. Uveitis, Graves’ ophthalmopathy etc.)

1. Anatomical Barrier Defects

Severe burn Injuries

Cerebrospinal Fluid Leak

Cochlear Implant

*Mild/ moderate burns excluded (< Stage 3)*

1. Wider determinants of immunosuppression

Pregnancy (final trimester)

Type 2 Diabetes

Malnutrition (operationalised as anorexia nervosa & stunting)

*Excluding pregnancies earlier than 3^rd^ trimester*

Internal reference group: Drug-managed HIV

Control: General Population not identified as immunosuppressed
